# The *Lancet Global Health* Commission on financing primary health care: putting people at the centre

**DOI:** 10.1016/S2214-109X(22)00005-5

**Published:** 2022-04-04

**Authors:** Kara Hanson, Nouria Brikci, Darius Erlangga, Abebe Alebachew, Manuela De Allegri, Dina Balabanova, Mark Blecher, Cheryl Cashin, Alexo Esperato, David Hipgrave, Ina Kalisa, Christoph Kurowski, Qingyue Meng, David Morgan, Gemini Mtei, Ellen Nolte, Chima Onoka, Timothy Powell-Jackson, Martin Roland, Rajeev Sadanandan, Karin Stenberg, Jeanette Vega Morales, Hong Wang, Haja Wurie

**Affiliations:** a Department of Global Health and Development, London School of Hygiene and Tropical Medicine, London, UK; b Department of Health Services Research and Policy, London School of Hygiene and Tropical Medicine, London, UK; c Breakthrough International Consultancy, Addis Ababa, Ethiopia; d Heidelberg Institute of Global Health, University Hospital and Faculty of Medicine, University of Heidelberg, Heidelberg, Germany; e National Treasury, Pretoria, South Africa; f Results for Development, Washington DC, USA; g Bill & Melinda Gates Foundation, New Delhi, India; h UNICEF, Iraq Country Office, Baghdad, Iraq; i World Health Organization, Kigali, Rwanda; j World Bank, Washington DC, USA; k China Center for Health Development Studies, Peking University, Beijing, China; l Health Division, The Organisation for Economic Co-operation and Development, Paris, France; m Abt Associates, Dar es Salaam, Tanzania; n Department of Community Medicine, University of Nigeria, Enugu, Nigeria; o Department of Public Health and Primary Care, University of Cambridge, UK; p Health Systems Transformation Platform, New Delhi, India; q World Health Organization, Geneva, Switzerland; r Pronova Technologies, Santiago, Chile; s Bill & Melinda Gates Foundation, Seattle, WA, USA; t College of Medicine and Allied Health Sciences, University of Sierra Leone, Freetown, Sierra Leone

## Executive summary

The COVID-19 pandemic has brought the need for well-functioning primary health care (PHC) into sharp focus. PHC is the best platform for providing basic health interventions (including effective management of non-communicable diseases) and essential public health functions. PHC is widely recognised as a key component of all high-performing health systems and is an essential foundation of universal health coverage.

PHC was famously set as a global priority in the 1978 Alma-Ata Declaration. More recently, the 2018 Astana Declaration on PHC made a similar call for universal coverage of basic health care across the life cycle, as well as essential public health functions, community engagement, and a multisectoral approach to health. Yet in most low-income and middle-income countries (LMICs), PHC is not delivering on the promises of these declarations. In many places across the globe, PHC does not meet the needs of the people—including both users and providers—who should be at its centre. Public funding for PHC is insufficient, access to PHC services remains inequitable, and patients often have to pay out of pocket to use them. A vicious cycle has undermined PHC: underfunded services are unreliable, of poor quality, and not accountable to users. Therefore, many people bypass primary health-care facilities to seek out higher-level specialist care. This action deprives PHC of funding, and the lack of resources further exacerbates the problems that have driven patients elsewhere.

### Focus on financing

Health systems are fuelled by their financing arrangements. These arrangements include the amount of funding the system receives, the ways funds are moved through the system to frontline providers, and the incentives created by the mechanisms used to pay providers. Establishing the right financing arrangements is one crucially important way to support the development of people-centred PHC. Improving financing arrangements can drive improvements in how PHC is delivered and equip the system to respond effectively to evolving population health needs. Thus attention should be paid simultaneously to both financing and service delivery arrangements.

In this report, the *Lancet Global Health* Commission on financing PHC argues that all countries need to both invest more and invest better in PHC by designing their health financing arrangements—mobilising additional pooled public funding, allocating and protecting sufficient funds for PHC, and incentivising providers to maintain the health of the populations they serve—in ways that place people at the centre and by addressing inequities first.

### Financing is political

Answering the question of how to make these changes goes far beyond technical considerations. Fundamentally shifting a health system's priorities—away from specialist-based and hospital-based services and towards PHC—involves political choices and creates numerous political challenges. Successfully reorienting a system towards PHC requires savvy political leadership and long-term commitment, as well as proactive, adaptable strategies to engage with stakeholders at all levels that account for the social and economic contexts. Therefore, this report addresses both technical and political economy considerations involved in strengthening financing for PHC.

### Spending more and spending better on PHC

Despite broad recognition of the importance of PHC, there is no global consensus on what exactly constitutes PHC. This makes it challenging to measure and report on levels of expenditure on PHC. In this Commission we define PHC as a service delivery system or platform, together with the human and other resources needed for it to function effectively. We found that LMICs spend far too little on PHC to provide equitable access to essential services and that much of the (significant) variation in PHC spending levels across countries is explained by national income levels, although there is variation in the amount of government resources allocated to PHC at any given level of economic development. Furthermore, at every level of PHC spending, there is substantial variation in performance, suggesting that we need to spend better as well as spending more.

In this Commission, we analysed provider payment methods and found that the sources of PHC expenditure remain fragmented and overly reliant on out-of-pocket payments. Population-based provider payment mechanisms, such as capitation, should be the cornerstone of financing for people-centred PHC. However, these mechanisms are rare in LMICs, where input-based budgets are standard practice. Furthermore, many features of primary health-care organisation that are necessary for population-based payment strategies (such as empanelment, registration, and gatekeeping) are absent in LMICs.

Redressing these limitations to improving financing PHC is urgent, as new challenges continue to arise. As in other parts of the health sector, PHC will continue to become more integrated, digitally-driven, and pluralistic; therefore, PHC financing arrangements also need to evolve to support, drive, and guide these changes to better meet human needs.

The Commission takes the position that progressive universalism should drive every aspect of PHC. That means putting the rights and needs of the poorest and most vulnerable segments of a population first. This requires unwavering ethical, political, and technical commitment and focus. Together with this overarching principle, we identified four key attributes of people-centred financing arrangements that support PHC.

(1) Public resources should provide the core of primary health-care funding. Revenue-raising mechanisms should be defined based on the ability to pay and be progressive. Out-of-pocket payments must be reduced to levels where they are no longer a financial barrier to accessing needed care, impoverish households, or push households deeper into poverty. In most LMICs, this level of public funding for PHC can only be generated through increased allocations to PHC from general tax revenue, and therefore requires an expansion of countries' taxation capacities. In low-income countries, more development assistance will be needed to expand the resource envelope for PHC.
(2) Pooled funds should be used to allow all people to receive PHC that is provided free at the point of use. Only once universal coverage with PHC is achieved should pooled resources be extended to cover other entitlements. In this way, PHC can help fulfil the promise of universal health coverage.
(3) Resources for PHC should be allocated equitably (across levels of service delivery and geographic areas) and protected as they flow through the system to frontline providers. Countries should deploy a set of strategic resource allocation tools (including a needs-based per-capita resource allocation formula and effective public financial management tools) to match primary health-care funding with population needs and ensure these resources reach the frontline, and prioritise the poorest and most vulnerable people.
(4) Payment mechanisms for primary health-care providers should support allocation of resources based on people's health needs, create incentive environments that promote PHC that is people-centred, and foster continuity and quality of care. To achieve these goals, a so-called blended provider payment mechanism with capitation at its core is the best approach to paying for PHC. Capitation should form the core of the primary health-care financing system because it directly links the population with services. Combining capitation with other payment mechanisms, such as performance-based payments for specific activities, enables additional objectives to be achieved.

Each country is at a different point along its path towards the goal of effective financing for PHC. The four attributes outlined both represent goals and present a guide for working towards those goals. This Commission recognises that, depending on the context, the evolution of an effective primary health-care financing system in some countries might occur through incremental changes, whereas others can implement comprehensive reforms. Improving PHC financing can occur in response to bottom-up advocacy, top-down policy or, most likely, through a combination of grassroots and technocratic approaches. Political, social, and economic factors are therefore as important as technical design elements when it comes to enacting efficient and equitable primary health-care financing reform. Changing the ways in which PHC is financed requires support from a wide range of stakeholders, and deliberate political strategies, to determine and then stay the course. The change also requires good information about PHC resource levels and flows so that this reorientation can be effectively managed and monitored.

In this Commission, we provide five recommendations.

(1) People-centred financing arrangements for PHC should have public resources provide the bulk of primary health-care funding; pooled funds cover primary-health care, enabling all people to receive PHC that is provided free at the point of service use; resources for PHC are allocated equitably across levels of service delivery and geographic areas, and are protected so that sufficient resources reach frontline primary health-care service providers and patients; and primary health-care provider payment mechanisms support the allocation of resources based on people's health needs, create incentive environments that promote PHC that is people centred, foster continuity and quality of care, and remain flexible enough to support rapidly changing service delivery models.
(2) Spending more and spending better on PHC requires a whole-of-government approach involving all ministries whose remit interacts with health and requires the support of civil society. Key actors and stakeholders should be involved in designing and implementing financing arrangements for PHC that are people-centred. Although the specifics will vary depending on the national context, there are important roles and responsibilities for ministries of health, ministries of finance, local government authorities, communities and civil society groups, health-care providers and organisations, donors, and technical agencies.
(3) Each country should plot out a strategic pathway towards people-centred financing for PHC that reflects the attributes outlined above, including investments in supporting basic health system functions. Technical strategies should be underpinned from the outset by analysis of the political economy.
(4) Global technical agencies should reform the way primary health-care expenditure data are collected, classified, and reported to enable longitudinal and cross-country analyses of achievement of key primary health-care financing goals.
(5) Academic researchers, technical experts, and policy makers, among others, should pursue a robust research agenda on financing arrangements for PHC that place people at the centre to support achievement of key primary health-care financing goals.

## Introduction

Primary health care (PHC) is a key component of all high-performing health systems,^1^ an essential foundation for universal health coverage (UHC), and a prerequisite for meeting the Sustainable Development Goals. It is a pathway to achieving good health at low cost^2^ by providing essential and cost-effective health interventions, including health promotion; maternal, newborn, and child health care; immunisations; and treatment for common illnesses across the life course. As the global burden of non-communicable diseases increases, PHC is emerging as the locus of both prevention and the coordination of life-long management of chronic conditions. PHC also has an important role in providing essential public health functions, including responding to epidemic diseases such as the COVID-19 pandemic.

When successfully delivered, PHC serves as a key vehicle for fulfilling governmental and societal commitments. For example, primary health-care expansion improves equity when its services reach vulnerable segments of the population.^3^ Because primary health-care services are provided where people live and work,^4^ and because PHC focuses on population health, it can address many determinants of health that underpin various sources of vulnerability.^5^ PHC can protect households' financial wellbeing by fostering good health and reducing the risks of disease among breadwinners, caregivers, and other family members, and by averting the need for expensive secondary and tertiary health care.^6^ In fragile states and conflict-affected settings, primary health-care services can help build trust in the health system—and in the government it represents.^7^

A convincing economic case for PHC has been made repeatedly. Most of the available evidence comes from high-income countries. In these contexts, it has been shown that by providing key services at the lowest appropriate level of the health system, PHC can decrease the need for unnecessary hospital admissions, prevent avoidable readmissions, and limit inappropriate use of emergency departments.^8^ In low-income to middle-income countries (LMICs), an expanding body of evidence shows the cost-effectiveness of many interventions that are typically delivered through PHC. Indeed, a 2018 analysis classified 198 (91%) of 218 essential UHC interventions as PHC^9^ and another report estimated that up to 75% of the projected health gains from the SDGs could be achieved through PHC.^10^ Expanding a core set of integrated interventions for women's and children's health (narrower than PHC) is calculated to generate economic and health benefits in low-income countries valued at 7·2 times more than the costs; the value increases to 11·3 in lower-middle-income countries.^11^ A study of 67 LMICs projected that investing in PHC over the period from 2020 to 2030 would avert up to 64 million deaths.^10^

There is also a strong case for public investments in common goods for health, including public goods^12^ (which, in the economic sense, are services and functions that are both non-rival and non-exclusive), and in functions that generate strong positive externalities. These goods, which include the essential public health functions in PHC, require public funding as they are otherwise subject to market failure.

Yet despite its fundamental importance and incredible promise, PHC is not doing well in many countries, especially LMICs. The global community first proclaimed its commitment to multisectoral and integrated PHC in the 1978 Alma-Ata Declaration. However, this commitment was quickly derailed, with funding and technical support flowing instead into vertical and disease-specific programmes.^13^ Despite periodic attempts to refocus on PHC, vertical programmes and hospital-based and specialist-based care models have regularly been prioritised over PHC. Funding for PHC is generally insufficient, access to primary health-care services remains inequitable, services are of inadequate quality, and patients often have to make out-of-pocket payments to use them. Health-care worker shortages persist, particularly in rural areas where the need is often greatest, and in many countries supplies of medicines, equipment, and other necessary commodities are grossly inadequate.^6^

This situation reinforces a cycle of neglect of PHC: when primary health-care services are unreliable, of poor quality, and not accountable to system users, it leads to poor uptake and low levels of trust in community-level health care. Users choose to bypass primary health-care services, which then receive even fewer resources. To successfully provide PHC at community level, national and local health-care systems need to be reimagined and restructured, beginning with placing the needs and preferences of people (including the intended users and providers) at the centre of the system design.^6^, ^14^

Health financing arrangements provide the fuel for health systems: they establish the amount of resourcing available and the way in which risks are shared among those who are ill and those who are well, the ways that funds flow through the system to frontline providers, and the payment systems that create incentives for providers. Together, these arrangements shape the equity, effectiveness, and efficiency of PHC.

This report focuses on how to get the financing arrangements right to serve and fuel effective, efficient, and equitable PHC service delivery. As will be discussed throughout the report, establishing the right financing arrangements for effective and equitable PHC can both support and drive other necessary transformations. This Commission contends that health financing arrangements for PHC—how to mobilise sufficient resources to support PHC objectives, how to ensure that resources reach frontline providers in ways that align with PHC objectives, and how to design financial incentives that encourage the delivery of, and access to, high-quality, equitable, integrated and efficient PHC—should be centred on people, and focused on equity. Panel 1 presents descriptions of two key terms that are used throughout the report: health financing functions and health financing arrangements.

Panel 1 **Health financing functions and arrangements**

Three core health financing functions are mentioned throughout the report:

• Mobilisation of funds: the collection of revenue (from taxes, insurance contributions, user fees, donations, or other means) that is used to pay for delivery of health services. Resource mobilisation is addressed in detail in section 3.
• Pooling: accumulating prepaid funds (such as social security contributions, taxes, or health insurance premiums) to pay for health services for a group of people. Pooling is addressed in section 3.
• Purchasing: the mechanisms by which mobilised and pooled funds are transferred to providers who deliver health services. Purchasing involves three elements: specifying what services will be purchased (often called the benefit package), identifying which providers are eligible to provide these services, and defining the set of arrangements through which providers are contracted to provide the services. How providers are paid to provide primary health care (PHC) is the focus of section 5.

We refer in the report to a number of different ways of paying PHC providers:

• A line-item budget is when providers are given prospectively a fixed amount of funds to cover specific line items, such as medicines and utilities, for a period (usually a year).
• A fee-for-service payment is when providers are reimbursed for each individual service provided.
• A capitation payment is when providers are given a fixed per-person payment, determined and paid in advance, to deliver a defined set of services to each enrolled individual for a specified period of time.
• A pay-for-performance system is when providers are given bonus payments (or penalties) for achieving service coverage or quality targets.

We use the broader term health financing arrangements to refer to both the core health financing functions and the ways in which they are organised and interact. These arrangements include the public financial management processes through which resources flow to frontline providers. Throughout the report we pay particular attention to:^15^

• Budget formulation: the process of determining, soliciting, and securing sufficient public funding for PHC and the health system overall.
• Resource allocation: the process of assigning available resources to specific uses (in this case, to PHC).
• Budget execution: how the funds budgeted for services flow through the public system to providers.

In this Commission, we aimed to present new evidence on levels and patterns of global expenditure on PHC (throughout the report, the terms expenditure and spending are used interchangeably), including describing how PHC is currently organised and paid for; analyse key technical and political economy challenges faced in financing PHC; identify areas of proven or promising practices that effectively support PHC across the key health financing functions; and identify actionable policies to support LMICs in raising, allocating, and channelling resources in support of the delivery of effective, efficient, and equitable PHC that is people centred. Section 1 provides a general introduction to PHC policy and challenges. It then characterises global and national challenges, as well as opportunities, related to financing PHC. Section 2 describes the current financing landscape for PHC, detailing existing patterns of expenditure, provider payment, and related organisational features. Section 3 elaborates on mobilising sufficient resources for health through progressive means and then pooling resources to enable cross-subsidisation between those who are ill and those who are well. Section 4 focuses on how to ensure that resources mobilised for health are allocated to PHC, and emphasises the importance of engaging with the multiple budget tools available to Ministries of Health to ensure that resources reach frontline providers. Section 5 highlights the importance of structuring incentives for PHC providers so that they are motivated to provide PHC that is people centred, and proposes a strategic pathway of steps that countries can take to establish appropriate incentives. Section 6 describes the importance of, and notes strategies for, addressing the political economy of financing PHC. Finally, section 7 presents a synthesis of the vision for people-centred financing arrangements for PHC, summarises possible pathways for working towards this vision, and provides recommendations and proposes actions for different stakeholders committed to supporting LMICs to spend more—and to spend better—on PHC. It is the Commission's hope that this report will serve as a resource to policy makers around the world who are committed to this crucial endeavour.

We prepared this Commission through an extensive process of study and debate on good and promising practices in financing PHC. The 22 expert members, representing 19 nationalities, have amongst them experience working in national governments, technical agencies, bilateral and multilateral donors, universities, and independent think tanks. Assisted by a technical team based at the London School of Hygiene & Tropical Medicine, London, Commissioners drew on the following sources of evidence: case studies prepared by national consultants on innovations in PHC financing in seven LMICs (Brazil, Chile, China, Ethiopia, Ghana, India, and Philippines) and three high-income countries (Estonia, Finland, and New Zealand); a compilation of Organisation for Economic Co-operation and Development (OECD) and WHO health expenditure data to conduct expenditure analysis; a new survey of PHC organisation and provider payment in LMICs; literature reviews, including systematic and scoping reviews, of existing knowledge on financing PHC; and an expert roundtable on digital technologies and PHC financing. Additional publications based on these products from the Commission are available on the Commission's website.

## Section 1: Financing primary health care in the 21st century—challenges and opportunities

### Defining PHC

In different contexts, PHC has been operationalised in different ways: as an approach to the delivery of health care that reorients the system away from hospitals and specialist care to practitioners working at community-level outpatient facilities; as a coordination mechanism which links primary care, community care, specialised care, wider public health interventions, and long-term care services;^16^ as a package of health services, often defined using cost-effectiveness as a primary criterion; as a service delivery level or platform, together with the human and other resources needed for it to function effectively; or as a system which combines a platform, a service package, and an approach that emphasises an orientation to meeting the needs of the population.

For the purposes of the Commission's health financing analyses, we found it necessary to link service delivery arrangements and orientations of PHC with the way resources are directed through the financing system to reach frontline providers. Resources typically flow to service delivery platforms. For this reason, PHC as a platform is our favoured operational definition of PHC. It typically includes both community-level and first-level health care. While it is true that some PHC services might be provided in hospital outpatient departments, it is the contention of this Commission that, over time, countries should aim to shift most PHC services out of hospitals to the appropriate community-level or first-level platforms where they can be delivered cost-effectively.

PHC is being transformed by new technologies that have the potential to overcome persistent challenges and radically change how people engage with health services. For example, digital technologies are streamlining procurement of commodities, improving supply chains, supporting health-care providers' adherence to clinical guidelines, and enabling tracking of patients who would otherwise be lost to follow-up.^17^, ^18^ New mobile and telemedicine technologies are helping patients to remotely access health information, medical advice, and their own health data. These technologies might help patients and their families to take greater responsibility for their own health and enable new, more horizontal relationships between patients and providers. Similarly, opportunities to pay insurance premiums digitally, such as via mobile phones, may help to mobilise additional financing for health. Technology-driven transformations bring some risks, including increasing health inequalities and fragmenting financing and delivery of care. However, they also offer avenues for making PHC more convenient, accessible, affordable, and high quality.

PHC will continue to evolve. For this evolution to fulfil the potential of PHC, health-care providers must expand their areas of focus and develop new skills, and health systems must develop new ways of delivering services across the life course, including incorporating preventive and supportive services. Innovations such as new digital and telehealth platforms must be deployed to support individuals and their families to manage their own health. Governments and communities must recognise and foster the role of PHC in essential public health functions, and PHC must engage with individuals and the wider community to co-produce forms of delivery that will meet people's needs. Appropriate use of technology will be key to support those delivering and those using services—eg, enabling task shifting between different cadres of health workers and delivering care that is flexible and closer to people's homes.

### COVID-19: changing the context and highlighting lessons for PHC

The COVID-19 pandemic has brought the need for PHC that is well financed into sharp focus in several ways:

• It underscores the relationship between health and the economy. In particular, it has highlighted that failing to invest in health, including PHC, can have dramatic economic consequences.
• Countries with stronger PHC systems were able to respond faster and more effectively to the pandemic.^19^, ^20^, ^21^ For example, Japan, Vietnam, and South Korea were better prepared to carry out COVID-19 surveillance because they were able to capitalise on existing public health capacity for contact tracing.^22^ Close partnerships between multidisciplinary PHC providers and local governments allowed rapid responses by reassigning roles while still maintaining other public health services, as seen in France^23^ and Catalonia, Spain.^24^ This shows that PHC systems provide a foundation for effective management of health crises.
• The PHC system is a good platform for public health measures to control infectious diseases. The pandemic brought renewed attention to the vital importance of common goods for health,^25^ including the essential public health functions that are a component of PHC. Essential public health functions include surveillance systems, test-and-trace systems, quarantine functions, and vaccination.
• Going forward, COVID-19 can only be overcome through action at the PHC level. For example, COVID-19 vaccinations will be provided through PHC platforms as provision shifts from a vertical campaign mode to a routine service. Management of mild-to-moderate illnesses related to COVID-19 will also be through PHC.
• The COVID-19 pandemic has accelerated service delivery changes that were already underway. In particular, health care has rapidly adjusted to incorporate remote consultations, ramped-up support for home care, task shifting to lower-level cadres and structures, and expanded digital monitoring of health status, among others.
• Above all, by highlighting the structural inequalities that exist within and across countries, the COVID-19 pandemic has emphasised the need to work for equity, solidarity, and social justice for all—these principles are central to the PHC approach.

Many of these lessons were highlighted in previous health emergencies, such as the 2014 Ebola outbreak in West Africa. However, the global scale of COVID-19, with its accompanying global and national responses, have conclusively shown that health issues can be made a top priority and that rapid changes to health systems and financing are possible. Governments and international funders alike created new flexibility in health financing arrangements, including rapid budget reallocations, mobilisation of new funds, and use of flexible purchasing arrangements. Some of these new arrangements should be retained and expanded. However, they have also exposed new areas of financial risk and vulnerability, as well as highlighting the need to continually focus on transparency and accountability.^26^

COVID-19 has shown that the need for well-financed, well-functioning PHC has never been greater. Yet many aspects of the pandemic response have instead led to a greater concentration of resources on hospital care, vaccines, and other so-called silver-bullet approaches^27^, ^28^ instead of prioritising basic public health interventions such as test-and-trace, disease surveillance, and population-based preventive measures.

This presents real risks to PHC financing and delivery. The financing requirements of the response to COVID-19 (both by the health system and in the economic response) have placed unprecedented pressure on government budgets, while spending capacity has decreased due to declines in revenue and borrowing.^29^ For example, the immediate financing needs for additional funding for COVID-19 prevention, treatment and surveillance in sub-Saharan African countries were estimated at about 3% of gross domestic product (GDP), or US$53 billion.^30^ At the same time, the International Monetary Fund estimated that economies around the world contracted in per-capita terms by an average 5·9% in 2020 as a result of COVID-19,^31^ driving an untold number of households into poverty and reducing their ability to pay for health care.

Spending on routine health services has fallen in many countries^32^ and generating more public resources for PHC will be challenging under conditions of fiscal restraint. In 90% of 105 countries surveyed by WHO, the pandemic badly disrupted many essential services that were not directly related to COVID-19, particularly mental health and reproductive, maternal, neonatal and child health care.^33^ Among 22 low-income countries, ten (45%) reported disruptions in at least 75% of essential services—this represents far more disruption than was reported in LMICs (30%) and upper-middle and high-income countries (8%).^34^ In the Democratic Republic of the Congo, for example, by October, 2020, up to 33% of the health budget had been redirected to the COVID-19 emergency response.^33^ Detailed accounts of the effect of COVID-19 on health financing in two other countries in sub-Saharan Africa, Sierra Leone, and South Africa, can be found in the appendix (p 2).

The COVID-19 pandemic has thus shifted the landscape of possibilities for financing people-centred PHC in particular and health more broadly. Although it generated some new opportunities, it also created vast new challenges—and provided a glimpse of the potential havoc that future crises (health and otherwise) can create, and the consequences of not prioritising equity.^35^ Health financing systems need to be resilient to allow the surge capacity needed to respond to shocks while maintaining access to essential services.

### Determining PHC packages

To examine and operationalise financing arrangements for PHC requires clarity on what services are being financed. The PHC package can be conceptualised at three levels. At the highest level, each government and national health system must articulate its own vision for comprehensive PHC that addresses its population health needs. Fulfilling a stated vision requires drawing on resources from both public and private sources. The Alma-Ata Declaration's vision of PHC also encompasses contributions from other sectors to address social determinants of health. In this report we focus on choices made within the health budget but recognise the need to identify mechanisms for securing contributions from outside the health sector, including education, water, and sanitation. At the benefit package level, each government and health system must identify which services it can afford to provide either for free or with partial coverage. In many low-income settings, external funds will be needed to augment government financing. At the provider payment level, each health system must determine which services it will pay providers for, at what level of payment, and via which provider payment mechanism (see section 5). Vertical programmes that provide some PHC services might be excluded from this payment system.

The specific PHC package that is financed and delivered in any particular setting will be determined by a country's (or region's) fiscal capacity, population health needs, and political decisions about priorities. It must include both population-based essential public health functions and personal health services. Cost-effectiveness criteria should inform these choices, but a pragmatic approach is needed when combining services at an operational, or service delivery platform, level.

### PHC finance and delivery are linked

Directing resources to certain levels, structures, and providers makes it possible for them to function—and it also strengthens them so they can continue pulling and absorbing resources for appropriate and effective care. Conversely, inappropriate health financing arrangements can constrain effective care, and drive users to seek services that should be offered as PHC from higher levels of the system or from unregulated providers.

Getting financing functions right is important. But numerous countries' experiences have shown that PHC financing reforms work best when the organsation of PHC delivery is improved at the same time. This might be done by, for example, creating new cadres of health worker, or by incentivising multidisciplinary team approaches. Organisational reforms both enable the absorption of additional resources and make PHC more people centred. We therefore argue that countries need to address financing levels of PHC, financing arrangements, and delivery structures at the same time.

### Financing is political

Designing financing arrangements is more than just a technical challenge—it also involves choices that are inherently political, in the broad sense of the term. Political, socioeconomic, and cultural conditions are part of the context in which PHC financing reforms take place and are integral to whether and how reform occurs.

Increasing the allocation of resources to health might require taking resources away from other sectors. It might also necessitate changing the roles of hospitals so they are more supportive of PHC and share responsibility for population health. Strengthening financing arrangements for PHC to better reach frontline providers and communities should mean that as resources increase, the relative distribution of resources and power will favour PHC providers compared to hospitals and specialists to ensure improvements in PHC services. Such shifts are complex because they run counter to political pressures that often favour investing in readily visible improvements, such as building facilities and reducing hospital waiting times. Whether changes are instituted as top-down radical changes or via bottom-up incremental modifications, they require shifts in power and influence at all levels.

In all cases, leaders pushing to change PHC financing must attend to the political economy of PHC financing reform (including making the political case for change and building the coalitions to enact it). Political economy considerations are woven throughout the report, and section 6 specifically addresses political economy analysis for PHC financing reform.

### Building on strong health system foundations

Financing is only one, albeit an important, element of well-functioning health systems. It strongly influences, and is influenced by, other key health system building blocks. Having these other building blocks in place is crucial. These include governance arrangements that support delivery of people-centred PHC; a health workforce that is trained and supported to provide high-quality care; data, monitoring, evaluation and learning systems to capture and disseminate accurate health and spending information; functioning procurement and distribution supply chains for medicines and other commodities; and public finance management systems supporting every aspect of financing and delivery.

### Financing community health and community health workers

The Commission has not differentiated between PHC and community health—an area that of late attracts substantial donor funding, particularly for the deployment of community health workers.^36^ Indeed, community health systems can be considered an advanced form of PHC implementation, where care takes place in the communities where people live and work. These platforms also present additional opportunities for deployment of new technologies. Although community health is arguably more focused on accountability and health service delivery in the context of the community (however defined), it still depends on trained staff, supplies, infrastructure, administrative processes, and integration with higher levels of care. Community health cannot exist outside the systems underpinning PHC, and must ultimately be financed on budget through the same mechanisms recommended elsewhere in this Commission. Although some governments or donor-funded programmes might separate community health activities in the context of PHC,^36^ others will integrate community health workers with their clinical personnel. What is most important is recognising the shared goal of universal access to primary care services and essential public health functions also prioritised by this Commission.

A key financing policy choice is whether and how to pay community health workers. Securing sustainable financing for community health worker programmes can be a challenge, particularly as in many countries these rely on external funding.^37^ Debates about paying community health workers typically focus on the trade-offs between reliance on volunteerism underpinned by intrinsic motivation of volunteers and the need to recognise and remunerate work fairly (and in doing so, addressing gender disparities, as most of the community health worker workforce is female).^38^, ^39^ Section 5 includes a brief summary of the evidence on paying community health workers.

### The private sector and PHC

In many LMICS, the private sector is an important source of PHC provision.^40^, ^41^ Policy makers frequently express a desire to work with the private sector. However, the term private sector covers a heterogeneous set of providers, ranging from faith-based non-profit facilities that are well integrated into national health systems, to professionally trained clinicians operating private practices, to informally or untrained providers providing unregulated, low quality, even dangerous care. As noted, the Commission contends that universal PHC must rely predominantly on pooled public funds—but this does not preclude engaging with the private sector. Indeed, public financing mixed with private provision is widely adopted in OECD countries, where private providers are the main mode of provision of primary care in half of countries.^42^

Integrating the private sector into PHC platforms requires mechanisms to channel public funds to the private sector through purchasing arrangements. This pathway requires effective regulation, contracting capacity, and a broader set of purchasing institutions, including accreditation. In low-income countries, where informal private providers predominate and the public sector has insufficient resources for administering effective strategic purchasing and oversight, the best policy option is likely to be to start by providing good quality and affordable services in the public sector, while progressively strengthening regulation and professional bodies and collaborating with the private sector to develop referral pathways, training and qualification, and integration of clinical data.^43^ As both the public and private sectors develop greater capacities, the private sector can contribute to broader PHC functions, including public health surveillance and other essential public health functions, civil registration processes, the health management information system, and outbreak management.

## Section 2: The landscape of financing and organisation of PHC

### Analysing PHC expenditure

This section sets the scene for the rest of the report by presenting data on several topics: how PHC is currently financed, how PHC providers are paid, and some of the features of how PHC is organised that are particularly relevant to financing arrangements. Key messages from this section are presented in panel 2.

Panel 2 **Primary health care (PHC) financing landscape–key messages**

• Despite the prominence of PHC in political commitments and policy statements, limited information is available on levels of, or trends in, financial resources for PHC. Different methods of calculating PHC spending are used, making it hard to compare expenditure data from different sources.
• Annual government spending on PHC is $3 per capita in low-income countries and $16 per capita in lower-middle-income countries, which falls far short of any commonly used benchmark of the minimum amount needed to provide a basic package of health services.
• Much of the variation across countries in estimated spending levels on PHC can be explained by national income level. However, there is also substantial variation in government spending on PHC among countries at similar income levels.
• Higher levels of spending on PHC are generally associated with higher levels of service coverage. However, at any given spending level, there is substantial variation in performance, indicating that there is a need to spend better, as well as to spend more, on PHC.
• Financing of, and spending on, PHC are fragmented. Governments typically invest in outpatient services, donor funding is used for prevention, and nearly half of private spending (most of which is out of pocket) is on medicines. Although external funds are an important source, particularly in low-income settings, that augment government and out-of-pocket expenditures, they can also cause fragmentation.
• Out-of-pocket spending on PHC remains unacceptably high, particularly in low-income countries, continuing to expose households to financial risk.
• The most common method for paying public providers for PHC in low-income to middle-income countries is input-based budgets. Capitation-based payment systems for PHC are rare in low-income to middle-income countries.
• Public providers have little autonomy over their spending, which limits their efficiency and responsiveness.

To prioritise PHC expenditure, it is first necessary to measure what is currently spent. Empirical spending data allow policy makers to track existing expenditure, show how funds are currently being used, and make a case for increased commitments. Without data, it is hard to steer health systems toward stated goals, including equity, and even harder to track progress.

#### The first challenge: defining PHC for financing analysis

Analysing the financing arrangements for PHC requires clarity about what is (and what is not) part of PHC. Measuring PHC spending is challenging for numerous reasons, beginning with the breadth of the definition of PHC. As noted in section 1, PHC can include functions outside the health sector. Yet for conceptual and practical reasons, the current standard framework for tracking all financial resources for health in a country restricts the boundaries of health spending to the health sector itself. This framework, the System of Health Accounts, does refer (eg, through memorandum items for health promotion with a multisectoral approach) to expenditures by other sectors that clearly contribute to health, such as water and sanitation, but data on spending in these areas are not collected systematically.

Even within the health sector, there is no clear consensus on how to operationalise the definition of PHC in a robust expenditure monitoring exercise. The System of Health Accounts does not include a designated category for reporting spending on PHC.^44^ Instead, it classifies health spending in various ways, including by type of provider and by type of health-care service. In practice, most countries' definitions of PHC cut across these two classifications. Furthermore, the level of detail possible in System of Health Accounts reporting does not allow for accurate tracking of expenditure through to PHC services; instead System of Health Accounts measures rely on proxies derived from reported aggregates. For example, some countries do not distinguish expenditure on general outpatient services from that on specialised outpatient services, so their PHC expenditure estimates include both. The System of Health Accounts also does not distinguish among different levels of hospital care; however, in many countries district hospitals are part of PHC, while tertiary care is provided at centralised or regional hospitals. When used to estimate PHC expenditure, such approaches can give an impression of relatively high levels and shares of PHC spending. Panel 3 describes three countries' different approaches to organising PHC, and how the differences are reflected in expenditure tracking.

Panel 3 **Three countries' approaches to defining primary health care (PHC)**

Despite the existence of a global expenditure classification system, countries vary considerably in their models and conceptualisation of PHC, including how PHC spending is measured. Three examples, Thailand, South Africa, and the UK, are presented to show the breadth of this variety.

**Thailand** *

In Thailand, PHC is defined differently in rural and urban areas. PHC in rural areas includes all services provided by the District Health Systems Network, which is comprised of public subdistrict health centres and district hospitals (each of which serves a catchment of approximately 50 000 people). The District Health Systems Network is the first entry point to PHC for all people living in rural areas and provides a comprehensive range of services throughout the life course, including health promotion, disease prevention, and primary care services. It also provides public health functions, such as disease surveillance and response, and home visits, and supports multisectoral action to address social determinants of health and empower citizens and communities. In urban settings, meanwhile, PHC is less well-developed and most of the population uses hospital outpatient care directly, without any gatekeeping. In these settings, PHC is provided by the public and private hospital-based outpatient departments that provide a comprehensive range of primary care services similar to the District Health Systems Network.

Based on these definitions, the Thailand National Health Account estimates PHC expenditure as the sum of general and dental outpatient curative and preventive care provided at subdistrict health centres, and at public and private hospitals.^45^ Between 2015 and 2019, total PHC spending was 38% to 40% of current health expenditure. About 60% of PHC spending was financed by three public-health insurance schemes. Household out-of-pocket payments represented between 4% and 7% of PHC spending, as the publicly-covered benefit package is comprehensive and medicines are fully subsidised. Per capita PHC spending increased from US$85·7 in 2015 to US$114·8 in 2019.

**South Africa**

In South Africa, a uniform budget structure for the country's health programming was designed to specifically designate subprogrammes for PHC. The classification system is standardised across provinces and districts. Overall, South Africa spent US$92·9 per capita on public sector PHC in 2019–20. The five main subprogrammes of PHC comprise 18·8% of provincial health expenditure. South Africa also has a large, separately designated, HIV and AIDS subprogramme that adds an additional 10·4%, bringing total PHC expenditure to 32·3% of public health expenditure, or 1·3% of gross domestic product. This budget subprogramme classification differs somewhat from the WHO and System of Health Accounts 2011 classification system: the 2016–17 National Health Accounts for South Africa showed PHC represented 28% of government health expenditure.

**UK**

The National Health Service (NHS) in the UK describes PHC as “the first point of contact in the health care system, acting as the ‘front door’ of the NHS”. PHC is most closely linked to doctors in general practice, but also covers dentists, opticians, and pharmacists. Other professionals, such as nurses or physiotherapists, might also be part of a PHC team. Most primary care providers in the UK operate as independent contractors for the NHS. A range of services are contracted and financed through various models such as the General Medical Services or Personal Medical Services, Alternative Provider Medical Services, and Primary Care Trust Medical Services. According to the 2019–20 Annual Report and Accounts of the Department of Health and Social Care, primary care accounted for £12·6 billion (8·6%) of the total gross government expenditure on health of £145·27 billion. Prescribing costs, tracked separately, amounted to an additional £8·5 billion.

Many countries' expenditure reporting systems are inadequate for tracking expenditure on PHC. Although both OECD and WHO use the System of Health Accounts 2011 framework to track total health spending, only OECD collects disaggregated health spending (based on both the health-care function and provider classifications) for its 34 high-income-country members. WHO began tracking health spending based on health-care functions in 2016, but does not report health spending classified by provider. In 2016, WHO reported PHC estimates for 93 countries, increasing to 98 countries in 2018 (the most recent data available). Furthermore, breakdowns of public and private spending are only available in the WHO database for 57 countries in 2016, and 61 in 2018. Both the availability and the reliability of expenditure data on PHC in LMICs remain inadequate because of underinvestment in resource tracking systems, as well as the complexity of defining and tracking expenditure on PHC.^6^

Finally, different international bodies and countries compile PHC expenditure using different definitions (panel 4). Not only is there no agreed-on approach to estimate spending on PHC, but there is also no single database that provides globally comparable data for analysing PHC expenditure.

Panel 4 **Working with Organisation for Economic Co-operation and Development (OECD) and WHO definitions of primary health-care (PHC) expenditure to construct an expenditure database on PHC**

To prepare our landscape analysis of PHC expenditure across countries, the Commission needed to combine data from various sources, considering different definitions and assumptions. We focused on two key sources: OECD^46^ and WHO.^47^

The OECD definition of PHC begins with expenditure on basic health care services derived from the health care function (HC) classification (namely, the sum of spending on general outpatient curative care (HC131), outpatient dental care (HC132), home-based curative care (HC14), and preventive care (HC61-HC64).^48^ An extended option also includes spending on pharmaceuticals. PHC expenditure is defined as expenditure on these services, limited to those delivered by ambulatory care providers derived from the health provider classification. WHO's definition of PHC uses only the HC classification. It starts with the same basic health care services in OECD's definition, and adds four additional components:

• Curative outpatient care not elsewhere classified (HC13).
• Outpatient and home-based long-term health care (HC33 and HC34).
• 80% of medical goods provided outside health care services (HC5). Because HC5 also includes inpatient-related medicines purchased outside health care facilities, the 80% share is an assumption aimed to capture just the PHC element.^44^
• 80% of health system administration and governance expenditure (HC7); this is included to represent the share of administrative expenditures related to policy and implementation costs for population-based public health interventions.

Because the WHO definition includes spending on hospital-based general outpatient care, pharmaceuticals, and administrative costs, the OECD definition is a subset of the WHO definition. Therefore expenditure levels estimated using the OECD definition will always be lower.

**The Commission's definition**

The Commission used a definition of PHC expenditure based on the WHO definition to compile the data presented below. However, we excluded the administration and governance expenditures. This brings our definition closer to that used by the OECD and limits additional arbitrary assumptions. We are not suggesting that administration and governance are not important for PHC; rather, we believe that these inputs are better captured outside the direct measurement of PHC spending. The inclusion of administration costs disproportionately biases estimates of PHC in low-income countries upwards. Even with this restriction on the definition of PHC spending, our estimates have been calculated using a broad definition of PHC that does include some hospital care. Therefore, our estimates should be seen as an upper bound estimate.

Details on how we combined data from the WHO and OECD databases to increase the number of countries we covered are presented in the appendix (p 5).

Definitions matter. They signal what is prioritised and valued, and they shape norms regarding how services should be organised. They also influence how data are collected and presented. There is a trade-off between using a simple global definition and accounting for country-specific definitions to permit more accurate reporting. In these early days of PHC expenditure reporting, what is most crucial is for each country to choose a way of operationalising PHC expenditure estimates so it can track its progress. Eventually, however, a consistent definition across countries will be needed to allow for cross-country comparisons and global monitoring of expenditure on PHC.

In generating our estimates of current levels of PHC expenditure, this Commission was constrained by both the levels of current reporting and the definitions used by the organisations that compile data.

#### The Commission's method of calculating PHC expenditure

The Commission's approach to measuring expenditure on PHC is presented in panel 4, along with the thinking behind it. For the purposes of tracking expenditures on PHC in the future, this Commission favours using an operational definition based on service delivery platforms for PHC: population-based public health services, community health services, health centres, and first-level hospitals. This is because financing arrangements typically channel resources to providers and platforms, rather than to interventions or services. We also take the normative position that PHC should not be delivered through higher-level hospitals, because improving financing arrangements should focus on driving resources to and supporting use at the appropriate level of care, which brings services as close as possible to people and delivers them at the lowest cost. To estimate PHC expenditure using a platform-based approach requires data that cross-classify between health care function and provider category. However, because currently available data from WHO are only reported by health-care function, we are limited to estimating PHC expenditure using a service-based (rather than a platform-based) approach. We also made some modifications to the PHC expenditure reported by the WHO for the purposes of our analysis (panel 4).

WHO reported data on total PHC spending on 98 countries for 2018 and government spending on PHC on 61 of these. We observed that the WHO database did not provide data on government spending on PHC for all OECD countries. In order to compare spending levels in high income countries with those in LMICs, we constructed PHC expenditure for OECD countries from data on expenditure by financing scheme, using expenditure by government and compulsory schemes to proxy for government spending on PHC. We used exchange rate data from the Global Health Expenditure Database to convert the raw data from local currency. With the addition of reconstructed government spending on PHC per capita from the OECD database following the WHO definition, the total number of countries providing data on government spending on PHC increased to 90 countries.

#### Levels of financing for PHC

Despite data limitations, our analyses have identified some notable patterns in the levels and sources of PHC spending across countries. Table 1 presents overall health expenditure data by country income group for 2018. The table shows that total expenditure on PHC in low-income countries is $24 per capita and in lower-middle-income countries it is $52 per capita. Government spending on PHC is even more meagre, at $3 in low-income countries and $16 in lower-middle-income countries, which falls short of the WHO estimate of the per capita recurrent cost for PHC of $65 in low income countries and $59 in lower-middle income countries^10^ (section 3). Although the share of government health spending allocated to PHC is similar in LMICs and high-income countries, government spending on PHC as a share of total PHC spending is lower in low-income countries than in high-income countries and the same is true of the share of government PHC spending in relation to GDP.

**Table 1:** Summary of health expenditure in 2018^40^, ^41^

	**Level of spending per capita (US$)**						**Share of spending (%)**					
	(Total) Current health spending	Domestic general government expenditure on health	Total PHC spending	Domestic general government spending on PHC	Out-of-pocket spending on PHC	External spending on PHC	PHC spending as a share of current health spending	Domestic general government spending on PHC as a share of total PHC spending	Domestic general government spending on PHC as a share of GDP	Domestic general government spending on PHC as a share of domestic general government expenditure on health	Out-of-pocket spending on PHC as a share of total PHC spending	External spending on PHC as a share of total PHC spending
Low-income group	40 (n=16)	8 (n=16)	24 (n=16)	3 (n=14)	12 (n=14)	8 (n=14)	59% (n=16)	13% (n=14)	<1% (n=14)	33% (n=14)	44% (n=14)	35% (n=14)
Lower-middle-income group	104 (n=25)	44 (n=25)	52 (n=25)	16 (n=24)	23 (n=24)	8 (n=24)	52% (n=25)	29% (n=24)	<1% (n=24)	36% (n=24)	49% (n=24)	14% (n=24)
Upper-middle-income group	416 (n=20)	242 (n=20)	169 (n=20)	73 (n=19)	65 (n=19)	6 (n=19)	42% (n=20)	45% (n=19)	1% (n=19)	34% (n=19)	39% (n=19)	5% (n=19)
High-income group	3310 (n=34)	2355 (n=34)	1312 (n=34)	840 (n=33)	318 (n=33)	0 (n=33)	42% (n=34)	59% (n=33)	2% (n=33)	36% (n=33)	28% (n=33)	<1% (n=33)
Total	1306 (n=95)	907 (n=95)	523 (n=95)	328 (n=90)	139 (n=90)	5 (n=90)	48% (n=95)	41% (n=90)	1% (n=90)	35% (n=90)	39% (n=90)	10% (n=90)

GDP=gross domestic product. PHC=primary health care. OECD=Organisation for Economic Co-operation and Development. For total PHC spending, WHO provides data for 98 countries and OECD provides data on high income countries that overlap with WHO data but with an additional five countries which brings a total of combined data for 103 countries.. However, we excluded eight countries due to the inconsistency in how they reported health spending by functions (France, Greece, Ireland, Italy, Mexico, Portugal, UK, and USA). The total number of countries for which we present data is therefore 95 countries. For government spending on PHC, WHO provides data for 61 countries and OECD provides data for an additional 36 high income countries that brings a total of combined data for 97 countries. However, we excluded seven countries due to the inconsistency in how they reported health spending by functions (France, Greece, Ireland, Italy, Portugal, UK, and USA). This gives a total number of 90 countries for this indicator. For five countries, the Global Health Expenditure Database reports total PHC spending data but no disaggregation by financial source (ie government, external, and private [Bosnia and Herzegovina, Democratic Republic of Congo, Ethiopia, Ghana, and Uruguay]). Only five high-income countries report external funding for PHC (Barbados, Mauritius, Seychelles, St Kitts and Nevis, and Trinidad and Tobago). For other high-income countries external spending is negligible or zero. Averages are unweighted means across countries. To calculate the average of ratios, we calculated the ratio for each country and took the average for each income group. The sum of government, external, and out-of-pocket spending will not be equal to the total spending due to the omission of other types of private spending, such as voluntary private insurance. The gap is bigger in high-income countries because private insurance is more common in high-income countries. Although out-of-pocket spending on PHC is available in the OECD database, WHO only reports domestic private spending on PHC. To estimate the out-of-pocket spending for PHC in low-income to middle-income countries, we took the ratio of total out-of-pocket spending on health to total domestic private spending on health and multiplied it by private spending for PHC for each country.

A descriptive analysis of PHC expenditure patterns is presented in the appendix (p 9). There is a strong correlation between income level and health expenditure on PHC, yet there is also substantial variation within any given economic level in how much governments spend on PHC. For example, spending in low income countries ranged from $8 to $46 and in lower middle-income countries, spending ranged from $11 to $120 per capita (appendix p 9). The share of PHC in current health expenditure (which includes expenditure from public, private, and external sources) decreases as countries' income levels increase—again, substantial variation exists at every income level. For example, the share in low income countries ranged from 29% to 86% (appendix p 10). Priority given to PHC within government health spending is similar on average across income groups, although there are variations in commitment at any given income level and this is more pronounced in low-income and lower-middle-income countries. However, the government share of total PHC spending was lowest in low-income countries, where both external sources and private spending (in LMICs, predominantly out-of-pocket spending) has a substantial role in financing PHC. Indeed only 13% of PHC financing in low-income countries is public.

**Figure 1:**
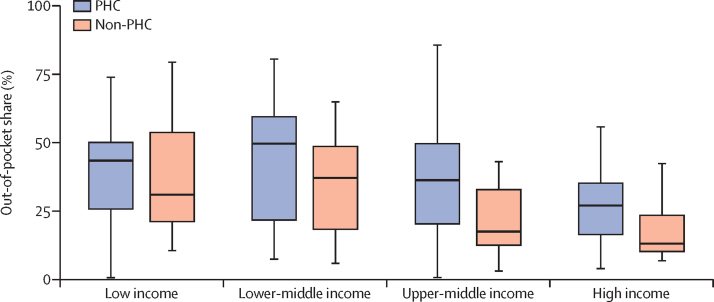
Out-of-pocket household spending as a share of total spending for PHC and non-PHC in 2018, by country income Out-of-pocket share of PHC is calculated as out-of-pocket spending on PHC as a share of total PHC spending. Out of pocket share of non-PHC is calculated as out-of-pocket spending on non-PHC as a share of total non-PHC spending. The box represents IQR; the ends of the whiskers represent the minimum and maximum value; the bold horizontal line represents the median. PHC=primary health care.

Financing for PHC in low-income and lower-middle-income countries is dominated by relatively unregulated private expenditure, most of which is out of pocket (figure 1). Only in high-income countries does the average share of out-of-pocket spending on PHC fall below 30% (although even in this group of countries, there is a long tail of countries with substantial out-of-pocket spending for PHC, suggesting that the issue is one of policy, not availability of resources). Figure 1 also compares the out-of-pocket share of PHC spending with the out-of-pocket share of non-PHC spending. At all country income levels, households are more exposed to out-of-pocket spending for PHC than for other health spending. This finding suggests that pooling arrangements provide less coverage for PHC and that households are more likely to be exposed to catastrophic financial consequences of paying for PHC—this underlines the importance of including PHC in benefit packages. The high level of out-of-pocket spending for PHC is particularly worrisome in LMICs, where the majority of people die from preventable causes that could be managed at the PHC level, and where poor people might be more likely to forego PHC than advanced specialist care. We contend that the lack of pooling for PHC runs counter to a progressive universalism approach to PHC, and exacerbates inequities. Finally, it is important to note that these figures do not account for people who are not able to access PHC at all; this is a general limitation of any equity analysis based on incurred expenditure.

**Figure 2:**
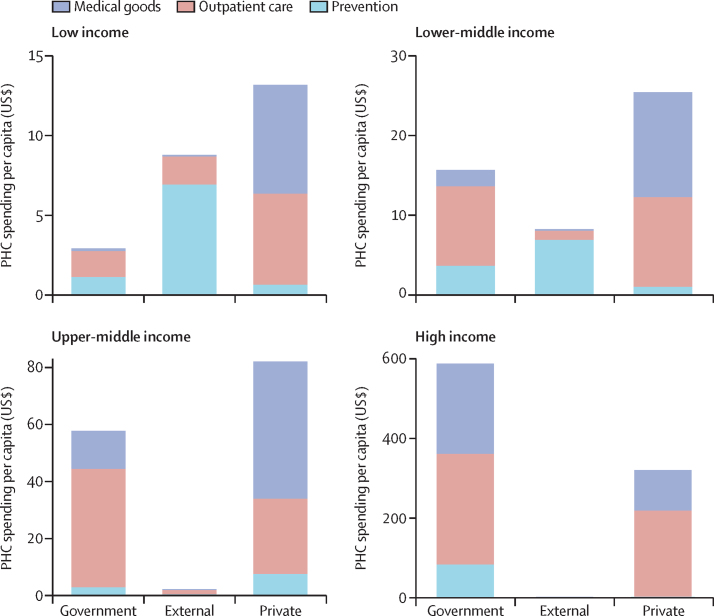
Components of PHC spending by financial source in low-income to high-income countries in 2018 Figures exclude dental care and home care. Medical goods only include medicines and other medical goods purchased outside of outpatient facilities. Private spending includes individuals paying out of pocket and other domestic private sources, such as private insurance (voluntary and compulsory). External spending includes the use of grants, concessional loans, and aid in kind from outside the country. PHC=primary health care

It is also notable that about half of private spending on PHC (most of which is out of pocket in LMICs) is for medical goods purchased outside health services (figure 2). Much of this is likely to be for medicines; for example, *The Lancet* Commission on Essential Medicines for Universal Health Coverage^49^ reported that more than 62% of pharmaceutical expenditure in LMICs was from private sources, which is likely to be mostly out-of-pocket spending considering the low levels of prepaid and pooled resources.

In LMICs, the largest share of government expenditure is for outpatient care. Within this spending category, government health spending is highly skewed towards health-worker salaries. WHO's analysis of the Global Health Expenditure Database indicates that for 136 countries, 57% of public spending on health is allocated to wages.^50^ For PHC, in which few other inputs are used, salaries are likely to be an even higher share. This balance of spending likely represents a source of inefficiency if, after paying the wage bill, insufficient funds are left to purchase other inputs required for health workers to work effectively.

In low-income and lower-middle income countries, where external funds are a significant contributor to PHC expenditure, donor funds are predominantly spent on prevention. Figure 2 suggests significant fragmentation of PHC expenditure across financing sources.

**Figure 3:**
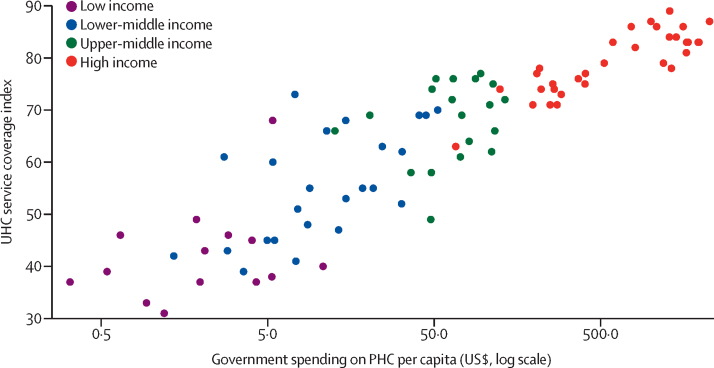
Government spending on PHC versus UHC index, 2018 PHC=primary health care. UHC=universal health coverage.

Does the level of spending matter? This Commission examined whether there is a relationship between overall government spending on PHC and coverage of key services (figure 3). We used the universal health coverage (UHC) service coverage index^51^ as a proxy for PHC service coverage—of the 14 variables indexed, 11 relate to core PHC services (in addition, the index includes hospital bed density, health-worker density, and capacity to implement the International Health Regulations). Two key findings emerge from the analysis. First, higher government spending on PHC is strongly associated with better service coverage. This relationship remains strong after adjustment for GDP per capita (result not shown), which figure 3 suggests could be a potential confounder. Second, for any given level of government spending, particularly below $50 per capita, there is substantial variation in performance on the service coverage index. For example, countries that spent between $50 and $55 have a range of UHC index from 42 to 68. Whether there is a causal relationship underlying the association between government spending on PHC and service coverage is hard to show. However, the data are consistent with the notion that countries should not only spend more but also spend better to achieve improved coverage of core PHC services.

#### The role of donors in financing PHC

Low-income countries depend substantially on external sources to pay for PHC (appendix p 11). External funding typically focuses on prevention and treatment of single diseases, which can contribute to fragmentation in financing arrangements and PHC delivery—this is especially evident when externally funded programmes are not part of government planning and budgeting processes. Community health worker programmes, which often form the backbone of PHC delivery, are also highly dependent on donor funding: an estimated 60% of funding for community health worker programmes in sub-Saharan Africa comes from external sources, much of which is funding for vertical, disease-specific programmes.^52^ The fragmentation of PHC financing related to reliance on donor funding is due to requirements for tracking and reporting separately on donor funded activities. It might also be a source of inefficiency due to the so-called start-stop nature of donor funding (as compared with government budgeting).^53^

#### Limitations of our analyses

There are limitations to these analyses. First, use of the WHO's broad PHC expenditure definition has the effect of biasing the estimates of PHC spending upwards (because of the inclusion of outpatient services provided in hospitals). Any definition that uses a narrower scope would produce lower estimates of PHC expenditure. For example, in 2019, the OECD reported that spending on primary care within ambulatory settings represented just 12% of health expenditure; this increases to 17% when PHC services delivered in hospital settings are included, and to 34% by including retail pharmaceuticals.^48^

Second, data are only available for 95 out of 192 countries and for a single point in time. The spending estimates for some income groups are affected by the small number of countries in each group. Most importantly for our purposes, the absence of consistent and comparable data over time means that it is not possible to easily identify which countries are increasing or sustaining their commitments to PHC. Therefore, although these results are informative, throughout the report we also include more qualitative assessments of countries' progression towards adequate financing for PHC.

Even with our limited dataset, we argue that LMICs need to spend more on PHC to provide equitable and universal access PHC that is people centred. Further, the Commission contends that adopting a definition of PHC that is consistent with the vision of providing health care at the lowest possible level, and then supporting countries to collect and report data disaggregated in this way, are essential steps toward improving the quality of national data on PHC expenditure. Data, after all, are an essential part of the strategy for monitoring and actively protecting resources for PHC going forward, and for enabling countries to show their progress in increasing their commitment to PHC.^52^

### Organisation and provider payment for PHC

**Figure 4:**
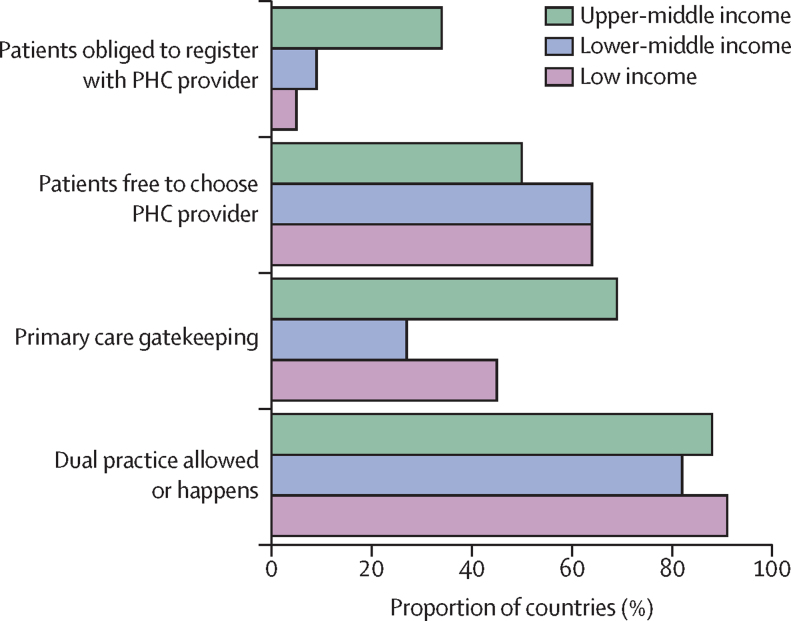
Organisation and governance of public PHC providers Data indicate the proportion of countries with each organisational and governance arrangement in place. Data are disaggregated by country income group. PHC=primary health care.

**Figure 5:**
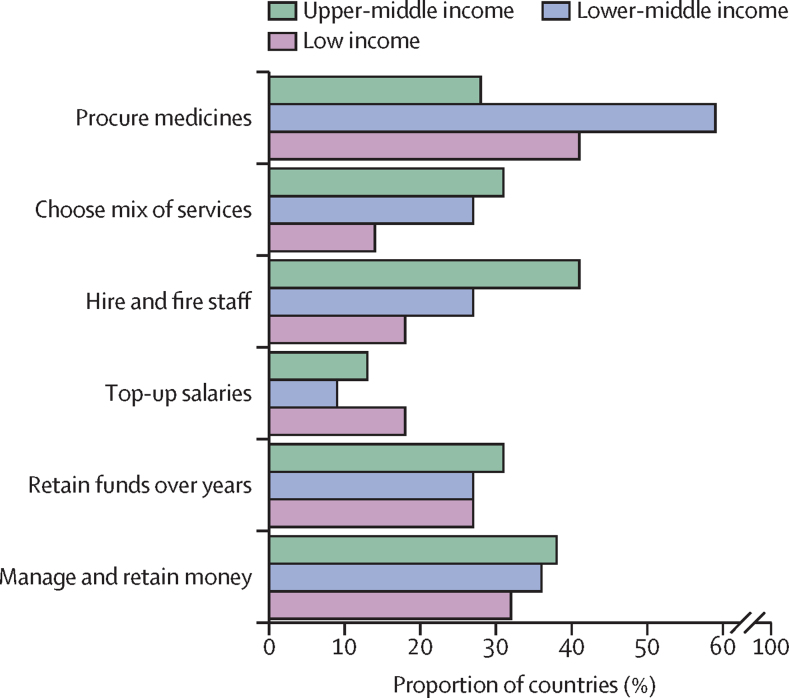
Degree of autonomy for public PHC providers Data indicate the proportion of countries in which public PHC providers have autonomy in how they function. Data are disaggregated by country income group. PHC=primary health care.

**Figure 6:**
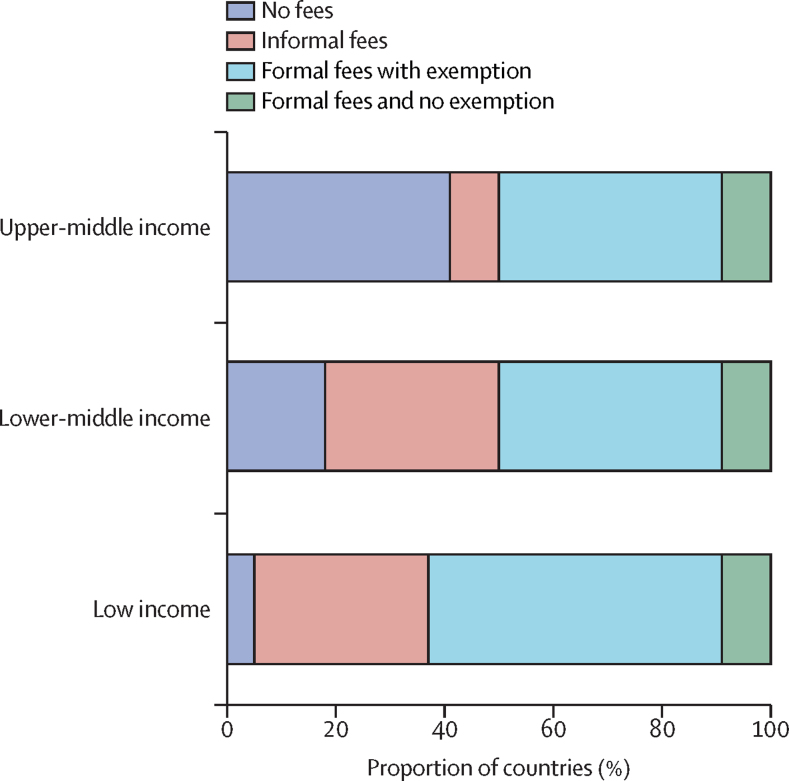
Formal and informal user fees at public PHC providers Data indicate the user fee policy in place in public PHC providers across surveyed countries (categories are mutually exclusive). Data are disaggregated by country income group. PHC=primary health care.

**Figure 7:**
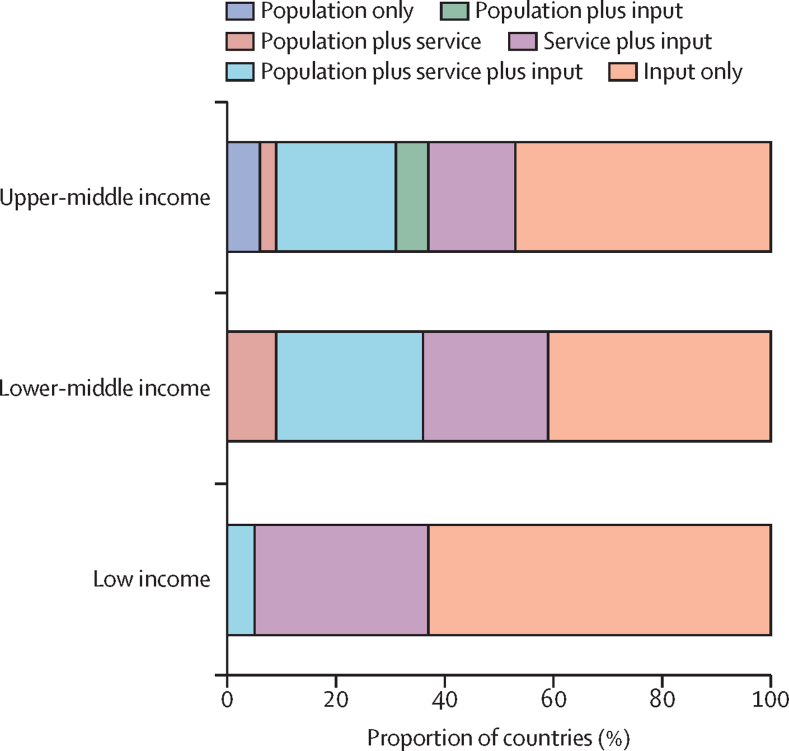
Provider payment mechanisms for public PHC providers Data indicate the type of payment system used in public PHC providers across surveyed countries (categories are mutually exclusive). Data are disaggregated by country income group. Population payment refers to capitation. Input-based payment refers to global budget, line item budget, and direct payment of salaries by government. Service-based payment refers to fee-for-service, case-based payment and pay-for-performance. PHC=primary health care.

**Figure 8:**
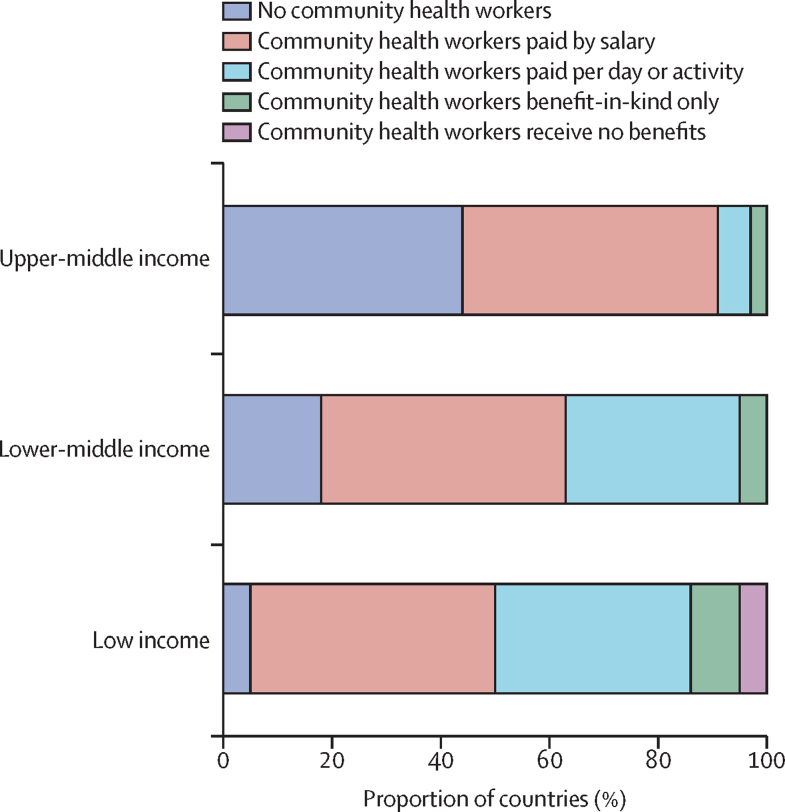
Paying community health workers Data indicate how community health workers delivering services on behalf of the government are remunerated in surveyed countries (categories are mutually exclusive). Data are disaggregated by country income group.

Countries differ widely in terms of how PHC is structured and organised, including whether PHC includes community health workers and how they link to health facilities. Yet, although the availability of data on PHC expenditure in LMICs is improving, there is still little systematically collected cross-country data on the financial arrangements of LMIC health systems and provider payment structures. This contrasts with the data collected from OECD countries, for which the Health System Characteristics Survey provides comparable data on how countries finance, deliver, allocate resources to, and govern their health systems.^42^ To address this important data gap, in this Commission, we did our own cross-sectional survey in LMICs with the aim of collecting data on the key financing-related features of how PHC is organised, and on how PHC providers are paid. The questionnaire was sent in a personal email to health financing experts identified through the networks of the London School of Hygiene & Tropical Medicine, the World Bank, Results for Development, and WHO. The survey could be completed in two ways: either through self-completion or through a videoconference interview (for methods see appendix p 13). The survey was sent to 107 LMICs (one expert from each country), of which there were 75 responses: 22 from low-income countries, 22 from lower middle-income countries, and 31 from upper middle-income countries. Figure 4, Figure 5, Figure 6, Figure 7, Figure 8 present key findings from this survey.

#### How PHC delivery is organised

Figure 4 presents results on indicators of how PHC services are organised that have implications for provider payment. The requirement for people to register with a public PHC provider, necessary for population-based forms of provider payment such as capitation, is uncommon in low-income and lower-middle-income countries, but more common in upper-middle-income countries. People are not restricted in their choice of public PHC providers in two-thirds of low-income and lower-middle-income countries. More restrictions on choice of provider (often in relation to the requirement to register with a provider) in upper-middle-income countries suggest that these countries seek to encourage greater consistency in use of PHC providers. Gatekeeping (in which individuals are required, or have strong financial incentives, to be referred by primary care providers to access higher-level services) is used in less than half of low-income countries. It is present in nearly three-quarters of upper-middle-income countries, although in practice these systems might frequently be circumvented. Dual practice (when a doctor works in both public and private sector practices) is common in all LMIC settings.

The extent of provider autonomy links closely with provider payment arrangements, because changing providers' incentives without enabling them to make decisions about how to use their resources constrains the potential for improvements in responsiveness and efficiency. Figure 5 shows that public sector PHC providers generally lack autonomy, with fewer than half of the countries included in the survey granting providers autonomy in any domain. More specifically, public PHC providers in fewer than 40% of LMICs have autonomy to manage and retain income, nor do they typically have the autonomy to top-up the salaries of their staff to reward performance. Provider autonomy to hire and fire staff, for example, tends to increase as country income level increases, with PHC providers in more than 40% of upper-middle-income countries able to make such decisions. There is a similar pattern across country income groups when it comes to autonomy to choose the mix of services to provide. Regarding procurement of medicines, however, autonomy is greatest in lower-middle-income countries—this might reflect chronic shortages of inputs.

#### How PHC providers are paid by different funding sources

This Commission's survey confirms that user fees (whether formal or informal) are a common source of payment for PHC in low-income and lower-middle-income countries, although many countries have exemptions in place for specific population groups or types of services. Figure 6 shows that the prevalence of user fees as a funding source in the public sector decreases as countries get richer: about 95% of low-income countries report that there is some form of user fee, whereas fewer than 60% of upper-middle-income countries have them. Informal user fees remain a problem in both low-income and lower-middle-income countries; upper-middle-income countries do considerably better in this regard.

The survey also provided insights into how PHC providers are paid from pooled funds (most commonly government and social health insurance payers). Figure 7 shows that these two types of payer rely fully on input-based budgets in more than 60% of low-income countries, and on input-based budgets in combination with fee-for-service in a further 30% of low-income countries; the relevance of the type of budgeting used for PHC is discussed in detail in section 4. Service-based payment, which includes fee-for-service, case-based payment or pay-for-performance, are also commonly used by government and social health insurance payers, most notably in lower-middle-income countries where it is present in almost 60% of PHC systems. Population-based, or capitation, payment systems are rarely used in low-income countries. At higher income levels, there is wider use of blended payment methods that combine different payment mechanisms and thus open up the possibility of a greater role for capitation. Capitation payment, alone or in combination with other payment methods, is used in almost 40% of middle-income countries. Our survey also found that in countries where capitation is used, around two-thirds of PHC systems adjust the payment amounts to reflect variations in health needs (data not shown).

These findings from this Commission's survey support other evidence about the role of user payments in low- and lower-middle-income countries, and show that countries at higher levels of income are making greater use of capitation or blended payment as part of the move towards paying for people-centred PHC. These new data also provide a baseline for future efforts to track developments in PHC financing in LMICs.

#### Payment of community health workers

The Commission's provider payment survey also collected data on how community health workers are paid. Figure 8 shows that more than 80% of lower-middle-income countries and more than 90% of low-income countries reported having some form of community health workers, compared with just over half of upper-middle-income countries. The most common form of payment for community health workers was by salary, although payment per day or per activity was also common in low-income and lower-middle-income countries. A small minority of countries have their community health workers paid in kind only**,** such as gifts or training; no payment for community health workers was reported only in a small minority (less than 5%) of low-income countries. Evidence on different ways of paying community health workers is presented in section 5.

### New challenges for financing PHC

PHC needs to constantly respond and adapt to new challenges created by changes in the environment, population health needs, and our expanding understanding of the importance of pursuing equity. Improving financing arrangements is one key way to influence adaptations of PHC. The growing burden of non-communicable diseases, the inevitable emergence of new pandemics, rapidly changing digital technologies for delivery and administration, and the growth of the private sector will all force the delivery of PHC—and its financing arrangements—to change as well. Responding to changing conditions also generates many opportunities for policy makers to influence PHC and improve its capacity to provide people-centred care that better supports social goals, such as UHC and equity, on the national and global scales. Financing should, at a minimum, not impede needed changes in service delivery; at best, financing arrangements can serve as a transformational force. This section describes several challenges for delivering PHC and the promising approaches to organising PHC financing that are elaborated further throughout the rest of the report.

#### Financing to facilitate service integration

Coordination and integration of services is at the heart of people-centred care. PHC has been described as the coordination mechanism that links primary care, community care, specialised care, wider public health interventions, and long-term care services.^16^ However, efforts to improve coordination and integration often face challenges at the interfaces of services and specialities; as noted, in LMICs, many challenges have arisen from the absence of integration between PHC and donor-financed vertical programmes, such as those focused on maternal health or HIV and AIDS. Improving coordination among different PHC services is as important as coordinating PHC with higher levels of the health system.

Problematic financing arrangements are frequently at the core of these challenges. Different financing mechanisms for different sources of funds, the means of allocation and management of flows of funding, and complicated payment mechanisms all act as major barriers to the implementation of more integrated approaches to service delivery.^54^ As noted in the Commission's case study on Chile, for example, the absence of strategic coordination between the capitation payment for PHC and the application of diagnosis-related groups for hospital care made clinical coordination and integration of care difficult.^55^ Where PHC is provided by an unorganised private sector and paid for by out-of-pocket payments, care can be fragmented and create excessive financial burdens. In this situation, for example, every provider might require the same diagnostic tests to be repeated and there can be excess prescription of branded medicines.^41^, ^56^ Donor funding can be a further impediment to integration. How health financing arrangements can support integration is explored in section 5.

#### Digital innovations in health financing

One major development in PHC, as indeed in all sectors, has been the rapid development of new digital technologies. In this Commission, we reviewed the published and grey literature regarding two questions: where have digital technologies facilitated financing of health and PHC? And how have financing arrangements been adapted to facilitate the digital delivery of PHC (appendix p 17)? After the literature review, a roundtable discussion took place among 13 digital and health financing experts (academics, practitioners, and donors) and the Commissioners captured recent and unpublished experiences and insights (for methods see appendix p 35). Four main messages emerged from this process. First, very little robust evidence exists on the impact of digital technologies on health financing objectives, including financing for PHC. Rigorous research in this area is urgently needed. Second, digital technologies offer great promise to improve the efficiency of resource mobilisation and purchasing arrangements; however, caution is warranted. There are risks, such as fragmentation if it leads to multiple small funding pools or parallel electronic patient information systems. Another risk is misalignment with UHC equity principles; for example, for the moment, digital access is much higher in cities, although this is changing rapidly. Third, to date, digital technologies related to financing have mostly been applied to health-care purchasing. For example, in the Philippines, digitised claims analysis using artificial intelligence is being used to detect fraud;^57^ in India, provider payment has been facilitated through an online system for data reporting and online pay-for-performance of Accredited Social Health Activists.^58^ Some applications to revenue mobilisation and pooling have also been documented: in Ghana, mobile phone apps have simplified payment of health insurance contributions and enrolment reminders.^59^ Fourth, little has been documented about any actual adjustments to financing arrangements to facilitate online and digital delivery of PHC, beyond measures to include remote consultations in insurance benefit packages. However, this area is developing rapidly (for example, the introduction of Babyl, an artificial intelligence-supported digital health service provider, **i**n Rwanda)^60^ and often without formal assessment or evaluation. Financing arrangements must be adapted if digital solutions are to be included in pooled funding arrangements, and provider payment levels need to be adjusted to cover additional activities required to integrate new technology enabled activities.

Most recently, the public health and social measures put in place to address the COVID-19 pandemic, such as lockdowns and quarantines, accelerated the development and use of various forms of digital health-care delivery, including remote consultations and home-based monitoring. Financing arrangements were quickly adapted to enable these changes, such as extending benefit packages to include remote consultation. PHC needs to continue to embrace new technologies that support coordination, interoperability, and regulation; and financing arrangements should continue to adapt to accommodate these. It is also important to avoid a situation where multiple solutions act independently and foment fragmentation. The key will be to allow flexible financing approaches to support these developments, while ensuring that they are thoughtfully integrated into the wider health system to induce efficiency gains without sacrificing equity. This is discussed throughout the Commission.

#### Paying for medicines for PHC

The data presented above show the importance of expenditure on medicines—which is sometimes as high as 40–50% of all expenditure and is often paid for out-of-pocket through private pharmacies—in PHC spending in many LMICs (figure 2). However, medicines are often out of stock in public health facilities or not included in provider payments. Patients then pay out of pocket for medicines purchased in private pharmacies, representing a substantial barrier to the use of medically appropriate and high quality medicines. This is particularly apparent for non-communicable diseases.^61^ Such out-of-pocket expenditure leads to inefficient use of medicines and financial burdens for patients.

Effective financing arrangements for medicines for PHC should have two key outcomes: to provide financial protection against the costs of medicines, and to encourage efficient use of resources. Financing arrangements for paying for medicines should focus on four goals. (1) Including essential medicines in the PHC package that is covered by pooling arrangements, including those needed for management of chronic conditions. (2) Ensuring that sufficient funds are mobilised to supply these medicines and deploying all available policy tools to procure medicines efficiently (including essential medicines lists, bulk procurement, and market shaping to secure favourable prices). (3) Encouraging efficient use of medicines through appropriate incentives to providers, dispensers, and patients. (4) Enabling new ways for people to access medicines that allow them greater control and easier access, for example, through self-service vending machines,^62^ e-prescription and e-pharmacy.

#### Conclusion

In section 2, we provided an overview of how PHC is financed and how PHC providers are paid. Although the findings are instructive, it is clear that much of the global expenditure data on PHC are problematic. A consensus on a consistent operational definition of PHC would enable better expenditure analyses—it would also help to prioritise PHC, promoting the normative message that PHC should not be provided at higher level hospitals, and enable countries to better track their progress. Generating agreement on a definition requires taking a position on the trade-offs between, on the one hand, allowing flexible definitions that fully capture local particulars, and, on the other hand, having a consistent definition that enables global monitoring and comparisons.

Even if the data presented in this Commission significantly overestimate PHC spending, they still show that the levels of government spending on PHC in low-income and lower-middle-income countries are far from sufficient, and that a considerable share of current financing comes from out-of-pocket, not pooled, resources. Furthermore, the relationship between PHC spending and service coverage, as measured using the UHC service coverage index, shows that existing money could be spent more effectively. These findings underpin the Commission's overarching message: that countries need to spend more and spend better on PHC. Better spending would mean replacing out-of-pocket spending on PHC with pooled public sources (see sections 3 and 4); ensuring that the resources intended for PHC reach the frontline providers; reducing fragmentation of funding; and expanding provider autonomy by improving how providers are paid to better incentivise people-centred PHC (see section 5).

## Section 3: Mobilising and pooling resources to finance health care

This section addresses how to take a people-centred approach to raising more prepaid and pooled funding for PHC by mobilising more funding for health overall. The issue of how resources are allocated to PHC from the overall health budget is addressed in the next section, although these are closely intertwined in practice. This section includes: an overview of the challenges faced in mobilising and pooling financing for health, a vision for increasing resources for health, and some possible strategies to pursue this vision. It is important to note throughout that stakeholders beyond the health sector have the greatest responsibility for raising additional resources for health. The ministry of finance is key because it both has responsibility for raising government revenue and wields significant power in budget-setting negotiations with spending ministries, such as the ministry of health. Key messages from this section are presented in panel 5.

Panel 5 **Mobilising and pooling resources for primary health care (PHC)–key messages**

• Government expenditure on health falls short of what is needed for UHC, which limits the overall ‘pie’ available for the PHC share and forces patients to continue to pay out-of-pocket, posing a persistent barrier to access. PHC should be free at the point of use because even small payments can deter use. This requires progressive removal of user fees and increased public funding. Increasing funding will be challenging in the near-future because of the economic impact of the COVID-19 pandemic, which will constrain government budgets.
• Generating additional pooled resources is a challenge: fiscal capacity remains limited by macroeconomic conditions and inefficient revenue collection; however, additional resources will have to come mainly from taxes (general or earmarked); expansion of social health insurance, a strategy being pursued in many low-income to middle-income countries, is constrained by the small size of the formal labour force in many countries; thus donors continue to have an important role to play in low-income settings.
• Increasing tax revenue is both a technical issue (how to increase tax capacity and how to broaden the tax base) and a political issue (due to acceptability and compliance): the latter requires skilful engagement in budget politics.
• Better spending of available resources is key, although the potential to generate efficiency savings in the health sector is limited within existing institutional arrangements; it also takes time (and often investment) to achieve these savings.
• Better pooling arrangements are also needed to reduce fragmentation, secure equitable cross-subsidies and efficient integration between levels of care. Where actual pooling is not possible, intermediate pooling supported by, for example, harmonisation of financing arrangements across pools, or virtual pools supported by digital technologies, can provide intermediate solutions.

The Commission has not taken a position on a target spending level for health. That topic has been considered by various bodies that have each proposed their own benchmarks.^63^, ^64^, ^65^, ^66^, ^67^ Various governments and donors have committed to these benchmarks, although few have ever actually met the targeted spending level. Regardless of which benchmark is considered, LMIC governments and donors do not yet spend enough on health.^33^ These benchmarks might be useful for advocacy purposes, and to give a global indication of how much is needed overall. However, they do not recognise that a given level of spending could be necessary but not sufficient to achieve desired outcomes. The benchmarks also disregard the substantial variation in performance across countries with similar levels of government budget allocation to health.^68^ Whatever the benchmark, estimates of resources required should be based on per-capita needs rather than simply funding facilities or infrastructure.

### Challenges in mobilisation and pooling of funding for health

The existing mechanisms for mobilising and pooling health funds in LMICs have multiple weaknesses that arise from their fragmented and insufficient revenue sources and the inherent challenges of pooling and managing resources. There are several specific problems.

The first is limited tax revenues. Public spending, including on health budgets, is often constrained by the general macroeconomic conditions and underlying weaknesses in national systems of taxation that limit the revenue base for public spending (as shown by the low mean tax–GDP ratio in low-income countries of 12%, as compared with nearly 30% in high-income countries).^69^

The second is social health insurance contributions. Social health insurance contributions represent an important source of health funding in several high-income countries.^33^ However, the formal labour force in many LMICs is insufficient to support expanding social health insurance contributions; in low-income countries, for example, only 10·2% of the population works in the formal labour sector.^70^ The COVID-19 pandemic has increased unemployment in the formal sector, with ever more workers shifting to the informal sector where it is more difficult to collect contributions.^70^ Furthermore even when social health insurance contributions are collected, translating them into additional revenue for health is made complicated by the issue of fungibility (interchangeability) within government budgets.

Third, a larger government budget does not necessarily lead to greater allocation to health. Even when governments do manage to expand the total envelope of available funds, either through tax revenues, social health insurance contributions, or donor financing, this does not necessarily lead to greater allocations to health.^33^ Failure to prioritise funding for health in total government spending might be due to technical issues (such as insufficient capacity within ministries of health to present strong investment cases for health to ministries of finance).^71^ It might also arise from public finance management structures that are not aligned with health budgeting needs or as a consequence of various political economy factors such as power imbalances and low levels of accountability between health system users and political leaders, and lobbies promoting other public expenditure priorities (see section 6).

The fourth problem is inadequate, declining, and fragmented donor funding. Donor funding for health, although valuable and, for the foreseeable future, essential for low-income countries, has been falling since 2015_·_^33^ The economic effects of the COVID-19 pandemic are likely to also negatively affect donors' budgets. For example, in 2021, the UK Government reduced its international aid spending target from 0·7% of gross national income to 0·5%. Furthermore, donor development funding can be unpredictable even in good economic times as it is based on donor preferences. The funding that is made available is typically largely targeted to specific disease programmes (in low-income countries, for example, an average of 65% of donor assistance is allocated to infectious and parasitic diseases).^33^, ^72^ Further, donor contributions tend to operate off-budget, undermining countries' ability to plan and manage these resources alongside domestic funding.^33^, ^72^ Nevertheless multilateral and bilateral assistance for selected low-income countries remains essential to at least 2030, and an improved global system of multilateral country support is required.

The fifth is excessive reliance on out-of-pocket spending. As alluded to previously, and as a result of the difficulty in raising pooled public funding, out-of-pocket payments by individuals are the dominant source of funding for health in low-income countries: they represent, on average, more than 40% of all health spending across all LMICs, and nearly 60% in the 32 low-income countries.^33^ Out-of-pocket payments can include formal user fees, informal fees levied by individual providers, travel costs, and payments for medicines and other supplies. Because payments made out of pocket are not pooled, they curtail cross-subsidisation between rich and poor, as well as between healthy and sick populations. The global health and economics communities have stated unequivocally that out-of-pocket payments need to be reduced (and user fees for PHC removed) and instead replaced with prepaid and pooled funding.^73^ Indeed, formal and informal user fees act as barriers (disincentives) to accessing PHC, particularly for the poorest; for those who do choose to pay, user fees can lead to financial hardship.^74^, ^75^ The frequent argument that fees paid at the time of service prevent frivolous use of health services without causing harm is not substantiated by existing evidence.^76^ Requiring even small payments can dramatically reduce use of highly cost-effective interventions.^77^ User fee exemptions for the poor and vulnerable can mitigate access barriers, although experience in LMICs suggests they can be challenging to implement, as it is hard to identify the poor effectively and the criteria are often gamed or applied inconsistently.^78^

The sixth is reduced budgetary allocations to health due to the economic impacts of COVID-19. Budget allocations to health were falling in LICs and lower-middle-income countries even prior to the COVID-19 pandemic.^33^ As the COVID-19 emergency phase ends, many countries can be expected to experience falling government budgets.^29^ For example, the South African Health 2021 Medium Term Expenditure Framework budget was reduced by almost 10% over one budget cycle as a result of the post-COVID budgetary restrictions.^79^ Austerity budgets following previous crises have had very harmful effects on health budgets.^80^

The seventh is difficulty in pooling resources. The incremental nature of UHC expansion often leads to the development of multiple risk pools as different programmes evolve to cover different population groups.^81^ Fragmentation of revenue streams is also an obstacle to pooling, with general tax revenues collected and used through the budget system and often disbursed as input-based budgets to maintain the health delivery infrastructure, and other sources of revenue pooled in an off-budget fund (such as a public insurer) and disbursed as payments for services. Once multiple pools have been established, it can be politically difficult to merge or integrate them, as this requires trade-offs with some interest groups losing, or perceiving to lose, their advantages.

The convergence of these problems in mobilising and pooling funds for health has resulted in large variations in levels of government health spending, even among countries with similar economic status (appendix p 12).

### The Commission's vision for resource mobilisation and pooling

The Commission's vision for people-centred financing of PHC requires an adequately financed health sector funded by expanded public and pooled sources that protects people from financial hardship when seeking care and promotes equity.

Achieving the Commission's vision involves reducing out-of-pocket payments (including removal of user fees for PHC) and replacing them with other types of pooled public funds that are raised through progressive means. In line with progressive universalism, the Commission argues for an explicit focus on addressing inequities.^82^

### Mobilising new resources for government spending on health

One of the greatest sources of increased government health expenditure has been economic growth, rooted in conducive macroeconomic conditions.^83^ However, in the near term this is unlikely to be sufficient to secure the additional financing needed due to the adverse global economic outlook generated by the COVID-19 pandemic. Below, we discuss other strategies for mobilising new public resources for health and investigate how countries can translate increased general revenue into increased funding for health. We also comment on the potential for the health sector to improve the efficiency of spending of its existing resources.

#### Increasing the overall envelope

Governments and health systems have several options for increasing resources for health, including expanding taxation, expanding social health insurance contributions, and increasing borrowing.

The primary means of expanding resources for health in LMICs is to expand the overall available government revenue collected via taxation. This can entail improving collection of existing taxes, increasing the tax base, and expanding the number and types of taxes levied. LMICs, however, face significant challenges when collecting tax revenues. Constraints including lack of infrastructure (including information technology systems) and administrative challenges, such as incomplete property registers, the size of the informal economy, or the inability to trace transactions within it. These limit the capacity of tax agencies, often already weakened by an insufficient number of skilled staff and limited technical capacities.

Expanding LMICs' national taxation capacity therefore requires strengthening various institutions, systems, and skills. Countries also need to decide on the appropriate mix of direct (income), indirect (for example value-added tax) and other taxes (including trade taxes), in which there will be a trade-off between administrative complexity and equity. Income taxes can be more progressive than indirect taxes, but require more elaborate systems to enforce compliance.^84^ More global comparative data on taxation across countries is now available than ever before and is vastly better than what was available 10 years ago.^85^

LMICs could also focus on taxes that directly effect health outcomes, which are often perceived as politically more acceptable.^86^ The health sector is increasingly active in advocating for health taxes or excise taxes on unhealthy products such as tobacco, alcohol, and sugar-sweetened beverages. However, the primary purpose of these taxes is to improve population health status by creating disincentives to the use of harmful products. Therefore, where excise taxes are successful, they should eventually result in reduced revenue. However, in the meantime, these can raise revenue that can be earmarked for health if political economy considerations and budgetary rules are aligned.^87^ According to the World Bank, an increase in excise taxes on tobacco, alcohol, and sugar-sweetened beverages that would result in a 50% increase in prices could raise the tax–GDP ratio by an average of 0·7 percentage points in low-income countries and LMICs.^72^ Other taxes could also be explored for the health sector, such as taxes on transport and airline levies^88^ or on carbon emissions.^72^ Overall, the potential additional revenue that these taxes or levies could bring seems to be limited (up to a maximum of 2·3% of actual spending on health).^89^ Earmarking these taxes to cover health expenditure is possible^29^ although it might be resisted, particularly by finance ministries,^86^ because of the inflexibility it introduces into the budgeting and planning process.

A tax on payroll, or compulsory social health insurance (rather than voluntary as is the case of many community-based health insurance schemes) is also used as an earmarked tax for health. Indeed, many countries are working towards UHC through expansion of social health insurance,^90^ as it holds the potential to improve equity by increasing pooling and introducing pre-payment.^91^ However, empirical evidence raises some concerns about the introduction of compulsory health insurance contributions in low-income countries.^92^ First, the necessary conditions for raising additional social health insurance funding are seldom met because most do not have full (or nearly full) employment and few jobs are located in the formal sector, registered, and therefore taxable.^90^ Second, social health insurance might not be feasible due to the high costs of setting up and administering the collection of contributions^92^ and might take a long time to lead to the achievement of UHC, as was the case in Germany for example.^93^ Whether the revenue raised can outweigh the operational costs has seldom been analysed.^94^ When it has been examined, the findings are inconclusive.^90^ Third, the cost to employers of social health insurance premiums might also have a negative effect on the labour market, reducing the probability of employment in the formal sector as well as wages.^95^ The changing nature of work, including demographic changes and structural changes in employment, pose a challenge to the feasibility and sustainability of mobilising resource for health through employment-based models such as social health insurance.^96^ Finally, social health insurance does not necessarily support progressive universalism, as it can redistribute resources towards wealthier segments of the population. For example, general revenues can be used to subsidise social health insurance that predominantly serves upper-income groups rather than ensuring that subsidies are used to extend coverage to vulnerable segments of the population.^90^

Whatever tax is chosen, political economy factors, both internal and external, as well as the structure of LMIC economies, always threaten the feasibility of taxation reforms (panel 6).

Panel 6 **Political economy challenges of expanding taxation in low-income to middle-income countries (LMICs)**

An increase in taxes is never welcomed by taxpayers. Introducing taxation reforms involves complex political economy concerns, including:

**Internal factors**

Taxation reform involves extensive bargaining among different actors, including governments, taxpayers, specific industries and sectors, tax intermediaries such as accountants and tax advisers, and revenue collection organisations.^97^ As such, the process of bargaining with all stakeholders will be central to decisions about the design of tax regimes. Certain groups might be disproportionately affected by different taxes—and this might constrain the feasibility of adopting them. For instance, although a property tax could be an effective way to increase tax revenue, these tend to affect wealthier people who are closely connected with political decision makers.^98^ Similarly, sugar-sweetened beverage taxes, although important for chronic diseases, affect particular agricultural and industrial sectors.

**External factors**

LMICs' ability to raise taxation revenue is currently substantially limited by existing bilateral tax treaties and the associated curtailment of taxes on multinational companies, tax evasion and avoidance, loss of potential revenue from extractive industries, and tax exemptions and incentives given to investors.^98^ The G20 finance ministers' recent announcement of a minimum 15% tax on multinationals in countries where they operate is a positive step towards addressing this issue.^99^

**Structure of the economy**

LMICs typically have a large informal sector which is difficult to tax. Further, the economy might be comprised of many small-scale firms that are more likely to be dependent on a few natural resources or commodities. LMIC economies also often rely on foreign aid, which is not taxable. These factors limit tax collection and the tax base.^84^

Generating additional resources from taxes, including for health, is certainly possible—but it requires a tailored strategy in each country and it takes time. Developing a tax reform strategy requires understanding the types of taxes and rates that are feasible given the political and economic structure of a country, and a whole-of-government effort to successfully push to increase the overall revenue envelope. For example, Tunisia increased its tax revenue from 18·2% of GDP in 1990, to 32·1% in 2018.^100^ It is now nearly double the average of 16·5% across Africa in 2018.^101^ This was achieved through the implementation of multiple policies, including: countering harmful tax practices, preventing tax treaty abuse, making dispute resolution mechanisms more effective, tackling money laundering, and cracking down on tax evasion. Aside from the technical solutions that were adopted, political resolve was essential in driving these reforms, and their success.^100^, ^101^

Another way to increase the budget available for health is to use government borrowing, as a short term approach. In times of crisis, government borrowing can have an important temporary role in sustaining health and social spending. The ongoing global COVID-19 pandemic, and the associated global recession represent just such a situation. Some reflections on the challenges of mobilising revenue in times of crisis are presented in the appendix (p 3). Deficit financing is typically considered more suitable for capital, rather than recurrent, expenditures. However, it can have a crucial role in supporting countries to emerge from crises while helping to mitigate their social consequences. In the first quarter of 2021, the International Monetary Fund, World Bank, and OECD all called on countries to take on temporary debt to finance human development. These institutions have emphasised the important role of the health sector in dealing with the pandemic, as well as in turning around the COVID-19-induced economic crisis. They also note the importance of not withdrawing social support, health spending, or fiscal stimulus in the midst of a pandemic.^102^

The increased borrowing will need to be accommodated by fiscal adjustments in the future. Most countries are projected to go through extended periods of fiscal adjustments in the coming years; in some places, the overall government spending envelope is expected to shrink, as will discretionary spending as a share of government expenditures, as debt service requirements grow.^29^ Grants, concessionary loans, and favourable debt treatment, including debt service suspension and restructuring, will be vital for many LMICs in the coming months and years. Government debt can help maintain liquidity and solvency and support to the spending needed to recover from COVID-19. Although LMIC governments might be reluctant to acquire more debt, it represents a way to respond to crises while expanding support for the health sector. Ultimately, investing in health will benefit the economy, enabling countries to get out of debt more quickly.

Another way to increase the overall envelope is by bringing donor funding on-budget and aligning it with national priorities. The COVID-19 pandemic (just as with the 2016 Ebola epidemic) showed that investing in health in LMICs has global significance.^29^ Donor funds not only need to increase, but they also need to be used in ways that align with government priorities, after the principles of the Paris Declaration.^103^

#### Translating more resources overall into additional funding for health

Whichever strategies are selected by governments, and specifically ministries of finance, to secure additional resources, the next step is to ensure that the new resources are invested in health. The widespread economic impact of the COVID-19 pandemic has provided clear evidence of the close connection between health and economic prosperity and should strengthen the case for increased investment in health. However, ensuring that adequate resources are allocated to health requires continuous efforts. The allocation of resources to health is an intensely political issue. Multiple political economy factors have a role in whether health is prioritised.

The first factor is competing demands. In the face of infinite needs, different sectors in a given country must compete for limited funds. How much goes to health depends both on how much value is placed on health and on what other challenges the country faces. A country struggling with active conflict might prioritise military spending over health. For example, Mali's budget allocation to health decreased from 2016 in the face of a domestic terrorist insurgency.^47^, ^104^ Health advocates must both understand the competing demands and determine how best to present health as an economically sound and ethical investment.

The second is who benefits from funding health. In most countries, small elite groups of politicians and financiers wield the most control over budgeting and financing decisions. Unless they see benefits in investing in health, little might change. In Turkey, for example, there were nine unsuccessful attempts to establish a national health insurance system for UHC before 2003. Those attempts were blocked in part by legislative gridlock in the parliament and opposition to new legislation by the Constitutional Court.^105^ UHC was eventually adopted after the rise of the AK party, whose platform centred, at the time, on opposition to persistent inequalities^106^ (see section 6 for further details).

Third is that in certain contexts, health must become part of a popular political agenda in order to be prioritised. This can occur during political transitions, such as following the fall of dictatorships,^55^, ^107^ the rise to power of an insurgency (as in Ethiopia),^108^ or as a result of crisis such as COVID-19 (see section 6 for further details).

Whilst this list is by no means exhaustive, it illustrates the need to take political economy considerations into account in tandem with technical solutions to gain support for health.

### Better pooling of existing and new resources

Whether or not total health spending increases, a shift from out-of-pocket spending towards pooled arrangements can radically improve the equity and efficiency of health financing.

A defining characteristic of the health sector is the high degree of uncertainty associated with health needs, which vary across populations, over time, and across geographies. This uncertainty makes it necessary to pool risk across populations to protect individuals from financial hardship if they find themselves in the unlucky group that requires expensive health services. Redistribution of resources from people and places of lower need to those of higher need is more effective in larger, more diverse pools. Pooling can occur either in government budgets (at central or decentralised levels), through compulsory insurance schemes, or, potentially, through virtual health insurance pools supported by digital technologies. The opportunities, and challenges, of virtual pools are discussed in the appendix (p 34). Many countries are moving in the direction of merging or consolidating multiple pools to gain the benefits of more effective risk-pooling, which include better redistributive mechanisms and greater equity, stronger purchasing power, and increased administrative efficiency.^109^

### Better spending

Inefficiencies in health spending exist. The 2010 World Health Report stated that there were inefficiencies equivalent to 20–40% of health spending, and in the OECD between 20% and 50% of health expenditures might be wasted due to inefficiencies.^73^, ^110^ Inefficiency can be allocative (that is, spending money on the wrong interventions, such as high-cost, low-impact health services). Inefficiency can also be technical (failing to get the maximum output from available inputs, for example when patented drugs are purchased in lieu of generic drugs). In theory, addressing these inefficiencies could be a source of resources available to the health sector by releasing resources while maintaining (or even increasing) output. Efficiency can also improve if greater outcomes are achieved with the same inputs; however, this does not increase fiscal space. Efficiency reforms should not be seen as a way to either balance budgets or identify spending cuts in health. However, they do form part of the wider effort to use available health resources to improve health outcomes, and to persuade ministries of finance to increase the health budget.

Improving either technical or allocative efficiency is a complex task, fraught with technical and political hurdles, and requiring time. A review done for this Commission^111^ showed that the efficiency gains that could be achieved in practice through reforms addressing inefficiencies are likely to be smaller in magnitude than suggested in the 2010 World Health Report.^73^ Furthermore, the available evidence on the timing and feasibility of efficiency-focused reforms is scarce and not generalisable across countries. The methods used for the literature review, together with an overview of possible efficiency reforms identified, are presented in the appendix (p 37).

Although the effect of some reforms focused on enhancing spending efficiency might be immediate (such as requiring the purchase of generic rather than branded medicines, or use of bulk tenders for medicines or medical equipment), many others may take years to reap benefits. Many inefficiencies, such as leakage due to corruption or fraud, are structural; tackling them requires addressing historical precedents and social norms in addition to administrative processes.^112^ Further, it should be noted that addressing some inefficiencies can require upfront investments. This might be one area where digital technologies can prove (positively) disruptive, as long as they do not create new fragmentation (appendix p 16).

Finally, although the focus here has been on whether inefficiencies could lead to financial savings (as has been argued by many finance ministers), ministries of health should be working to improve the value for money of their spending. This outcome might be realised through a decrease in cost, as long as the outcome (including quality of care) remains the same. It might also involve an increase in cost with an accompanying improvement in outcomes or simply an improvement in outcomes, with the cost remaining constant.

#### Conclusion

The need to shift away from out-of-pocket spending towards pooled public funding is urgent, yet the reforms to increase resource mobilisation and pooling necessary to achieve this objective will take time to design and implement. Investments in strengthening the tax base, expanding the types of taxes levied, and tax collection capacity—all of which fall outside the purview of the ministry of health—will be essential. Digital technologies might hold some promise (for example through virtual pools), although caution is warranted in ensuring that these align with UHC objectives. Improving the efficiency of spending can help, but is not the primary way to create more fiscal space for health.

## Section 4: People-centred allocation of resources to PHC

### The Commission's vision for a people-centred approach to allocating sufficient resources to PHC

In the Commission's vision, each country's health sector strategically uses appropriate policy tools to direct sufficient resources to PHC to enable a universally accessible system that provides quality services in line with a benefit package appropriate to the level of care. To do so requires mechanisms for funding, budgeting, and financial management to ensure that those resources reach frontline providers and platforms. It will also organise the service delivery system to pull resources to PHC. By doing so, countries can support the delivery of, and equitable access to, people-centred PHC. Key messages from this section are presented in panel 7.

Panel 7 **People-centred allocation of resources for primary health care (PHC)–key messages**

• Increasing the allocation of health resources to PHC is a political decision; it might involve redistributing of resources—in absolute or relative terms—away from other sectors, or within the health sector away from hospitals.
• Increasing budget allocations to PHC does not guarantee that resources reach frontline services; protecting PHC allocations to the point where they reach frontline providers requires clarity, active steering, and accountability mechanisms.
• Making allocations to PHC more visible in health budgets is an important way to improve tracking of existing resources, secure additional resources, and highlight the importance of essential public health functions.
• A range of policy levers are available to increase and protect allocations to PHC. Public finance management tools, particularly those that strengthen budget formulation and execution, can be used to increase PHC budgets, make them more visible in the public finance management system, and ensure that resources reach frontline services. Service delivery arrangements can be used to pull resources to PHC. These arrangements include explicit service standards and guidelines, new configurations of teams, and effective referral systems. In some instances, new cadres of frontline PHC providers have enabled more resources to be directed to PHC. All these improvements can have the effect of stimulating demand for PHC and, with the right financing arrangements, drawing more resources to this level.
• Multiple tools can be applied at the same time. Many of them require a clear and context-specific operational definition of PHC. Applying public finance management levers requires engaging with various health and public finance management system capacities.
• Institutional responsibility for PHC is typically fragmented across ministry of health departments; as a result no single unit is in charge of securing funding or held accountable for progress. Although it is not necessary to create a new operational unit for PHC to ensure that PHC is prioritised, it must be clear where responsibility for budgeting and planning for PHC lies in the ministry of health.

The objective of resource allocation for PHC is to ensure that sufficient resources are directed to, and reach, frontline providers working in PHC platforms, including at community level, as well as supporting essential public health functions. Whether or not the overall fiscal space for health is increased, deliberate efforts can be made to allocate more resources to PHC from existing health budgets, either through the budget process or by strengthening budget execution to ensure that available resources for PHC effectively reach PHC platforms and providers.

Putting people at the centre of these arrangements entails allocating resources for PHC based on population needs, rather than allocating resources to facilities, inputs, or vertical programmes. To achieve equity in this allocation process means prioritising the needs of people with the lowest socioeconomic status and least-served geographic areas. This section of the report explores the forces that can draw resources away from PHC, and describes policies that support allocation and protection of resources for PHC. It also addresses the special case of allocating and securing financing for essential public health functions. We acknowledge that various other policies also indirectly influence allocations to PHC, including some key PHC design elements and how PHC is linked to higher levels of the health system. Provider payment methods, which are addressed in greater detail in section 5, are central to this.

### How resources for health are allocated

Allocation of resources to health in general, and to PHC in particular, occurs in different ways in different systems of government. In centralised budget systems, the collection and allocation of funds takes place centrally with, typically, the Ministry of Finance allocating a set amount, based on multisectoral budget negotiations, to the Ministry of Health. Although some of these resources might be ring-fenced for specific purposes, many decisions about allocation within the health sector are the responsibility of the Ministry of Health. It decides how to allocate the available funds to geographic units (such as provinces) or to levels or platforms of care, such as PHC.

In decentralised systems, decisions about resource allocation—across sectors and within the health sector—are made by local authorities. This gives the opportunity for resource allocation decisions to be shaped by local needs, disease patterns, and priorities. However, it can also lead to a focus on financing services that are popular or visible, rather than those that bring the greatest population health benefits. A key challenge for decentralised systems is that allocations to health in general, and PHC in particular, might be less visible and traceable at national level than when they are presented as part of a single centralised budget. Therefore, decentralised systems might require additional policy tools (such as common budget structures and reporting systems) to protect, manage, and monitor the use of resources for PHC. The countries of Kenya, India, and the Philippines are all examples of devolved decentralised systems where allocations to, and power over, health are substantially determined at subnational level.

Whether a government is organised as a centralised or decentralised system represents a broader political choice that is made outside of the health sector. However, the health sector must adapt its allocation processes to match the wider political structure of the country.

As with allocations to health in general, there is no definitive answer about how much is the right amount to allocate to PHC in any given setting. The key issue is estimating the resources required to finance a PHC package that is universally accessible, places minimum financial burdens on users, and that is aligned to macrofiscal capacity. The analysis of the relationship between PHC spending and coverage of priority interventions presented in section 2 showed that there is substantial variation in the level of coverage achieved for a given spending level, particularly in LMICs; section 3 discussed some evidence about spending targets.

Costing exercises can help to inform these decisions. However, costing requires a clear operational understanding of what constitutes the PHC platform and services in that particular setting. South Africa has used numerous approaches to cost its PHC system, including normative personnel and infrastructure costing, modelling visit rates and unit costs, complex methods of costing specific disease groups and treatment interventions, comparisons of facilities using cost, efficiency, and quality indicators, and actuarial approaches in designing capitation payments.

### The politics of resource allocation within the health sector

Budget allocation processes are influenced by a range of political forces operating at all levels. Problems, such as those created by political patronage systems,^113^, ^114^ can be exacerbated in decentralised systems where local political incentives, power relations, and special interests can carry more weight than the policy priorities of a central Ministry of Health. Vested interests at central level might also skew resource allocation.

PHC faces particular problems in attracting sufficient resources because it typically does not elicit strong political support within the health sector and may be excluded from the benefit package of particular insurance arrangements. Responsibility for elements of PHC might be fragmented across agencies or technical departments, with no clear responsibility or accountability for delivering on policy commitments to improve PHC.

Other factors combine to limit the political attractiveness of PHC. In particular, the people who would benefit most from expanding and improving PHC (for example, children, women, and people living with chronic conditions) typically have little political power. Furthermore, when PHC is done well, it keeps people healthy and thus becomes nearly invisible.

In contrast, allocating resources to hospitals is more politically appealing. The hospital is a highly visible symbol of the health system. A hospital is the site of more, and more expensive, technology, which is perceived as beneficial and important. Hospitals also employ specialist physicians, who tend to have higher professional status and political connections than PHC workers. Hospitals engage in readily apparent medical education and other training activities, and employ large numbers of support staff. They tend to be located in urban areas, closer to decision makers, and cater to wealthier population groups who wield significant political influence.

Therefore, when limited resources are available for health, hospitals and other secondary and tertiary care initiatives are often more successful at securing them. However, this imbalance undermines the efficiency and equity of the health system as a whole. In this section, we simply note that the process of securing budgets for PHC is not merely technical but is also influenced by political forces. Section 6 addresses the political economy of financing PHC in much more detail.

### Forces that impede allocation to PHC and the policy levers that protect resource flows

**Figure 9:**
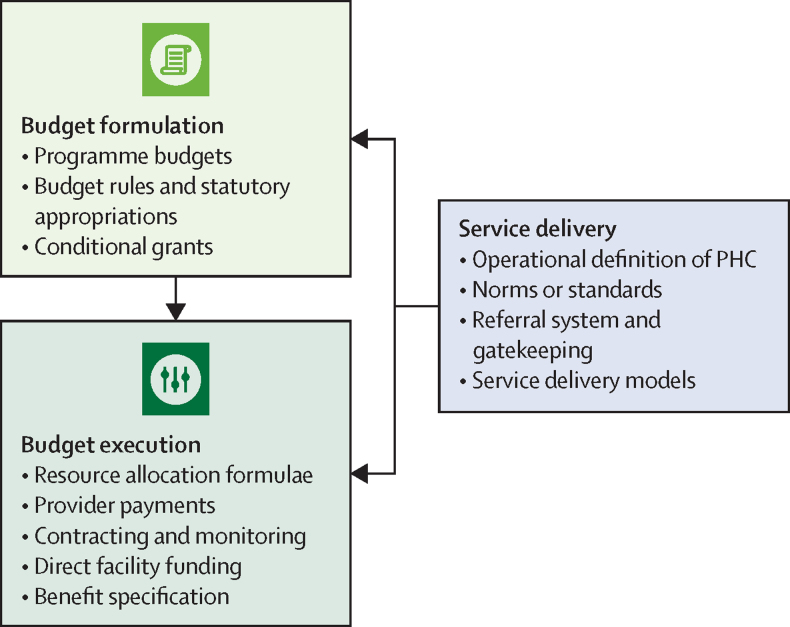
Policy tools to increase and protect resource allocations to PHC PHC=primary health care.

A number of technical factors can also impede increased allocations to PHC or divert funds away from PHC. Addressing these is essential to protect resources for PHC and ensure they reach the frontline. This can be done through careful system design and, in particular, the use of various categories of policy tool (figure 9).

The first category of tools is related to budget formulation. Programme budgeting, budget rules and statutory appropriations, and conditional grants influence how budgets are made and aligned with policy priorities. A second category of tools relates to budget execution, or the effectiveness with which funds flow through the system. These include the use of population-based resource allocation formulae and direct facility funding. Some purchasing tools, such as benefit specification, provider payment, and contracting arrangements, also fall in this category because they influence the flow of funds. The third category concerns how PHC services are organised and how they relate to and interact with the rest of the health system. This category includes the adoption of an operational definition of PHC, the use of norms and standards to establish resource requirements, service delivery models, and effective referral and gatekeeping functions.

The first two categories, budget formulation and budget execution, are part of public finance management processes; for their part, service delivery arrangements will influence both the estimation of budget needs and the routes through which funds move through the health financing system. These policy levers clearly are not mutually exclusive; indeed, they can be undertaken in parallel. We outline a short overview of each of these levers in the following sections. The capacities (of the health system and broader public finance management processes) required to deploy these tools are presented in the section's conclusion.

#### Budget formulation

The primary instrument for allocating public funds to health programmes is the government budget. The budget formulation processes used by many governments can sit at odds with the unpredictability and complexity of resource needs for health,^115^ with budget formulation and stated policy objectives often disconnected.^116^ In practice, the process of budget formulation is commonly reduced to incremental adjustments to the previous year's budget. Health budgets also suffer from a lack of credibility, as they are often believed to be based on inaccurate data and incomplete budgeting analysis.^117^ Even when PHC is nominally prioritised in national policies and plans, budgeting structures and processes can inhibit the allocation and flow of funds to PHC providers. In most countries, PHC is not a visible line item in the national budget, making it hard both to pinpoint how much is being allocated and to monitor whether PHC funds are actually received. Instead, PHC budgets are often embedded in, and absorbed by, the budgets of hospitals or local governments. Budgets frequently follow a line-item structure, with resource flows tied to inputs rather than to activities, levels of care, or population health needs. In these systems, resources get to frontline PHC providers mostly through in-kind, easily quantified provision of medicines and supplies, and staff salaries. Establishing appropriate programme budgeting can help to make financial allocations to PHC more visible in the budget.

Oversight of resource allocation to PHC is especially complicated when multiple financing agents and purchasers are involved. This occurs, for example, if a social health insurance fund is administered separately from the government health budget. The social health insurance fund is then outside the influence of the ministry of health and different provider payment mechanisms might apply. Likewise, in decentralised systems, central authorities have little oversight or influence over budget allocations by decentralised authorities. In China, for instance, PHC is the responsibility of two different agencies: the local health bureau for essential public health services and the social insurance office for medical services. Each has a separate budget and uses different provider payment methods (see section 5). In the Philippines, fragmented funding makes it difficult to prioritise PHC (panel 8). As noted in section 3, donors can contribute to fragmentation of budgets when their substantial contributions remain off-budget and separate from national plans. Ethiopia provides an interesting example of a national initiative to harmonise all sources of funding on one budget, which is called One Health, One Plan, One Budget.^118^

Panel 8 **Multiple funding flows and fragmented budgeting processes for primary health care (PHC) in the Philippines' decentralised system**

The Commission's case study on the Philippines examined the arrangements for funding PHC in a decentralised political system. Health is a devolved function in the Philippines: local governments receive a share of general tax revenue and are also able to raise revenue through local taxes. Decisions about the allocation of these funds between health and other sectors are taken at the municipal level. In addition to a share of these general funds, health receives some additional supply-side funding from the central government for infrastructure and for employment of essential staff. Local government also receives reimbursement for PHC from PhilHealth, the national social health insurance programme.

Resources are allocated among sectors through a process that is led by the elected municipal mayors. Budget rules provide for mandatory shares of the budget to some sectors, such as gender and development, but no such rule is imposed for health. The local health plan is the instrument used to consolidate PHC funds from different sources. But priorities can diverge between political and technical participants.

The fragmentation of PHC funding is exacerbated by the different timelines for the national and municipal planning cycles, and different procurement rules applied to different funding sources. In practice, reimbursement from PhilHealth is unpredictable due to administrative complexity; this revenue is also affected by low claims by beneficiaries who are unaware of their entitlements.

The outcome of this process is that municipalities spend only 7% of their budget on health, against a benchmark of 15% set by the central health ministry. Two new initiatives under the Universal Health Coverage Act are intended to help consolidate and align resources for PHC: the development of a new primary care provider network, and a special health fund that is pooled at the province level. Local actors remain sceptical about whether these technical solutions can address the political issues of resource allocation.

Several policy levers are available to address these problems and strengthen budget formulation.

(1) Programme budgets: this approach organises the budget according to programmes (eg, a service or group of services) rather than by inputs.^119^ Programme or performance-based budgeting serves to clarify programme objectives, and improves monitoring and accountability, as each programme can also have associated performance measures.^120^, ^121^ South Africa uses a programme-based budgeting approach alongside a higher-level item classification, and has a specific budget for PHC comprised of seven subprogrammes within the District Health Services programme, with substantial flexibility in shifting funds. This standardised programme structure applies across all provinces and districts. Each PHC facility can spend up to their budget limit in line with an agreed spending schedule. The five basic PHC subprogrammes (clinics, community health centres, community based services, district management, and other community services) comprise 19·2% of total public health expenditure (31·1% when including the HIV or AIDS and PHC facilities subprogrammes). Budgeting for the PHC programme can be a means of creating greater visibility and protecting resources for PHC. When applied generally across a government, programme or performance-based budgeting seems to be effective in improving resource allocation to health and supporting more productive negotiations between the Ministry of Finance and line ministries. Input-based budgets maintained alongside programme budgets facilitate financial control.
  However, programme-budgeting requires both considerable budget management capacity within the spending institutions and good costing information. It might also lead to new rigidities in budget execution if controls are carried over from input-based budgeting.^120^, ^122^ Although there is scope for expanding programme-based budgeting, there is little experience in how to address specific demands of the health sector.^123^ Programme-based budgeting for PHC also assumes that the package of services, as well as the level at which given services are to be delivered, are defined. As outlined in section 2, this is a key challenge for policy makers. Although experience with programme budgets is growing, there is still much that needs to be tested.^120^
(2) Budget rules and statutory appropriations: this is another approach to ensure sufficient budgets for PHC. Budget guidelines can mandate minimum budget shares for specific sectors (as in the case of the gender and development sector in the Philippines; panel 7). Statutory appropriations are a legally-mandated standing budget provision (so-called appropriation), which is not dependent on the passing of a legislative appropriation bill. For example, in Nigeria, the National Health Act earmarks 1% of the federal government's consolidated revenue to fund the Basic Health Care Provision Fund.^124^ Such rules and statutory appropriations have the advantage of protecting part of the health budget from political processes.
(3) Conditional grants: in both centralised and decentralised systems, the central government can influence resource allocation towards policy priorities through conditional grants that impose restrictions on the use of funds. Conditional grants can, additionally, be used to create incentives for specific spending (eg, matching rules for National Health Mission grants in India; panel 9), impose governance requirements (eg, audits or reporting requirements), or be supplemented with performance targets (eg, Plan Nacer and Sumar in Argentina).^126^ These can be used to influence devolved or other local units to invest in certain programmes, leveraging centralised funds to expand overall PHC resources.
  Panel 9 **Conditional grants influence resource allocation in India**
  In India's system of government, health is the responsibility of the subnational state governments. The Commission's case study on India examined mechanisms used by the central government to encourage states to invest in primary health care (PHC).^125^
  The National Health Mission, which was previously known as the National Rural Health Mission, is the main central programme for strengthening PHC. Under the National Health Mission, the federal government created a number of mechanisms to encourage states to fund PHC, including:
  • A matching rule for distribution of central level grants, which initially required states to contribute 15% of the total funds; the required contribution increased to 40% in 2016–17.
  • A planning process which included allowing the central government to conduct detailed reviews of PHC plans and budgets.
  • A system of performance assessment and accountability that made the release of a portion of the approved resources contingent on achieving a given level of performance. The performance-based component increased from 10% to 20% of the total National Health Mission funding and conditions were revised in 2018 though not yet implemented due to COVID-19 disruption.
  Over the period 2008 to 2019, state spending on PHC spending did increase. However, while the central government influenced the pattern of PHC spending, state-level respondents did not feel that these policy levers necessarily increased effective use of funds.

#### Budget execution

Budget execution processes in LMICs are often highly bureaucratic and focus on financial accountability rather than achieving outcomes. This focus on financial control might be due to concerns about corruption. The many stages of fund disbursement, as well as frequent delays in approval and release of funds, tend to reduce the amount and timeliness of funding that actually reaches PHC facilities and providers. The more remote the health facility, the fewer resources eventually arrive. This is not just an allocation problem; significant leakage and delay can occur during the many steps involved when disbursing funds from the central ministry of finance to a regional authority, then to local levels of government, and at last to facilities.^127^, ^128^, ^129^ A particular challenge for PHC is that discretionary item allocations suffer the most from leakage and other execution problems; this leads to few resources being made available for the general operating costs of PHC facilities.^116^ Low levels of provider autonomy (section 2) creates further rigidities in how resources are deployed. Various policy tools are available that can help to avoid or ameliorate these difficulties.

(1) Resource allocation formulae: These are used to allocate resources (among geographical units and levels of care) are policy levers that can promote equity in allocation. In its simplest form, a resource allocation formula allocates an equal per-capita amount across the recipient units. These formulae can be refined by adding adjustments, such as for differing health needs or local cost differences.^130^ For instance, health budgets in the English National Health Service are allocated among geographical units using a needs-adjusted formula. This approach inspired many countries to develop similar arrangements. Resource allocation formulae can also be used as part of budget rules and conditional grants.
  Resource allocation formulae have been effective in directing and protecting resources for PHC in both Chile and Brazil. In Chile, a per-capita amount for PHC is allocated to municipalities to operate PHC facilities (panel 10).^55^ In Brazil, the budget for the Family Health System is allocated to municipalities using a formula (panel 11).^107^
  Panel 10 **Capitation-based resource allocation in Chile**
  The Commission's Chile case study examined the development, design, implementation, and effect of the capitation-based system for allocating resources to primary health care (PHC) in Chile.^55^
  After its return to democracy in the 1990s, Chile replaced a system based on fee-for-service for PHC with one based on a per-capita-based allocation using a resource-allocation formula. The payments transferred to local governments are calculated according to the size of their population, adjusted to take account of the age, poverty, and rurality of a population. The local authority then allocates resources to PHC providers to cover the costs of salaries and services in accordance with the government's Family Health Plan. By 2019 this per capita allocation represented nearly 65% of the overall financing transfers for PHC. The payment system both serves to allocate resources to geographical units and is a means to pay providers. This capitation-based allocation has improved equity through the provision of more resources to poorer municipalities.
  One notable limitation of the system, however, is the low degree of financial integration between PHC and higher levels of care. Because diagnosis-related groups are used to pay for hospital services, clinical coordination and integration of care are more difficult. The risk adjustment mechanism is also considered to be quite crude, limiting possibilities for redistribution.
  Panel 11 **Brazil uses a population-based mechanism to allocate resources to the frontline**
  The Commission's Brazil case study set out to understand the health financing arrangements that shaped the Family Health System.^107^ The Family Health System scaled up the provision of PHC through multidisciplinary teams who provided community-based services in a geographical area, shifting the way health-care services are delivered in Brazil.
  Financing of the Family Health System was through a direct transfer from the federal level to municipalities, known as the Piso da Atencao Basica (meaning Floor for Basic Care). The transfer was calculated as a fixed per-capita amount based on municipal population and a variable component linked to federal priorities, including scale-up of the Family Health System model. From 2011, adjustments to the formula were introduced to allow more funds to be allocated to more deprived municipalities.
  Through this mechanism, regular and predictable resources were provided monthly to all municipalities for delivering primary health care (PHC). Adjustments to the Piso da Atencao Basica arrangements were made over time to encourage municipalities to adopt the Family Health System model, expand the scope of PHC services provided, and to address health inequalities. This approach to financing PHC had clear impacts on reducing inequality in funding; although this effect was mitigated to a degree by the requirement to have a substantial municipal contribution to PHC funding.
  Supported by these financing arrangements, the number of Family Health System teams grew from 2054 to 43 286 between 1998 and 2020, covering 133·7 million people (63·3% of the population). A number of studies point to the effectiveness of the family health services in improving access to health care, reducing health inequalities and improving health outcomes.^131^, ^132^
  However, with substantial disparities across municipalities in financial, administrative and technical capacities, inequalities across the country have persisted, and the availability of qualified health professionals in poorer and rural areas has constrained expansion of family health services. Recent developments, including fiscal austerity from 2016 onwards, political pressures to concentrate resources in specialised and hospital care, and the merger of financing blocks for PHC with secondary and tertiary hospitals threaten the achievements in financing an innovative PHC model over the past 20 years.
  A systematic review of the use of resource allocation formulae found that they enhanced equitable allocation of resources across provinces or smaller administrative units in Chile, Colombia, Zambia, and Zimbabwe.^133^ The appropriate mix of local and central financing matters for equity, as greater dependence on local resources undermines equity. Various other forces can also constrain the equity achieved through the use of resource allocation formulae, including: failing to account for local differences in costs; failing to account for the absorptive capacity of each geographic area; and overlooking the up-front investments required to expand service provision. Scarcity of consistent and robust data to inform the components of resource allocation formulae (eg, health status levels) at a given point in time can hinder the application and implementation of resource allocation formulae in LMIC.^134^, ^135^, ^136^
  Furthermore, simply applying resource allocation formulae does not ensure equitable distribution of resources. Okorafor and Thomas^137^ found that resource allocation formulae for PHC in South Africa were inequitable due to weak managerial capacity at lower levels of government, poor accounting for PHC expenditure, and lack of protection for PHC funds with regard to other service areas.
(2) Direct facility funding: where public finance management systems fail to effectively channel money to PHC platforms and providers, whether due to leakages or other reasons, a second policy tool is to give money directly to the PHC level. Under direct facility funding, a health-care facility receives some core funding from the central level directly into its own bank account, usually to enable the purchasing of medicines and other supplies, or to pay for operating costs such as utilities. When coupled with autonomy to spend according to local priorities and sound facility financial management, direct facility funding can improve efficiency and quality of care.^138^ Direct facility funding can also serve as a means to integrate multiple sources of financing at the facility level. For example, in Tanzania, the same formula is used to allocate resources from on-budget donor funds and the government budget (excluding salaries and medical supplies).
  This approach was used in Kenya in the late 2000s,^139^ Nigeria,^140^ Tanzania,^141^ Burkina Faso,^138^ and Uganda.^142^ Direct facility funding can ensure that funds reach the periphery; it can have the added benefit of making PHC providers more visible in the public finance management system, raising the profile of spending on PHC. More evidence is required, however, about the effectiveness of direct facility funding in actually channelling funds to facilities, and by association facilitating the removal of user fees.^138^, ^143^
(3) Strategic purchasing: This incorporates specification of the benefit package, selection of eligible providers, and choice of provider payment methods. Done well, strategic purchasing promotes effectiveness, efficiency, and equity in a health system. However, a poorly (or too narrowly) specified benefit package can cause patients, and therefore resources, to drift up the health-care system towards the hospital level. A benefit package that is too narrow, for example, might exclude routine management of chronic conditions. This can drive patients to seek care at levels of the system that are higher than necessary and expose them to risks of substantial out-of-pocket spending on medicines. However, various policy levers are available to address purchasing problems.

First is benefit specification. Having an explicitly defined and appropriate benefit package (that was developed using realistic costing) is a way to secure and protect allocations to PHC. In Thailand, for example, capitation payments for PHC are based on a defined benefit package. Its Health Interventions and Technology Assessment Programme is a world leader in health technology assessment. Benefit specification can also require co-payments when patients bypass PHC, which helps to direct patients to the appropriate level of care.

Second is provider payment mechanisms, which determine how money is paid from pooled resources to service providers. As such, the provider payment system has a substantial influence on how resources are ultimately allocated across providers. As is elaborated in section 5, adopting a payment mechanism that directs money to PHC, such as capitation, makes PHC expenditure more visible, more equitable, and helps to protect allocations. Poor design of provider payment systems can also incentivise bypassing PHC, such as when providers are rewarded for referring patients to higher levels of the health system. Resources can also be diverted away from PHC when mechanisms for paying providers have money following patients (for example, with fee-for-service payments) or are reinforced by an absence of gatekeeping or open-ended budgets at higher levels of the health system.

The effectiveness of a provider payment system as an allocation tool depends on the existence of complementary policies and properly aligned incentives. These include the coherence of provider payment systems across levels of care and payers, without which systems can decrease access, generate waiting lists, and overall decrease efficiency in the use of funding to provide care. It also can include health system organisation features such as gatekeeping and user incentives. Payment systems that constrain the budget at higher levels of the system (sometimes called closed-ended payments) can help to protect resources for PHC; this approach is used in Thailand. PHC subpools can also do this function; for example, in Taiwan an umbrella budget for the national health insurance has five subpools, including one dedicated to PHC provided at independent ambulatory care clinics.^144^

Third is contracting and monitoring. Contracts between purchasers and providers can be designed to include provisions that help channel funds to PHC and constrain resources from being paid to hospitals. For instance, in Estonia, the national health insurance fund guarantees a minimum amount of revenue (equivalent to the per-capita amount for 1200 individuals) for a defined list of PHC providers working in non-urban areas. This channels funds to PHC providers in sparsely populated areas and ensures that they can cover their fixed costs. More detail on Estonia's approach to PHC is provided in sections 5 and 6. Some contracts include volume caps on hospital payments, whereas others specify a facility level for payment for specific services. For example, in both China and Indonesia, purchasers will not pay for a service delivered at a level higher than appropriate. Contracts can also cover referral rules to limit unnecessary referrals to higher levels.

At times of crisis such as the COVID-19 pandemic, keeping money flowing through the system to frontline providers requires flexibility and adaptation of budget execution processes. Such flexibility also carries some risk. Keeping the focus on PHC is essential (appendix p 4).

#### Service organisation

In many health systems, inappropriate incentives for both patients and providers have been created that encourage the bypassing of PHC, resulting in pulling resources away from frontline providers. The incentives to bypass PHC are reinforced by the vicious cycle cited in section 1: weak political support for PHC leads to chronic underfunding, which affects the capacity of PHC providers to offer quality services; this, in turn, causes users to mistrust PHC and they bypass this level of care, turning instead to hospitals and specialists.

These incentives might not have been created with the intention of undermining PHC. However, without strategic and coherent organisational linkages among levels of care, resources can drift up the system. For example, when user fees are set such that the cost of PHC is similar to the cost of higher levels of care, patients may feel encouraged to bypass PHC. In places that lack easily accessible and trusted PHC to serve as a first point of contact, patients are also likely to seek care directly (if often unnecessarily) from specialists. Some innovative approaches intended to reinforce PHC by providing greater technical support to lower levels of care, such as the Health Care Alliances introduced in China, actually had the unintended effect of driving resources back to higher levels of the system.^145^ Various policy levers are available that, by improving the organisation of services, help to drive users and resources back to PHC:

(1) Organisational definition of PHC: a clear operational definition of PHC helps to steer resources towards it by defining what functions must be supported and by providing a category for tracking PHC expenditure. In Indonesia, for example, the national health insurance programme defines a clear set of PHC facilities (public and private) from which the PHC benefit package can be delivered.
(2) Norms, standards and guidelines: establishing clear norms standards and guidelines for PHC (including population coverage levels) can be a useful way to support allocation of resources to PHC. These declarations of how PHC is supposed to be delivered lend themselves to measuring resource requirements and tracking progress. The combination of a clear operational definition of PHC and service delivery norms makes it easier to cost PHC and to determine what level of funding is needed to deliver it. In Ethiopia, the expansion of PHC followed a stated objective: each PHC Unit (made up of one health centre and five health posts) would serve 25 000 people, including community-level services provided by two health extension workers per kebele (5000 people) in each health post. This clear standard enabled the government to estimate its resource needs and then translate the estimates into a costed plan that involved the construction of 15 000 health posts and 3200 health centres. The plan proved to be a powerful instrument when negotiating with both donors and government finance officials. The investments required for human resources to staff the new units were also determined based on the plan. Challenges remain, of course; these facilities are still developing the capacity to deliver the full set of PHC services, and there remain shortages of certain cadres of staff, particularly doctors and midwives.^146^
(3) Service delivery model: changing the service delivery model such that it effectively pulls resources to the PHC level. With appropriate financing mechanisms, service delivery arrangements that strengthen PHC can stimulate demand and help to pull resources to PHC. Different approaches have been implemented. For example, on the one hand, Brazil's Family Health System introduced multidisciplinary teams that operate at municipal level and are financed through the per-capita allocations paid directly to municipalities.^107^ Ethiopia, on the other hand, has developed a new model of PHC service delivery with the introduction of a new cadre. Health extension workers receive a year of preservice training and are paid a government salary to work from health posts at the village (kebele) level.^118^
(4) Referral systems and gatekeeping: protecting resource allocations to PHC includes directing patients and resources to the appropriate levels of care. Gatekeeping policies, in which patients must be referred from PHC providers to access specialist care, and measures such as empanelment and registration which link patients to providers, can help to influence care seeking and direct patients to use the lowest level of care at which their condition can be effectively managed. However, effective gatekeeping requires having functional referral systems in place so that patients can be rapidly sent to the appropriate level of care. Many health systems, especially in LMICs, do not have those systems in place. Where strict gatekeeping is not feasible, financial disincentives or incentives—such as higher co-payments for higher levels of care—can deter patients from bypassing PHC. In France, for example, a preferred doctor scheme was introduced in 2005, aiming to reduce the number of visits to outpatient specialists. Those who continued to self-refer to a specialist incurred a higher co-payment than those who were referred via their preferred doctor, which led to fewer specialist consultations.^147^

These and other policy levers will be more or less appropriate and effective depending on the specific context of each health system. Further, many problems exist within these approaches that have yet to be successfully addressed. Therefore, identifying new ways of improving the allocation of resources to support high-quality PHC service delivery remains a key challenge for policy makers.

### Financing essential public health functions

Essential public health functions and global common goods^148^ are part of PHC. They include activities whose importance has become particularly evident during the COVID-19 pandemic, such as public communication, disease surveillance, testing, contact tracing, support for affected individuals or communities, basic laboratory services, safe water supplies, sanitation, and hygiene. Mitigation of the impacts of environmental pollution and climate change on population health might also fall under the rubric of essential public health functions, with implications for health financing that is people centred. Financing population-focused interventions require different arrangements, sectoral engagement, systems, and capacity from individual primary care; however, many of the same principles and policy levers apply. Any national strategy for delivering essential public health functions should: create coherent priorities, clarify who is responsible for what, and align budgeting processes to ensure that these are adequately funded.^149^, ^150^ Financing for public health services frequently receives inadequate attention in government budgets; however, this financing is crucial for prevention and addressing the determinants of disease.

Essential public health functions can also suffer from fragmentation and ineffective organisational arrangements, with multiple payers involved and different government and non-government organisations responsible for delivery. Financing and formalising the regulation of private sector activity with population health impacts might be particularly important in considering public finance allocations to PHC at national and subnational levels. For example, subnational jurisdictions that benefit financially by taxing or partnering with polluting or extractive industries whose waste harms population health could reasonably be expected to fund essential public health functions or the additional services needed to mitigate these impacts.

In this Commission, we argue that a systems approach to financing is especially important for essential public health functions. This approach starts with clarifying who has responsibility for budgeting, planning, and ensuring that the essential public health functions are adequately resourced and delivered. As in other aspects of PHC, alignment of donor funding with national budgets and plans is also crucial. Decentralisation might result in insufficient resources for essential public health functions. Uniform national (and even regional) standards are essential; this requires system-wide coordination and standards across subnational units. However, the relative invisibility of these services and their benefits, and the required coordination of multiple sectors, might make them unappealing to local governments.

The policy levers detailed above can also be applied to securing financing for essential public health functions. For example, programme budgets can help to connect resource allocations to priorities and targets, whereas fiscal rules such as matching grants or resource allocation formulae can protect resources for essential public health functions. In some cases, however, it will be necessary to create new institutions to finance and deliver essential public health functions. For example, the Thai Health Promotion Foundation, which receives a share of the proceeds from national excise taxes, has responsibility for providing population-based health promotion activities. Private sector activities with an impact on population health should contribute to essential public health functions as appropriate.

### Institutional responsibilities and supporting functions

One challenge in financing PHC is the institutional design of many ministries of health, which are structured around health programmes (eg, department of maternal and child health, department of communicable diseases, and department of non-communicable diseases) rather than functions, levels, or service delivery platforms. Although it might not be operationally necessary or feasible to have a specific department for PHC, it is important that the responsibility for setting priorities for spending on PHC, and monitoring these, sits within an identified department or unit.

A key supporting function for resource allocation to PHC is the public financial management system through which budgets are developed and executed. Such systems are usually not unique to the health sector, and their strengthening may benefit from cross-sectoral initiatives to improve broader social sector financing and budget processes. Specific adaptation for the health sector might reduce paralysing bureaucracy. Strengthening public financial management, as well as PHC provider platforms' capacity to deal with finances, requires technical skill and the ability to collect, analyse, and interpret data on the population and its health needs to cost PHC benefits and required services. Another supporting function is the efficient deployment and management of the health workforce, including cadres at community level.

### Conclusion

When based on a clear operational definition of PHC, these budget formulation, budget execution, and service organisation policy tools can be deployed in concert to help ensure that sufficient resources are allocated to meet the needs of PHC that is people centred, and to protect the resources so that they reach the PHC delivery platforms. In Thailand, for instance, the budget for PHC is safeguarded through the combined effects of a defined PHC benefit package, ring-fencing of the PHC budget (and constraints on the budget for higher levels of the system), and capitation payment for PHC.

How can countries begin to move in this direction? We suggest that it begins at the budget formulation stage. Working towards developing a programme budget would be helpful to PHC, using other policy levers as necessary. At budget execution stage, a well-developed resource allocation formula can be a useful starting point for improving allocation of financing to PHC. Even a simple per-capita formula, with risk equalisation and performance and quality incentives added as the system develops, can begin to foster equity in universal coverage of a basic package of primary care services. For a formula to be effective, however, other reforms that link budget allocations to PHC are also needed.

**Figure 10:**
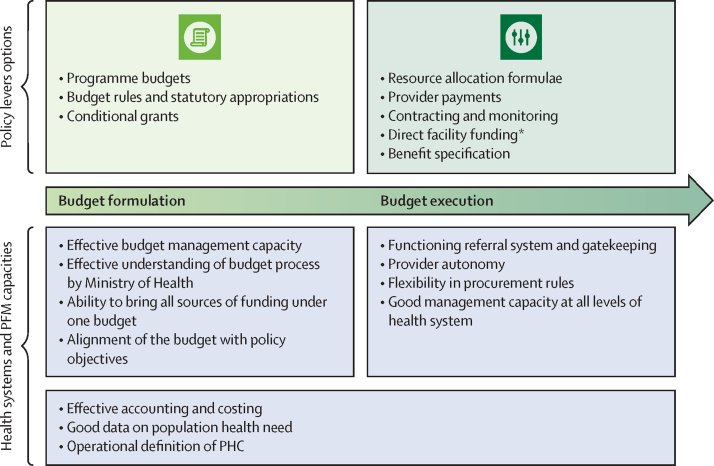
Health and public finance management system capacities needed to exercise public finance management policy levers PFM=public finance management. *Direct facility funding also requires individual facility bank accounts.

For any of these policy levers to be feasible, various health system and financial capacities need to be strengthened as well, such as budget management capacity at the Ministry of Health, high-quality data on health status and needs, and effective accounting practices. Figure 10 sets out these capacities.

Decentralised systems are closer to populations than centralised ones and have the potential to provide more flexible and responsive PHC services. However, they are at higher risk of local management problems and inequities. It can also be more difficult to influence resource allocation in decentralised systems.

Regardless of the type of system in any country, strong monitoring, performance management, and enforcement of appropriate budget and public finance management systems to ensure that resources reach PHC are as important as the initial process of resource allocation.

The Commission recognises that it is often difficult to make major or rapid shifts in allocation of resources in existing systems. In most cases, the discussion must be about incrementally influencing spending. However, as the COVID-19 pandemic has shown, sometimes major disruptions can create space for major reforms (and across multiple sectors) if appropriate policies have been identified in advance. Ultimately, these are complex processes that pose significant implementation challenges. Further, they rely on having data on variables such as costs and activities, together with numerous other supporting functions and systems. Ultimately, all of these systems are driven by human behaviour. Thus the following section addresses a key feature of every complex system for financing PHC: how it organizes the incentives for people to deliver and access high quality people-centred PHC.

## Section 5: Getting incentives right for PHC

Provider payment and incentives are another tool to ensure resources reach frontline providers and are used most efficiently. Countries can do more to create incentives that direct the behaviour of organisations that provide PHC and users towards people-centred PHC. In this section, we propose our vision of the PHC payment system based on a concrete set of principles. We describe the pathway countries can follow to make progress towards this vision and lay out the basic functions that need to be strengthened along the way. We also consider what motivates individual health workers, including the need to foster a culture of professionalism. Finally, we examine the role of provider payment policy in reducing financial barriers for those in need of PHC. Key messages from Section 5 are presented in panel 12.

Panel 12 **Getting incentives right for primary health care (PHC)–key messages**

• Incentive policies for providers and users are inextricably intertwined: PHC provider payment policies are integral to the elimination of user fees and informal payments for PHC services.
• Incentives alone cannot solve all PHC financing problems, but they should at least not work against PHC service delivery objectives.
• The Commission's vision of how PHC provider organisations should be paid is a context-specific blended payment model with capitation at its centre because that is most aligned with the principles and objectives of PHC.
• The blended payment model purposively combines capitation with elements of other payment methods (such as fee-for-service or performance-based bonuses for selected high priority services, and budgets to cover unavoidable fixed costs) to maximise beneficial incentives and offsets perverse incentives of each payment method, while ensuring other service delivery objectives, such as access, are met.
• Countries should only embark on provider payment reform when they are ready. The transformation of the PHC provider payment system is a complex process with distinct political economy challenges. The aim is to make incremental progress that involves continually strengthening supporting systems as the payment model evolves.

### The need to get incentives right

An incentive is an economic signal that directs individual health workers, health provider organisations, and patients towards self-interested behaviour. We know that incentives influence the performance of PHC providers and the behaviour of users.^151^ However, getting incentives right is not a panacea. As noted in section 4, the key problem in many LMICs is that insufficient resources actually reach PHC providers. No amount of tweaking incentives will help when newly qualified professionals often look to establish lucrative specialist practices and facilities are poorly equipped, making it difficult to staff primary care clinics. Dual practice, widely observed in LMICs, is a symptom of precisely this problem.^152^ To deliver high-quality PHC, it is essential that doctors, nurses, and other cadres of staff are valued, with adequate remuneration and conditions of work to attract them into PHC as a long-term career choice.^96^, ^153^

In many countries, the way health-care providers are paid often works against the objectives of PHC. In systems that pay providers a fee for a service, they typically set higher payment rates for specialty services, giving providers a financial incentive to prioritise curative, rather than preventive, care. In many LMICs, people lack trust in PHC and choose to seek care at higher levels, even if they have to pay more. Addressing these problems is especially difficult when funding and provider payment systems for PHC are fragmented. Case studies from LMICs have documented that the typical PHC provider receives funding from multiple payers using different payment systems for different population groups.^154^ In addition to creating administrative hurdles for PHC providers, when payments are poorly coordinated, the incentives generated might not align well (or might even conflict) with the objectives of PHC. These incentives can instead drive providers to prioritise certain patient groups in ways that exacerbate inequities or health services that are of low value to patients but lucrative for providers. Some of these problems are evident in China, as described in panel 13.

Panel 13 **China's experience with fragmented payment structures for primary health care (PHC)**

The Commission's China case study focused on the fragmentation of PHC financing.^145^ In China, PHC is mainly provided at village clinics and township health centres in rural areas and at community health centres and stations in urban areas. Financing for these institutions comes mainly from two sources: social health insurance and the essential public health fund. Social health insurance pays for a medical care package largely using fee-for-service payment, whereas the essential public health fund pays for a package of public health services using a population-based method (capitation). These two sources of funding are managed by different government authorities at both the national and local levels: social health insurance is managed by the Department of Social Medical Security, whereas the essential public health fund is managed by the Department of Health.

This fragmented payment system has been a barrier to integrated PHC for several reasons. First, a lack of coordination between the two funds' administrative authorities has resulted in separate delivery of medical and public health services. Second, fragmented funding can make it difficult for different cadres of PHC providers to coordinate their services, even when they are working within the same institution. For payment purposes, PHC providers try to maintain clear boundaries between the medical and public health services they offer; this is difficult, particularly in the case of services for non-communicable diseases. For example, payment incentives might lead doctors to focus only on providing medical services, even if they should also be playing important roles in disease prevention and public health services. Third, separate information systems have been established for the medical and public health services, making it difficult to manage the health of individuals and communities holistically. Finally, the performance of various PHC providers is evaluated separately by social health insurance and the essential public health fund, impeding the health system's ability to determine whether it is achieving its objectives. The existing incentives are not aligned to encourage medical and public health providers to coordinate, or even share information, with each other, although they are serving the same community members. A number of counties in China have recently begun to experiment with changing this fragmented financing situation by pooling the two sources of funding for PHC to pay family doctor teams. The intention of this innovation is to encourage greater continuity and integration in PHC.

Chronic underfunding of PHC, fragmented revenue streams, and misaligned provider incentives all contribute to the fundamental problem mentioned in sections 2 and 3: that many users in LMICs pay out-of-pocket fees for PHC services, which act as a barrier (disincentive) to accessing PHC, particularly for the poorest; for those who do choose to pay, user fees can lead to financial hardship.^74^, ^75^

Each option for paying PHC providers generates certain incentives that have been described in the literature.^155^, ^156^, ^157^ The key insight from the empirical evidence on the effectiveness of different payment methods is that no single payment method is perfect. Each payment method carries advantages and disadvantages.^158^, ^159^, ^160^, ^161^ Many countries have therefore moved towards a blended payment system, which combines elements of multiple payment methods, in part to maximise the beneficial incentives and minimise the perverse incentives of each option. We describe the main categories of payment method used for PHC.

• Tying payments to inputs, as with a line-item or global budget, is a passive form of purchasing. It provides a facility and its staff with a stable income, which is especially important in hard-to-serve areas, and contains costs. However, this provider payment method generates no strong incentives for providers to address the health needs of the population in the catchment area. Input-based budgets are often rigid, so providers cannot easily move funds across budget lines to respond to local needs (eg, they cannot choose to cut utility costs to spend more on medicines).
• Paying for services, typified by a fee-for-service system (not to be confused with user fees) in which the provider receives a payment from the institutional payer for each service provided, prioritises meeting users' demands. However, it carries many disadvantages, including an incentive to provide more care than is needed (particularly services with higher fees) and rarely prioritises preventive care. Pay-for-performance is a common add-on to other payment methods, whose purpose is to incentivise high-quality care through bonuses for reaching service coverage or quality targets, but can in principle result in gaming and the neglect of aspects of care that are not being measured.^162^, ^163^
• Population-based payment, which in this Commission we refer to as capitation, gives providers a fixed per-person payment, determined and paid in advance, to deliver a defined set of services to each enrolled individual for a specified period of time. Under capitation, continuity of care, a key prerequisite for successful PHC, is built into the reimbursement mechanism. Providers have an incentive to attract more patients to their practices and contain costs. However, a provider's revenue might not be enough to cover the costs of serving the population if some groups have higher needs than anticipated by the payment formula or if payment rates are set too low. In this case, there might be an incentive to avoid enrolling higher-need individuals, refer patients unnecessarily to specialists for care that could be provided in primary care, and skimp on quality of care.

The payment system itself is not the only force creating incentives for providers. Broader purchasing arrangements, including contracting, monitoring of provider performance, and population enrolment, can also generate both financial and non-financial incentives. For example, conditions of contracting, such as accreditation status or data reporting requirements, might create incentives for providers to improve their quality standards and upgrade information systems. Contracting arrangements might also specify service delivery standards, often tied to national clinical guidelines; this creates additional financial incentives if those standards must be met for providers to be paid.

### The Commission's vision of a people-centred payment model for PHC

#### Blended payment with capitation at its centre

The Commission's vision for PHC provider payment is a context-specific blended payment model built on capitation. This structure embodies principles that the Commission argues should form the core of PHC. Payment systems should allow adequate resources to flow to the PHC level in ways that: are equitable; match resources to population health needs; create the right incentive environment to promote the full PHC spectrum of prevention, health promotion, and management and treatment; foster people-centeredness, continuity and quality of PHC; and are flexible enough to support changes in service delivery models and approaches.

Capitation is a prospective population payment system.^164^ Because it is not tied to specific inputs or the volume of services delivered, capitation payment gives providers flexibility to coordinate and optimally manage care for individuals and populations. It is the only payment method that is based on the principle of equity, as its starting point is an equal fixed payment per person, which can then be adjusted based on health needs or other factors. Capitation payment is the only method that pays PHC providers for managing population health and prioritises preservation of good health, rather than delivering individual services to address health problems. As a prepayment-based system, capitation also provides a predictable and stable revenue stream to PHC providers that can be used to flexibly deliver services in responsive ways.^156^, ^165^

As PHC service delivery models become more community-based, patient-driven, and technology-enabled, payment methods need to be flexible enough to adapt to more varied, complex, and dynamic service delivery. Payment should compensate providers for delivering all services specified in a PHC package, some of which might not appear in typical fee-for-service lists or are not delivered in facility-based settings (such as essential public health functions, telemedicine, care management, or patient engagement). Capitation payment is flexible and can be redirected quickly in support of the service delivery model, as under this model providers have a large degree of financial and managerial autonomy.

If capitation has so many benefits, why does the Commission recommend a blended payment model? As noted above, capitation payment also has some clear drawbacks, such as encouraging underprovision, selection bias towards low-need patients, and unnecessary referrals to other levels of care.^157^ Blended payment models bring the benefits of capitation as the starting point and then use elements of other payment mechanisms to deliberately offset capitation's disadvantages and support achieving other specific health system objectives.^157^, ^166^

Blended payment models for PHC typically include a budget payment to cover unavoidable fixed costs, particularly in low-population or hard-to-serve areas; some fee-for-service carve-outs for health conditions or services that are high priority or at higher risk of being underprovided in capitation; and, in some cases, performance-based payment to incentivise reaching coverage targets for priority services and improving quality of care. Other complexities may be added to align with evolving and innovative service delivery models (panel 14).

Panel 14 **Paying for integrated care**

Differences in health financing arrangements, including payment methods, are frequently cited as a major barrier to more integrated approaches of service delivery. Successful integration requires sustained investment in staff and support systems, funding for start-up costs, and flexibility to respond to needs that emerge during implementation.^167^ A review of the evidence (mostly from high-income countries) found that a range of mechanisms have been used, often in combination, to achieve better service integration.^54^ This includes the commitment of dedicated resources to support the development of innovative care models, such as through targeted payments to finance infrastructure for provider networks, or the use of start-up grants to promote care coordination and integration activities.^168^ Countries are also increasingly experimenting with what has been referred to as value-based payments, which seek to link provider payment to a predefined set of evidence-based clinical process or outcome measures.^166^ Examples of value-based payment include bundled payments, shared savings, and global budgets.

Bundled and global payments are disbursed as a single payment in form of a lump sum per period for a specified population (global payment) or per episode or condition per patient (bundled payment) to a collective of providers. By linking payment to clinical, process, and outcome measures, providers are incentivised to increase efforts to improve patient care and process efficiency. As the payment is transferred as a single lump sum, regardless of the number of services provided, value-based payments are expected to promote care coordination and integration across providers and so reduce wasteful duplication of services and unnecessary hospital use. The Netherlands and various states in the USA have introduced disease-based bundled payment schemes for mostly chronic conditions, such as type 2 diabetes or cardiovascular disease.^169^, ^170^ These involve reimbursing providers for a package of services on a predefined patient pathway per patient and for periods of up to 1 year. Global payment models include shared savings programmes and comprehensive care payments. Shared savings programmes essentially mean that the payer and providers share the risk of rising expenditure, that is, providers that successfully lower their growth in health-care costs while continuing to meet quality standards will be able to keep the savings and reinvest them. Examples include the Medicare Shared Savings Program in the USA and the Healthy Kinzigtal integrated care programme in Germany.^171^ Global payment models involve fixed payments for the care of a patient during a specified time period. Several countries have additionally introduced pay-for-improvement, pay-for-coordination, or pay-for-performance schemes in primary care, incentivising chronic and coordinated care in particular, although the evidence of their benefits remains mixed.

Finally, a number of countries have experimented with different financing mechanisms, such as shifting responsibility for funding of particular components of service delivery between funding agencies.^172^ Others have introduced pooled funds to integrate health and social care or structurally integrated budgets, in which responsibilities for health and social care are combined within a single body under single management, such as within the Integrated Health and Social Care Board in Northern Ireland.

The evidence of what works remains patchy. However, an important lesson is that “integration costs before it pays”.^167^ Indeed, evaluations of novel schemes often find an increase in cost, mainly because the new service delivery model uncovers unmet need.^173^ The creation of new coordinating mechanisms will not compensate for lack of resources. The injection of one-off extra funding to pay for new services will not necessarily ensure long-term sustainability, particularly where new approaches fail to be incorporated into routine care.

Pay-for-performance has received considerable attention from policy makers and researchers in the last two decades.^174^, ^175^, ^176^ Explicit performance incentives encourage providers to focus on aspects of PHC that are unlikely to be incentivised by the global base payment and might be prone to quality skimping or underprovision. However, the current evidence suggests that improvements that result from pay-for-performance schemes are often less than anticipated.^174^ Financial incentives should be relatively low powered to prevent disproportionate focus on rewarded tasks and to ensure sustainability.^177^, ^178^, ^179^, ^180^

Performance monitoring should happen alongside implementation of the blended payment model. Under capitation, the payment is divorced from activity, meaning a concerted effort needs to be made to monitor how well health-care providers are doing. Indeed, a key advantage of pay-for-performance is that it can contribute to better accountability, such as improved measurement of provider activity and performance, and a more informed dialogue between purchasers and providers.^181^

#### Payment levels and flows

For a blended provider payment system to create meaningful incentives that affect providers' behaviours, adequate funding needs to flow through funding streams and not create conflicting incentives. Providers must also have the autonomy to manage funds and respond to incentives. Capitation payment rates on which a blended system is based should reflect adequate funding levels to purchase the inputs needed to deliver the package of PHC services according to quality standards laid out in national treatment guidelines. At the same time, payment levels must also align with the resources available from pooled public sources and the political priority placed on PHC. As funding to the health system overall grows, and as the skills of health workers to deliver the package of PHC services increase, capitated rates can be increased to channel a larger share of overall funding to PHC.

Capitation payments should be managed at the lowest level where they can be used effectively to provide the full range of services to address population health needs. In some systems, such as in Estonia^182^ and England, this is a frontline PHC provider organisation. Other countries, including Ghana and Kenya, are experimenting with establishing groups or networks of PHC providers to manage capitation payments. These platforms can share some functions, such as information management and quality assurance, and close capacity gaps. Providing capitation payments to a multidisciplinary provider group has been shown to foster coordination across the continuum of care.^166^ At a higher level still, in Brazil and Chile, local government authorities manage capitated funds for the delivery of PHC, and individual health workers can receive bonuses when their team or facility achieves performance targets.^55^, ^107^ In Thailand, capitation payments go to a contracting unit for primary care. More than likely, funds will need to be directed to multiple levels simultaneously to enable the provision of both individual-focused care and population-level services including essential public health functions (see section 4).

Funding flows from multiple sources need to be harmonised to align the incentives for providers. Although it is usually not feasible (or necessarily desirable) to merge all funding flows, a coordinated and deliberate payment system can help to achieve greater coherence at the provider level. A good example is Tanzania's system for providing direct facility financing using health-sector basket (pooled) funding from donors who have agreed to finance the health sector budget through the central treasury.^141^

#### Interface with payment at other levels of care

The way outpatient specialty and inpatient services are paid for can also influence the overall incentive environment for PHC providers. For example, if PHC providers are paid a fixed capitation payment, but hospitals are paid based on the volume of services provided, the potential adverse incentive of capitation payment to increase referrals is reinforced by the adverse incentive for hospitals to increase the volume of care, including admissions.^55^, ^165^ As mentioned in section 4, additional policy measures might therefore be needed to harmonise incentives across the levels of care, including gatekeeping requirements to enforce referral guidelines, ring-fencing the payment pool for PHC, or introducing payment caps at the level of the hospital to reduce incentives for unnecessary admissions.^183^

### Progressing towards a blended capitation-based payment model

An effective capitation-based blended payment is a sophisticated provider payment model that relies on a complex set of policies, implementation arrangements, and purchaser and provider management capacities. Reaching this stage requires a clear vision backed by strong political commitment, significant time, and consistent investment. As with the introduction of any new provider payment system, the responses of providers to the rollout of a capitation-based blended payment model cannot be fully anticipated. However, their responses are likely to be different if the payment model is evolving from an input-based budget (in which providers have little autonomy and might welcome the flexibility of capitation) or from fee-for-service payment (in which providers have more control over their revenue and might resist the move to capitation).

Moving towards a blended payment model, as with any reform process, requires anticipation and deft management of complex political economies, collection and analysis of data to address emerging issues, and flexibility to address unintended consequences in a timely manner. This process can seem dauntingly complex—however, the alternative is to remain with a status quo that is failing to provide the incentive environment required for delivery of PHC to improve health outcomes and equity. Provider payment reform is incremental and rarely is there a perfect time to start.

#### Evolution of the payment system

Many payment systems have evolved to reach blended payment models in similar ways, regardless of their starting points. In most countries, the introduction of equity-orientated and efficiency-oriented payment system reforms starts with a basic population-based capitation model. Typically, these systems are transparent, involve simple per-capita payments, and are easy to administer in places where data automation is limited. Most payment systems then eventually introduce risk adjustments. Complexity continues to increase over time as additional payment methods are added.

**Figure 11:**
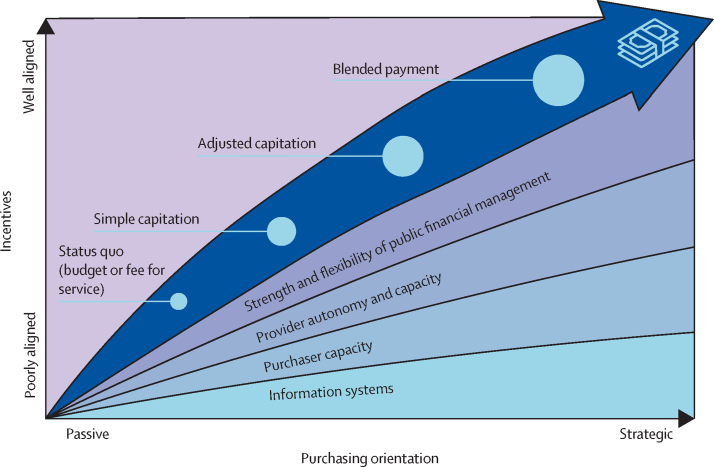
Strategic pathway for moving to a blended, capitation-based payment

Figure 11 presents the pathway of how countries can pursue the Commission's vision of a blended payment system, showing the interim steps in the evolution. Figure 11 also indicates the basic functions that should be strengthened over time to support payment reforms. Learning from other countries' experiences can help countries committed to progressive policies to hasten the development of their own context-appropriate payment models.

The trajectory towards a population-based payment model involves several concrete steps. First, establish a baseline capitation payment system. For capitation to promote equity and create clear incentives, the payment amount should be based on a formula that links the payment parameters (base per-capita rate, number of enrollees linked to the provider, and any individual or provider-level adjustments) to a defined package of PHC services. Each payment parameter can range from simple to complex. They function as strategic levers to maximise the potential benefits of the payment system while minimising adverse incentives and unintended consequences.

Second, define the PHC package. As mentioned in section 1, defining a package of PHC services linked to capitation payment creates an opportunity for each country to clarify what its definition of PHC includes (as well as setting boundaries between primary care, outpatient specialty services, and secondary care). Specifying what is included in the PHC package and where it is provided can drive shifts in service delivery priorities and promote the integration of vertical programmes into PHC.^155^ The PHC package linked to capitation (which may be a subset of the broader PHC package) can also be expanded to serve as the platform for financing essential medicines,^155^ either directly or through an outpatient drug reimbursement scheme, because medicines are a key driver of out-of-pocket expenses in LMICs.

Third, manage enrolment. A capitation payment system relies on all individuals being enrolled (registered) with a given provider for a fixed period. The assignment of a fixed and defined population to a single PHC provider is an advantage of the system. PHC services contribute to improving the health of communities by organising around populations rather than only serving individuals who actively seek health care. Individuals can be enrolled with providers through administrative assignment (as defined by a geographical catchment area) or by their own choice (known as open enrolment). Open enrolment during select time periods allows financing to support users' choices; in principle, this creates incentives for providers to be responsive to patients and provide high-quality services.^155^, ^184^, ^185^

Fourth, adjust for risk levels. Risk adjustment is a correction tool that uses a measure of risk variation to compensate health providers appropriately for the expected costs of providing necessary services for their enrolled populations. The calculations account for variation in health need, typically by using data on different baseline characteristics such as levels of health, sex or gender, chronic disease risk, and socioeconomic status. Risk adjustment protects higher risk and sicker patients from the incentive providers have to avoid caring for them when their care is predicted to be especially costly. Other adjustments, such as for geographic area, might also be included if there are significant cost variations for delivering the same package of PHC services in different locations, such as in rural and remote areas where fixed costs for transportation or use might be higher.

**Figure 12:**
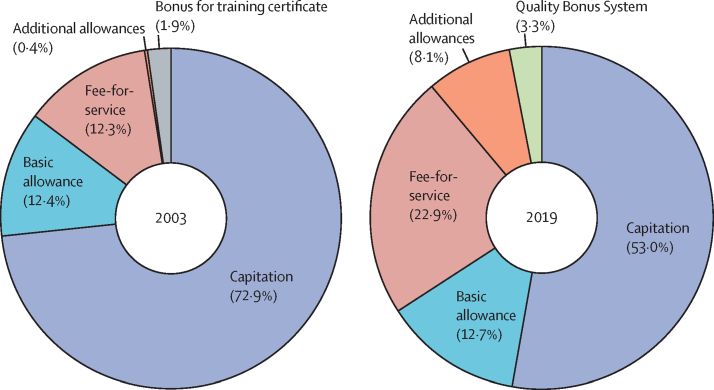
Estonia's blended PHC payment system in 2003 and 2019 PHC=primary health care.

Fifth, blend payment methods. Countries almost always find that the precise blend of payment changes as the system matures. Various factors, including history, culture, priorities within the PHC system, or shifting disease patterns in a country, can drive these changes.^186^ In Estonia, for example, the relative contribution or blend of different payment methods (capitation, fee-for-service, fixed basic allowance, and pay-for-performance) has evolved over time, as described in panel 15 and figure 12. In Aotearoa, New Zealand, similar evolutions are also occurring, as outlined in panel 16.

Panel 15 **Development of the primary health-care (PHC) payment system in Estonia**

The Commission's Estonia case study focused on the process through which the capitation-centred provider payment model for family doctors was developed after independence from the Soviet Union in 1991.^182^ Estonia is a high-income country with an ageing population of 1·3 million people. The country's health system has been lauded for achieving good health outcomes at low cost. Public funding represents the predominant source of health financing, constituting approximately three-quarters of total health expenditure.

The current system has been evolving over nearly three decades. In the late 1990s, Estonia undertook major payment reforms in parallel with organisational changes to the health system. Everyone in the population was registered with a PHC provider, either a family doctor, general internist, or paediatrician. Family doctors worked as private practitioners contracted by the national health insurance fund. The previous fee-for-service system was replaced with a capitation system, initially based on a flat per-person rate that was subsequently age adjusted. The capitation rate was intended to cover the salaries of a practice's family doctor and a nurse, as well as a defined set of equipment and certain laboratory tests. A basic allowance covered the costs of equipment, facilities, and transportation. Some fee-for-service payments were retained for a defined list of diagnostic tests and procedures. An additional lump sum was provided to cover the expenses of family doctors working in rural areas.

This new payment system was designed to incentivise family doctors to take more responsibility for diagnostics and treatment and provide continuity of care; the system also compensated doctors for the financial risks of caring for older people and working in more remote areas. Moving to capitation-based funding represented a major shift from the previous fee-for-service payment mechanism, in which doctors and health-care institutions were incentivised to perform a large number of diagnostic procedures. The shift in payment model was introduced along with a new organisational and contractual mechanism that has successfully promoted PHC while increasing the freedom and independence of PHC providers.

The payment system has continued to mature as new elements are added, as shown in figure 12. In the 2000s, a voluntary pay-for-performance element was added that was designed to motivate family doctors to widen their scope to include more prevention services (eg, childhood vaccinations) and chronic disease management (eg, hypertension care). This reform was widely accepted by providers: the proportion of family doctors participating in the scheme rose from 50% in 2006, to 97% in 2014. In 2015, participation in this quality-focused bonus scheme finally became obligatory for all family doctors, and individual performance results became public information. The basic allowances have also increased to cover rising costs of management and information systems and to motivate individual providers to form groups and expand the scope of services offered.

Panel 16 **Capitation in the reform of Aotearoa New Zealand's primary health-care (PHC) system**

The Commission's case study on Aotearoa (New Zealand) focused on capitation payment for general practitioners.^187^ Capitation was suggested as a way of paying PHC providers as far back as the 1930s, when the government embarked on a major reform to introduce free, integrated, comprehensive health care for the entire population. However, due to resistance from general practitioners, the capitation plan was scrapped and general practitioners remained as independent small businesses with their services funded by a fee-for-service government subsidy and out-of-pocket user fees. By the 1990s, the subsidy was funding only 20–30% of general practitioner costs, with the rest coming from user fees.

In the early 2000s, the government developed a Primary Health Care Strategy, the first major PHC policy since the 1930s. The government established a new type of not-for-profit entity, the Primary Health Organisation, which enlisted PHC providers on a voluntary basis. This allowed the health system to shift to universal weighted capitation at the Primary Health Organisation level. The shift ensured that all citizens could receive subsidised care in a way that accounted for need. The move to capitation was also designed to control government expenditure on PHC and expand the range of services that could be delivered by nurses. Over time, the Primary Health Care Strategy enabled the government to increase PHC funding and allocate a greater proportion to Primary Health Organisations working with higher-need populations. Large decreases in unmet need for general practitioner services were observed in the first 5 years, including for Māori people.

However, ongoing issues also persist. Although the funding was allocated using a risk-adjusted capitation formula, it insufficiently accounted for variation in needs related to ethnicity and deprivation. Despite a number of reviews, the formula has not been improved, in part because of concerns that further risk adjustment would create so-called winners and losers and undermine support amongst key groups. Instead, numerous ad-hoc changes have been made, resulting in complicated funding arrangements that can be confusing. There have also been concerns about continued user fees. Although they decreased initially, particularly for those on low incomes, the decreases were less than anticipated given the large increases in funding and the continued existence of charges might be blunting the provider incentives that were meant to be created by capitation. Major ongoing reforms to the structure and delivery of health services seek to address some of these challenges.^188^

#### Strengthening basic functions

LMICs considering shifting to a more strategic payment model for PHC need to consider when and how to embark on the process. The Commission emphasises that the right choices of when and how to transform the PHC payment model depend on the country context. Experience suggests that if a country is not ready (with conducive political will and some basic functions in place), payment reforms can be disruptive and potentially harmful.

In many LICs, the priority issue is the amount of coordinated funding reaching PHC provider level. These countries should focus first and foremost on getting more funds to PHC providers that can be used flexibly to meet population health needs. This might involve changes to the funding allocation mechanism, as discussed in section 4. In other countries, strengthening basic functions alongside an assessment of the current provider payment system is an appropriate first step.^156^

Even a perfectly-designed capitation-based blended payment system cannot work without basic supporting functions in place (figure 11). These will need to develop and evolve as the payment system becomes more sophisticated. It is possible that digital technologies can help to support the evolution of the provider payment system but this remains to be seen (appendix p 35). We describe the main basic supporting functions.

• Routine data capture and electronic record systems that are interoperable across datasets, ideally for the whole country, are needed to support complex payment systems. This is an iterative process: requiring data for payment leads to better data, which in turn allows for implementation of a more sophisticated payment system. The capacity to analyse and interpret data should be strengthened if policymakers are to act on data-driven evidence. Data are necessary for payment calculations and monitoring of population enrolment, population characteristics, and service delivery performance. The population enrolment list or database must be accurate because payments to providers under capitation are influenced by the number of individuals enrolled with that provider. The method of creating the list and giving providers access to it should be transparent so providers trust the list and their final payment amounts. Data are also necessary for population characteristics monitoring. To adjust population-based payment rates for need, detailed data on the key characteristics of the registered population are required. Last, data are necessary for service delivery performance monitoring because it is important to capture information on clinical care, health-care use, and patient experience to understand how well PHC providers are addressing population needs and to monitor undesirable behaviours, such as underprovision and excess referral. Performance monitoring should be aligned with the benefit package so that providers can be held to account on the services they deliver.
• Giving providers more autonomy to make decisions about how to provide PHC supports their flexibility to respond to incentives.^11^ Fostering provider autonomy includes the acquisition of new skills, many of which might be non-clinical—for example to do with coordination and communication. It also necessitates building their capacities to understand and manage incoming resources. In systems where providers have little management autonomy or do not have the skills to manage new procedures, the results of new purchasing and payment methods will be either be diminished or perverse. For example, in Indonesia, the purchaser for the national health insurance system pays PHC providers by capitation. However, there are strict rules about how public providers can allocate those funds among staff payments and other operational costs.^116^ In addition, providers that receive funds from multiple revenue streams must know how to allocate and account for them separately. Introducing complicated rules without training providers to understand them greatly diminishes the potential of capitation payment systems to encourage better and more efficient use of resources for service delivery.
• Public financial management systems must be flexible and straightforward. Many governments' public finance management systems are notorious for their rigidity regarding the use of funds and the complexity of their accounting and financial management requirements. Traditional public finance management systems might even prohibit prepayment to PHC providers, which is an essential design feature of capitation. However, when health facilities have strong financial management capacity and authority to make some financial management decisions, they are more likely to adjust service provision and deploy inputs based on the needs of the population.^115^ Payment system reform also requires simplification, by reducing administrative layers and burdens, and harmonising funding flows. This is key to ensuring that resources reach facilities on time and in full, and are appropriately tracked. Delays can be corrosive. In Ghana, for example, delays in fund transfers eroded trust in the capitation system.^189^ One potential solution is to establish facility bank accounts, which might require changing the legal status of facilities within the public finance management system, to enable direct payments to facilities,^128^ as has been tried in Tanzania,^190^ Uganda,^128^ and Nigeria.^128^
• The purchaser—whether it is the government or a social health insurance agency—should have the institutional authority and technical capability to enter into legally binding agreements with health-care providers that specify the characteristics and minimum requirements of contracted providers, services that providers will deliver, the methods and terms of payment, reporting requirements, and processes to resolve disputes.^156^

The experiences of countries that have attempted reforms of provider payment systems also highlight the need for PHC providers to be involved in the design of policies and adequately sensitised so that they understand the changes and lend their support to implementation.^191^, ^192^ Patients also need to be given information on their eligibility and entitlements. A method to monitor when providers incur excessive financial risks is required, and risk mitigation strategies considered. Finally, it is important to have a policy on the portability of benefits to determine how patients can access services when away from their registered facility and how their temporary providers will be paid.

#### Managing the politics of provider payment reform

Introducing a new provider payment system such as capitation is a highly technical endeavour. It is also a complex political process because making such major system-wide changes affects many of the stakeholders in the health system, including every PHC provider and patient served by the health system. It requires anticipating the effect of the new system on major stakeholders, including medical professionals (both general practitioners and specialists), social health insurance administrators, private sector providers, and the pharmaceutical industries. Introducing reforms requires significant interagency coordination within the government and possibly with donors.

The political economy challenges depend on a country's starting position. If the status quo is input-based budgets, a key stakeholder to engage is the ministry of finance because of its central role in defining the budget approach and public financial management rules. In countries with institutionalised fee-for-service, health providers might try to impede the reform, because population-based payment involves a transfer of risk to providers and is often perceived to be less lucrative for them as income is no longer tied to services.

The various possible pitfalls a country can encounter when attempting to change provider payment systems are evident in the experiences of several countries. The American Medical Association has opposed capitation (amongst other reforms) since the 1930s, when primary care physicians created a new insurance company to prevent hospital insurance plans from entering the primary care sector and influencing control over fees.^193^, ^194^ Medical associations in South Korea (where all physicians are paid on a fee-for-service basis) have complained about price regulation by the government and successfully pushed back on cost control reforms, such as diagnostic related groups and global budgets.^195^, ^196^ Providers' resistance has been influential in thwarting or undermining reforms in Taiwan,^195^ USA,^193^ Turkey,^197^ and Ghana (panel 17). Patient pushback (often fuelled by provider activism) against limits to provider choice under capitation has been documented in some of these countries.

Panel 17 **Lessons learnt from the capitation pilot in Ghana**

The Commission's Ghana case study examined a pilot of a capitation scheme in one region of the country between 2012 and 2016.^189^ The national health insurance scheme was introduced in Ghana in 2003, to replace the so-called cash and carry system based on user fees that had prevailed in the country after a free health care for all policy was abolished in 1969. The national health insurance scheme started with a fee-for-service payment for all covered services including primary health care (PHC), but that resulted in cost escalations that threatened the sustainability of the scheme. There was, for example, a large increase in annual spending, in the first 5 years of the scheme, driven by increases in the number of claims per insured member.^198^, ^199^ In response, the national health insurance authority introduced diagnostic related groups for outpatient and inpatient services and itemised medicine fees in 2008. However, even though the number of claims per member was reduced by 13%, overall costs kept rising and the complicated claims management process led to delays in processing claims.

In 2012, the national health insurance authority piloted a capitation payment system for PHC in the Ashanti region. However, the pilot was suspended after 5 years and various challenges. Agitations and protests by providers had begun at the start of the reform.^189^ Their objections were especially consequential because 2012 was an election year and the government was highly sensitive to any social unrest. In response to the providers' agitation, the Ministry of Health and the national health insurance authority made major policy compromises, including a reduction in the package of services and a 22% increase in the per capita rate.^189^ These and other compromises could possibly have been avoided if the implementers had considered various political, social, and economic factors from the outset. For example, they might have faced less opposition if they had chosen a pilot site that was less politically sensitive and not dominated by politically powerful private health care providers.

Many lessons were learned from the pilot about stakeholder engagement and building trust among providers and users. It also showed the importance of the supporting systems needed to implement capitation payment, which continues to be among policy options considered by the national health insurance for future reforms. The experience of the capitation pilot has also triggered discussions about service delivery reforms to form PHC networks to close gaps in provider clinical capacity, which also posed a challenge to the successful implementation of capitation payment.

In addition to the wider context, specific design characteristics of the proposed changes can also hamper or foster reform efforts. In HICs, capitation has often been implemented as part of a larger health reform, such as a shift towards family medicine.^200^, ^201^, ^202^ This was the case in Estonia, where the early establishment of a strong association of family doctors helped build support for PHC reform. In LMICs, capitation has more often been introduced as part of financing, rather than delivery, reforms. Several countries have combined the introduction of a capitation-based system with the creation of national health insurance programmes; examples include Indonesia,^203^ Thailand,^204^ and Kyrgyzstan.^205^

Changing the provider payment system also requires engaging with health system users. Many will have concerns, such as about whether the new system would limit their choices or introduce new forms of gatekeeping. However, one of the key potential benefits of capitation is fostering longer-term relationships between health system users and PHC providers; in principle this can bring more personalised care management and attention to prevention.^155^ Maximising this benefit and minimising perceived limits on choice require concerted patient education efforts and facilitating informed choice.

As is discussed further in section 6, advocates for changing the provider payment system can define political strategies to strengthen support and neutralise opposition to the proposed reforms.^197^, ^206^

### Motivating health workers

Thus far, our discussion of incentives has been focused on PHC provider organisations. Health care is delivered by individuals or teams of health workers; therefore, getting individual incentives right means health workers will be motivated to provide quality care and refrain from engaging in dual practice. Because community health workers have a vital role in the PHC system of many countries, panel 18 addresses the question of how this cadre of health worker should be paid.

Panel 18 **Paying community health workers**

WHO recommends that community health workers should be remunerated for their work “with a financial package commensurate to the job demands, complexity, number of hours, training, and roles that they undertake”.^207^ This of course leaves open the question of how community health workers should be paid. Before addressing this question, it is important to highlight that community health workers in many low-income to middle-income countries feel they are underpaid and poorly compensated for their time and effort, and their pay is often delayed.^208^

Most community health worker programmes offer some kind of financial incentive, with the choice between salary, monthly payments, or performance-based payments.^209^ Monthly payments are often a way of avoiding giving further benefits associated with salaried government employees. It is notable that Ethiopia, Ghana, Malawi, and Nigeria have large programmes in which community health workers have the status of formal civil servants. Whether community health workers should be paid by salary or performance-based financial incentives must consider more than effectiveness. However, even if we focus on the narrow question of effectiveness, we are unaware of any studies that have compared the performance of community health workers under these two alternative ways of paying.

By contrast, there is a growing body of evidence that suggests some types of performance-based financial incentives are more effective than others in improving delivery outcomes of community health-worker services.^210^ Large payments appear to be more effective than small ones, but they tend to shift effort away from non-incentivised activities. Giving performance payments to both community health workers and supervisors, and combining incentives with information to the community have been shown to be effective. Financial incentives have not worked when community health workers had limited control over the incentivised task, there were complex rules around disbursement of the incentives, and the focus was on selling products to poor households. Relatedly, there is good evidence that non-financial incentives (social recognition, trust, respect, and opportunities for growth and career advancement) can improve community health workers' performance and reduce attrition.^211^, ^212^, ^213^, ^214^

Financial incentives for community health workers need to be approached with caution, particularly the exclusive use of them. Although they can be effective, there is also scope for unintended consequences. Such a conclusion is reflected in WHO's suggestion that community health workers should not be paid “exclusively or predominantly according to performance-based incentives”.^215^

One frequently overlooked observation is that financial incentives generated by the provider payment mechanism at the organisation level might not be passed on to individuals. It depends on how individual health workers are paid. For example, pay-for-performance can provide a strong incentive at the provider level, but if most of the health workers are paid a flat salary, the effect on individual motivation might be less than expected. In low-income countries, salary levels are clearly important for health worker motivation but as influential perhaps are the delays in the monthly payment of salaries that are commonplace.

Because of the constraints of any payment system, the way in which services are provided depends to a large degree on the training and professionalism of the clinical workforce. It is essential that payment systems support and reinforce the professionalism of staff and their commitment to providing high-quality care. If the design of payment systems undermines professionalism by, for example, incentivising overprovision or discouraging continuity of care, staff can become demotivated.^216^ This is one reason why representative clinicians should be involved in the design of payment systems.

Health workers place great stock in their strong culture of professionalism, often supported and cultivated during training.^217^ This culture includes an orientation to the needs of patients, periodic self-reflection, and peer review. This relates to the importance of preserving provider autonomy. Similarly, there are direct non-financial incentives that can improve quality of care, such as opportunities for providers to share data on their performance with other professionals or with the public,^218^ and social recognition.^212^ Health workers appreciate having data that validates their perceptions that they are doing a good job; comparing them with their peers can be a powerful incentive to improve their practices.

### Addressing out-of-pocket payments for PHC

#### The role of provider payment policy

Progressive universalism, in which pooled funds should first be used to cover PHC to reduce out-of-pocket payments and replace the lost financing, requires action across all the health financing functions. In particular, removing financial barriers for PHC involves more than just changing user fees policy. It means ensuring patients do not face informal fees and are not sent to pharmacies to purchase medicines because public health providers are under-resourced. Provider payment rates and health worker salaries must be high enough to eliminate the need for user fees and informal payments. In this sense, provider payment policy is integral to the elimination of user fees and informal payments for PHC. In some countries, where escalating health expenditure from excessive use of services is a key concern, there might be a role for cost sharing but its effect on people with the lowest socioeconomic status should be carefully considered and mitigating measures put in place.

#### Incentivising the users of PHC

A key benefit of reducing out-of-pocket payments for PHC is to incentivise greater use of cost-effective health interventions, particularly amongst those with the greatest health needs. However, in some contexts, removing user fees is not sufficient to meaningfully expand financial access to care. Various programmes in LMICs have therefore introduced additional financial incentives for patients to use highly effective PHC services, such as immunisations and antenatal care. There is good evidence that offering cash or other financial incentives increases use of primary care services—although whether use translates into improved health outcomes is less clear.^219^, ^220^ Incentivising use of PHC will be more effective when the services are more readily available and of high quality. If the policy objective is to increase use, demand-side financial incentives should be considered before changes to provider incentives. However, there is little point in increasing demand for PHC if the available services are not ready or of sufficient quality to meet the population's needs.

### Conclusion

This section has presented the Commission's view that provider payment for PHC should be based on a blended payment model, adapted to each context but with capitation at its centre. It has presented a strategic pathway for countries to progress towards this model, recognising that each country has its own unique starting point. It has recognised that incentive policies for providers and users are linked, and the crucial importance of simultaneously moving towards an elimination of user fees and informal payments for PHC. Progressing this vision requires mobilisation of additional resources for health, allocating these resources to PHC, and ensuring that they work their way through the public finance management system to reach frontline service providers. The success of this technical strategy depends on a political strategy to set the vision, understand the interests of different stakeholders, and actively manage these interests.

## Section 6: The political economy of financing PHC

Transforming financing to support efficient and equitable PHC is often approached as a technical problem. Political and socioeconomic factors affecting financing reforms are frequently described as bottlenecks, barriers, or contextual factors. However, as has been highlighted throughout the report, the Commission recognises that these political economy elements are in fact central to any effort to understand, improve, or reform PHC financing.

In this section, we make the case that political economy analysis must be undertaken in conjunction with technical analysis and strategising for PHC financing reform, as part of a national mapping of the PHC financing ecosystem (section 7). This integration is an important first step in resolving some fundamental questions, including: if PHC is the best approach to achieve UHC, why is it not systematically and adequately prioritised in national budgets? And why are purchasing and other health financing reforms successfully implemented in some countries, and resisted in others?

### The Commission's vision of political economy

At its core, political economy brings together systematic explorations of politics and economics and power dynamics between stakeholder groups. Different schools of thought have focused on the so-called economy of politics, the economic constraints influencing political decisions, or understanding how the political context shapes the implementation of economic policy. The materialist approach, for example, focuses on the material conditions of a society's mode of production and argues that these determine how politics, economics, and social processes evolve.^221^ The new political economy approach states that policies can be analysed through the prism of neoclassical economics.^222^ Other scholars take an actor-based approach, seeking to identify the winners and losers from policy processes by studying incentive structures influenced by economic interests. The laws and political conditions that shape the material world change over time, thus political economy is essentially an historical science.^223^

Drawing on these traditions, political economy analysis in the health policy field has tended to focus on politics,^224^, ^225^ particularly on power relations among different interest groups and their relative abilities to influence reforms.^225^, ^226^, ^227^ Political economy analysis in health has also tended to analyse the outcomes of processes at a point in time and with a focus on a particular policy or specific issue, typically examining either the contestation and coalitions among interested parties that drive health system operations and reforms,^226^ or the nature and strength of political institutions that could stop the legislative process that underpins the enactment of reform in political decision-making.^105^, ^228^ Some authors have argued that the focus on political dynamics is too narrow^229^ and that understanding the roles of individuals within political structures has frequently been overemphasised.

Political economy analysis is also useful as a broader frame that examines the context, structures, and relationships that generate systemic features; what Jeremy Shiffman, a political scientist, focusing on the politics and global health governance of health policy-making in low-income countries calls the “enduring political and social arrangements not easily altered by the actions of individuals”.^230^

In this section, the Commission takes a relational view of political economy analysis. Our approach focuses on identifying how key actors—individuals, social groups, organisations, governments, and other stakeholders—relate to each other over time in determining access to, and distribution of power and resources, and how economic and social factors structurally influence these relationships. Applying political economy analysis to PHC financing helps to understand why resources for health are raised and allocated in particular ways to PHC for example, and what competition and contestation occurs throughout these processes. We conclude that political economy analysis has practical value in seeking to explain why efforts to improve efficiency and equity of PHC financing reforms have faced challenges, and to identify prospectively feasible strategies for particular political and socioeconomic contexts. Key messages from Section 6 are presented in panel 19.

Panel 19 **Political economy of primary health care (PHC) financing–key messages**

• Political, sociocultural, and economic conditions are as important as technical elements in the design and implementation of efficient and equitable financing for PHC. These political economy factors represent both constraints and opportunities.
• Advancing financing for PHC that is people centred relies on politically informed technical strategies, meaning that policy making in PHC financing and reform must be underpinned by political economy analysis.
• Prioritising PHC is possible at every income level and in any type of political governance model, given the presence of effective political alliances and socioeconomic conditions.
• Designing politically informed technical strategies requires navigating the evolving political economy context. Political economy analysis can inform the adjustment of the technical strategies to identify the pathways and challenges to the proposed change, taking the long view, and identifying the structural social or economic so-called red lines to be worked around.
• Developing PHC financing policy (whether incremental adaptations or a substantial transformation) and ensuring strategic investment in PHC require coherent policy aligned with the interests of key actors through collaboration and building coalitions among stakeholders (leaders, politicians, clinicians, technocrats, donors, and civil society representatives) and across sectors. This development might require achieving consensus or strategic compromise on how to expand access to PHC.
• Having a clearly articulated long-term vision is essential for making progress towards efficient and equitable PHC financing. Consistency, adaptation and staying on course are required when countries pursue long-term reforms, while retaining flexibility to take advantage of opportunities for change created by political and socioeconomic events such as political transitions and shocks, or emerging alliances.
• Sequencing is key. Planners must have the technical fundamentals and strategies ready in anticipation of windows of opportunity, which arise as a result of political dynamics and social and economic forces.

### A pragmatic approach to managing political economy considerations

The Commission builds on the application of political economy analysis to health financing by explicitly considering broader social, political, and economic features of a context that can influence the success or failure of PHC financing functions and reform efforts, as well as their evolution.

**Figure 13:**
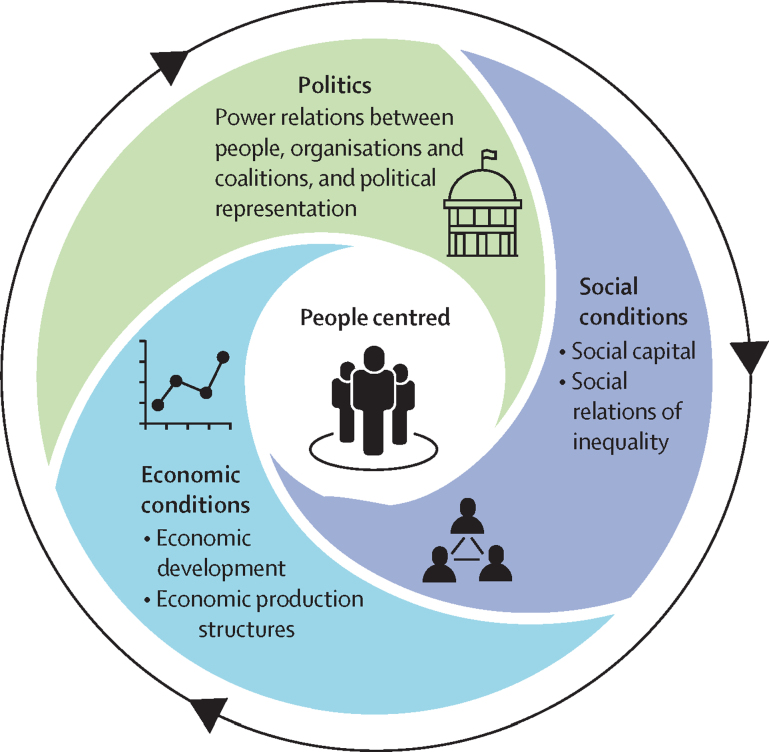
A political economy analysis conceptual framework for health financing reform

Our political economy analysis framework, shown in figure 13, takes into consideration three domains that influence financing for PHC. First, politics: including the range of actors (individuals, formal and informal organisations, and institutions) and their respective power, their relationships and contracts, their legitimacy, as well as contestation leading to the enactment of policies. Second, social conditions: encompassing social values, informal networks, class, caste, or other social constructs that can influence, for example, the options for distribution or redistribution of resources, including acceptance of, or resistance to, reforms such as greater pooling of resources. Third, economic conditions: including a country's level of economic development, production structures, levels of taxation and levels of aid, that facilitate or hamper the mobilisation of resources to PHC.

These domains are interdependent. Further, they are characterised by dynamic structures and processes that evolve over time—sometimes gradually and in other cases rapidly. Each is discussed in more detail in relation to particular financing functions.

### Political conditions shape financing for PHC

Politics and political conditions have crucial roles in explaining individuals' and institutions' behaviours. Three key political conditions influence the success of PHC financing initiatives in LMICs: the drivers of change, the mix of political actors, and the historical roots of the existing PHC financing system.

#### Political drivers of change

Change can be driven by different actors who represent various political powers, economic interests, or social movements. In some settings, such as Brazil and Costa Rica, strengthening PHC financing has been part of a consistent political drive to guarantee basic human rights and equity put forward by social movements that represent, or advocate for the interests of, grassroots populations, including poorer and marginalised groups.^231^ In both countries, providing essential public services, including investing in PHC, became part of the social contract between the state and its citizens and was supported by a diverse coalition of actors and enshrined in their constitutions.^107^, ^232^

In other cases, the change was driven by political leaders seeking to serve the interests of specific constituencies and expand their legitimacy and influence. For example, in China, political leaders initiated equity-oriented health financing reforms as a means to meet ambitious development goals. Similarly in Ethiopia, the federal government's development policies emphasised poverty reduction and support for community-based initiatives in the health, education, and agriculture sectors. This was enabled through a significant public investment in a comprehensive PHC platform organised around a new cadre of health extension workers^233^ and the Health Development Army of community volunteers. The health extension workers served as a focal point for expanded investments in health on the part of the government and its development partners, which are supported by the volunteers and local governance structures. The concept was rooted in the ruling party's political strategy of prioritising rural interests and seeking to unite ethnically diverse constituencies. This rural-focused strategy had previously enabled the party to govern territory successfully when operating as an insurgency,^108^ and was rooted in its Maoist and Marxist political ideologies.

Technocratic elites (actors who have both technical expertise and political leverage) and professional organisations, accompanied by effective bureaucracies with the ability and interest to operationalise reforms, can also initiate and drive change.

#### Aligning diverse political actors

Early involvement of actors from several sectors, including leaders, technical experts, and social activists from inside and outside the health system, has proven instrumental in transforming PHC financing in many countries. Enhancing health sector governance is an essential first step. Particularly critical is attracting a broad range of actors in support of PHC financing reforms to augment the pool of technical knowledge and skills that can support system functioning and transformation, and to foster unity among diverse interest groups. For example, as shown in the Ghana case study (presented in section 5), the introduction of capitation as the method of provider payment needed concurrence among the Ministry of Finance, medical professionals' associations, and patients.^189^ Getting their buy-in requires considering the economic interests of different actors. Therefore, managing up and building coalitions with the ministry of finance and other agencies as key partners, as well as addressing opposition, are essential when planning and implementing, for example, the introduction of capitation-based payment systems.

There are five key considerations related to bringing together the necessary mix of actors.

• Political will and legitimacy of the actors driving change are essential preconditions to generating a political process that enables collaborative policy making in support of effective policy. Ideally, this is combined with strong technical leadership at national and subnational levels of the health system. The leaders, benefiting from broad political support, and networks are key,^2^ and they do not need to be exclusively from the health sector. In Sierra Leone, for example, the removal of user fees in 2010 was actively led by the President Koroma, and supported by a wide range of actors, including the ministries of health and finance, donors, and civil society.^234^
• Policy development requires the engagement of an enthusiastic group of pioneers or policy entrepreneurs to provide both technical expertise and the vision for the reform. The central role of policy entrepreneurs or political champions in taking forward major policy initiatives such as UHC was also documented in Nigeria.^235^ Similarly, health financing reforms started in Estonia in the late 1980s when more opportunities for local decision-making started to arise within the Soviet Union.^182^ After the political transition, leaders from the University of Tartu, supported by external actors, drove the new vision of how PHC family practices should operate. The Ministry of Health funded early pilots before health financing transformations were initiated; the Ministry of Social Affairs and the Estonian Health Insurance Fund were also important supporters.^182^ Key individuals provided a strong vision and stewarded diverse reform processes, while enlisting a critical mass of professionals (namely, health-care providers and administrators interested in developing a sustainable financing system that would guarantee stable, earmarked funding for health) to provide support throughout the design and implementation of the reforms.^236^
• Political leaders and policy pioneers alike need to be supported by effective bureaucracies. Several countries that have achieved good health at low cost through investments in, and transformation of, PHC have bureaucracies characterised by strong managerial and implementation capacity, institutional memory, and openness to change. In both Thailand^237^ (Thailand's building of multi-actor coalition supporting PHC is outlined in the appendix [p 43]) and Estonia,^182^ bureaucratic elites and technocrats who could draw on their personal legitimacy provided both technical and political support, working with political actors and external experts to enable complex financing reforms. Conversely, in China, PHC financing reforms have been hindered by bureaucratic fragmentation, with local bureaucracies insufficiently incentivised and invested in developing solutions to the fundamental problems hampering PHC and with preference to invest in hospitals that attract political capital locally.^145^
• Strong civil society engagement is frequently catalytic in generating and sustaining broad-based political platforms that build support for and sustain momentum towards PHC financing reform. Civil society movements, including workers' unions and employers' associations, have led to the right to health (and in particular PHC) being enshrined in the constitutions of many Latin American countries (as previously highlighted in Brazil and Costa Rica). In Thailand, civil society advocacy was an important factor in creating structures at both national and community levels. In South Africa, civil society organisations such as the Treatment Action Campaign were crucial in bringing about the introduction of antiretroviral therapy.^238^
• Creating and sustaining structures and institutions that promote and support collaboration and dialogue was important in Thailand, Kyrgyzstan, Turkey, and other countries. Engaging key actors effectively in efforts to improve PHC financing functions entails aligning their interests and clearly defining their roles. In practice, the coordination function is often taken on by a dedicated transformation team. Although this team might have access to key political players, it also needs to be sufficiently independent to provide impartial advice and guidance and to weather shifts in power. For example, Thailand and Kyrgyzstan created research units within their Ministries of Health that were instrumental in guiding policy towards the achievement of UHC and sustaining an orientation towards equity.^2^ In Turkey, Minister of Health Akdag created a reform team that oversaw the implementation of the Health System Transformation Plan. Similarly, in Rwanda dedicated institutions directly supported decision making and coordinated national programme implementation related to PHC financing, including at the National University of Rwanda, School of Public Health and the Rwanda Biomedical Centre, Kigali, Rwanda.^239^ Such structures have been instrumental in generating evidence for financing reforms while maintaining independence from political processes.

Finally, collaboration is at the heart of the concept of a whole-of-government approach that transcends line ministries and other agencies' typical portfolio boundaries to achieve shared multisectoral goals.^240^ This is particularly crucial to PHC and its financing, in which engaging the whole government involves, in part, recognising the relative power of different ministries involved (particularly health, finance, and other social sectors such as education or water and sanitation), and ensuring that their interests align. In LMICs, the range of key stakeholders involved in designing and implementing PHC policies and programmes can be even broader, including key civil service agencies, donors, professionals, user associations, and civil society organisations—often disrupting the opportunities for joint action. This is particularly evident in areas such as the essential public health functions, a key component of PHC, under the remit of multiple sectors. Importantly, these intersectoral approaches and linkages need to underpin not only national strategic policies but be translated into structures and operational models at subnational levels such as district and local.

In Brazil, the municipalities are a key focal point in planning and implementing community-led multisectoral actions in response to local health needs and are governed by stakeholder committees.^241^ At the local level, the increasing importance of community health workers and multipurpose volunteers, whose role often incorporates tasks to promote social development and address social determinants of health which are core to PHC, and their linking in or integration within the health systems has meant that funding can be channelled more effectively to delivery of essential services (see sections 1 and 5). For example, in Ethiopia, this is seen in the collaborative community-based multisectoral models involving health extension workers, facility-based PHC providers, community governance structures, and volunteers working in close coordination to improve health and wellbeing of the population. This is underpinned by a national strategy for PHC and the Health Sector Development Program acting as a blueprint for how different actors should link to each other.

However, coordination among ministries alone is insufficient for effective PHC financing policy and strategic investment—it also requires coherence across policies. This can be achieved by having an overarching vision for PHC, including strategies for improving routine operation and enabling reform. Despite general recognition that comprehensive PHC requires multisectoral action, in practice coordinating financing across sectors and at different levels of government is relatively uncommon. One exception is China, where a cross-ministerial health system reform administrative mechanism has shown promise, with local actors (such as local vice governors and mayors responsible for PHC at the local level of government and the local health commissions) contributing to the ongoing development of integrated policies.^145^ In Brazil, participatory management councils at the municipal level were even enshrined in the country's constitution, enabling them to manage powerful local interests.^241^

#### Sustained vision, flexible strategies

PHC financing is subject to contestation that is dynamically changing over time. Policies promoted by one political coalition can be reversed when a new party comes to power. However, PHC financing is also path dependent, so initial decisions can determine the range of options that are available later. The importance of historical roots in fostering PHC that is people centred is seen in the UK's experience, where small general PHC practices funded by voluntary insurance were integrated into a national health system but preserved their autonomy.^242^ In many LMICs, colonial histories have had a major role in defining how health financing systems are organised. For example, Algeria and Morocco both have social insurance schemes that are similar to the Bismarck model, and the French system. Egypt, meanwhile, has a social assistance scheme inspired by the English Poor Law. Gaza uses an Egyptian insurance scheme, stemming from Egypt's occupation of Gaza through 1967.^243^ The structure of the health system and payment for this system remains based on the legacy of the colonisers.^243^

Although path dependency can mean that changes in PHC financing occur over a long period of time, with a series of reform steps building on each other, in some situations the direction of reform has changed at important junctures through radical socio-political or other crises, as in China (how path dependency has influenced China's PHC financing is outlined in the appendix [p 43]). Similarly, countries in Eastern Europe and the former Soviet Union had opportunities to rapidly overhaul resource mobilisation and purchasing arrangements for PHC after the political transition of 1989 and the collapse of the Union of Soviet Socialist Republics (political transition as an important juncture for PHC financing transformation in the Eastern European region is outlined in the appendix [p 44]). Although there is no doubt that compromise and aligning of actor interests and focusing on what is feasible are often required for changes to be politically viable, some of those compromises, although expedient, can greatly limit options later. Such limiting options for PHC financing include a focus on expanding population coverage by starting with formal sector employees, prioritising hospital services in the benefits package, and allowing for fee-for-service payments in the public sector.

Because of the dynamic nature of political processes, having a clear long-term vision, upheld and publicly stated over time, has been important in supporting the transformation of financing to enable PHC delivery models. Fostering effective financing functions and reform in line with this vision entails consistently engaging with politics and continually designing new technical solutions to emerging problems. In some cases, technical solutions can be developed while waiting for a window of opportunity to consider them to appear; in other cases such as Turkey's (Turkey's political strategy to strengthen PHC and its financing is outlined in the appendix [p 45]), rapidly changing political conditions create demand for novel technical solutions.

Once a clear vision has been agreed, moving towards PHC financing requires strategies for staying on course while retaining flexibility. Therefore, it involves maintaining direction of travel through periods of political stability and economic growth, taking advantage of windows of opportunity created by adverse political events and crises (such as COVID-19), and persisting through periods of stagnation. The balance of power among different groups also evolves over time, leading to the emergence of new agendas, new actors, and new coalitions. Reformers who have candidate technical solutions can slowly gather support for reforms that are initially unpopular or require strategic compromise. The case of the Seguro Popular in Mexico, for example, highlights the importance of strategic compromise and targeted negotiation to move reform processes forward, from abandoning the idea of merging the Mexican Social Security Institute and other social security programmes into a single organisation, to allowing enrolment without premium payment for almost the whole population.^225^ Brazil, Ethiopia, and China offer examples of countries where comprehensive reforms have been implemented and refined over decades, maintaining a consistent direction while adapting to political and socioeconomic transitions and considerable uncertainty.^107^, ^118^, ^145^

Examining the early efforts of countries that have successfully implemented long-term PHC financing and delivery transformation reveals the importance of building strong foundations to support ongoing changes. These foundations can be technical (ie, ensuring that the technical features of the reform were ready to be used at the earliest opportunity, as in Turkey), or can involve investing in PHC delivery capacity ahead of the reform, which helped Thailand, Ethiopia, and Estonia to absorb additional financial flows and enable large-scale shifts to PHC-focused health systems. In both cases, this involved creating new PHC cadres (eg, health extension workers in Ethiopia and family practitioners in Estonia) that were deployed nationally, instituting training and supervision structures, and embedding best practice norms and clinical standards. In addition to training human resources and extending PHC infrastructure, these activities generated visibility for the early reforms that improved buy-in among different constituencies. Thailand developed standard designs for PHC infrastructure and built many facilities, making services widely accessible with over 10 000 PHC facilities—one per 6000 population—linked to strong community-based volunteer programmes and widespread public health interventions such as dengue control.^244^ These reforms created public support for PHC and willingness to allocate new resources.

Finally, identifying strategic compromises has also characterised many countries' efforts in financing PHC. For example, in Estonia, policy makers passed iterative step-wise health-care reforms from the early 1990s to the end of the 2000s, strengthening the PHC system. In the early stages they adopted an explicit tactic of lying low and not inviting publicity and identifying paths of least resistance for implementation until a critical mass of supportive PHC providers (general practitioners) emerged.^245^ Compromise can be achieved through iterative processes of piloting, evaluating, and adapting PHC financing innovations before their scaling up—Rwanda, Burundi, and Burkina Faso all did this when introducing results-based financing.^246^ In China, provincial-level pilot programmes were evaluated, results disseminated, and experiences shared.^145^ In Thailand, high-level bureaucrats started implementing reforms (including payment reform) as part of a national pilot, and were able to refine the policies before they had to be approved by Parliament. In Brazil, reformers started implementing pilots of the proposed reforms in selected municipalities. These pilots were then enabled by the 1988 Constitution, which established a national health system for the entire country.^245^

### Social and economic conditions influence PHC financing

In addition to political conditions, inter-related social and economic factors can also support or hamper routine operation and reform of PHC financing (figure 13).

#### Social conditions

A range of social conditions influence financing arrangements. These include: the degree of inequality in a society, the availability of health workers with the capacity to implement reforms, prominent social grievances that propel certain issues to centre stage, and the strength of the social contract between the state and the population, among others.

Inequalities within a nation or society can provoke dissent against the status quo and foster support for reforms aimed at redressing the problems. China's 2009 health reforms stemmed from widespread complaints from the population about severe inequality in access to health care, as out-of-pocket payments continued to cripple households financially, accounting for about 60% of total health expenditure.^247^

Similarly, in Brazil, the thirst for greater equity among segments of the population and better representation after decades of dictatorship propelled successful adoption of broad health system reforms based on principles of health care as a citizen's right and a government responsibility. This formed the basis for a universal, comprehensive, and decentralised health system open to both community participation and, to some extent, private sector initiatives.^107^

Social grievances against the state or government bodies could also influence the success of a financing reform. Turkey tried to roll out a Family Doctors Programme, which included a swathe of financing and other health reforms, after unpopular decentralisation and privatisation policies had been implemented in the health sector. Those processes had created significant dissatisfaction amongst the population, as well as medical associations and physicians. The Family Doctors Programme was perceived as a similar strategy and met resistance.^248^ In Nigeria, a distrust of the national government led to the resistance of civil servants at the subnational level towards making contributions to the national health insurance scheme that would guarantee access to PHC, prevented the adoption of the scheme by subnational governments, delayed coverage expansion, and left the scheme as a voluntary programme.^249^

The strength of the social contract between the population and the state can affect how reforms are received. In the state of Kerala, India, for example, the strong social contract between the state leadership and the population has been partly credited for its early successes in tackling the COVID-19 pandemic.^250^ In the Middle East and North Africa, political regimes that came to power after independence also established social contracts that included providing material benefits, such as expanded access to primary and secondary health care.^243^ The weakening of these social contracts over time has driven widespread resistance to more recent reforms, and contributed to the 2011 uprisings across the region.

The existence of certain capacities (in the society at large and the health sector in particular) is also important when promoting change. Any health financing reform, such as the introduction of a new budgeting approach or a new provider payment structure, requires skilled staff to communicate and manage the transition and to implement the new approaches. As mentioned in section 3, having insufficient technical budgeting capacity at the Ministry of Health, for example, weakens its bargaining position during national budgeting cycles. This was the case in India, where the scarcity of capacity of the states to prepare health budgets and plans aligned with the central government's expectations has weakened their ability to obtain resources for PHC.^126^

Importantly, crises of any type can be transformed into opportunities for PHC reform if reformers are poised to act. In the UK for example, the National Health Service was created following the hardship of World War 2.^251^ In Costa Rica, a 1991 measles outbreak led to employers across the country being forced to pay for private care for their workers due to weak public PHC.^252^ Employers then threatened to stop making their social security contributions, contributing to government investment in a comprehensive PHC system. The Chinese health reform of the early 2000s, which involved substantial investments in PHC, was triggered in part by the 2003 SARS epidemic.^253^

The COVID-19 pandemic has been a particularly severe global shock that has affected societies and economic outlook, as discussed throughout the report. The political economy of the pandemic itself, and the response to its devastating consequences, has also started to be scrutinised, and will require further exploration.^254^ For now, it has triggered initiatives and debates on how PHC needs to be transformed to cater for changing needs.^255^

#### Economic conditions

National and global economic conditions have significant influence on health financing. These conditions include the structure of the economy, economic cycles of stagnation, recession, or growth, the structure of the health care provider market, the size and dynamics of the private sector, and the importance of aid as a source of financing for health.

As discussed in section 3, the structure of a country's economy has a substantial impact on financing for PHC. In low-income countries, only a small portion of the population and private sector organisations are subject to taxes. In Tanzania, for example, just 286 organisations contribute 70% of domestic tax revenue.^98^ Those who do pay taxes in countries with small tax bases have substantial power in driving what reforms can be implemented. The level of informality in the labour market dictates whether any type of labour employment tax or health insurance contributions will generate sufficient revenue to support social health insurance. In Ethiopia, for example, attempts to implement social health insurance have stalled for many years in part due to the high level of labour informality.

In addition to the structure of a country's economy, where the country is in the economic cycles of growth and contraction can influence the success of a reform aimed at increasing financing for PHC. In Brazil, the fiscal space generated from sustained economic growth during the 2000s enabled the country to increase its public health expenditures.^107^ In Chile, similarly, important PHC reforms were made possible by high economic growth during the first decades of the country's return to democracy.^55^ However, in Finland, the collapse of trade with the former Union of Soviet Socialist Republics in 1992 led to a steep economic decline and, as a result, national expenditure on health care was slashed by 12% in 1991–94.^256^

The structure of the health-care provider market also influences reforms, particularly those to do with purchasing. On the one hand, in Estonia, the Association of Family Doctors had a major role as a partner to the national insurance agency in designing and implementing laws to support PHC financing.^182^ In Ghana, on the other hand, the design and implementation of the national health insurance scheme was influenced by the strong bargaining power of providers, who preferred being paid on a fee-for-service basis and resisted a system change that would affect payment mechanisms for PHC.^257^

Finally, the size and dynamic nature of the private sector is another critical economic factor that can influence health financing reform efforts. In Thailand, for example, the capitation system was designed to improve quality of care through fostering competition among PHC providers. This was possible in Bangkok, where a large number of private providers were willing to accept patients with insurance (as the capitation system was part of the national health insurance scheme).^184^ In Ghana, on the other hand, the shortage of public and private providers in rural areas constrained competition during the capitation pilot in Ashanti region.^189^

### Applying political economy analysis to advance financing for PHC

As noted, political economy analysis seeks not only to explain but also to derive practical implications for strategic policy making. It explains how the political and socioeconomic contexts shape what is possible or not in developing and implementing key policies. Although some cross-country lessons can be drawn (with caution) from the country examples discussed throughout this report, the critical influence on PHC financing functions of the political, social and economic conditions that make up the political economy are best understood within each national, and often subnational, context. Analysis of these factors should be an integral ongoing part of implementing feasible interventions to improve efficiency and equity of PHC financing. A common thread is that there is no blueprint approach to changing PHC financing, and even within a country, the political economy context also changes over time, often rapidly.

Political economy analysis can be used to inform proactive or responsive strategies for adaptive management of the interests of different actors and formulating strategies that fit the social and economic conditions in support of health financing reforms. A political economy lens focuses on understanding the structural and socioeconomic conditions underpinning decision making and conflicting interests. This understanding might lead to, for example, the use of strategies to strengthen actors with limited power but who would benefit most from more effective PHC financing. In other cases, political economy analysis may indicate the need to anticipate and manage resistance from those who benefit from the status quo, and to identify opportunities to form coalitions. Practical approaches to using political economy analysis to underpin routine operation or transformation of PHC financing are outlined in the appendix (p 45).

Designing politically informed technical strategies starts by asking the right questions to navigate the complex political economy context (Sparkes S, WHO, personal communication).^258^ In this Commission, we have formulated a series of key questions that should be asked throughout the policy cycle, and can be used for setting the agenda as well as designing and implementing people centred PHC financing policies:

• What is the problem to be addressed? What ideas exist for improving PHC financing? What technical strategies would achieve this over time?
• Who are the stakeholders with an influence over the problem? What are their positions on the topic, and what is their relative power?
• What are the political dynamics at play?
• What could help to shift incentives to promote the changes pursued?
• What social and economic conditions that underpin the political process could present opportunities or constraints for the proposed change?
• What are the most likely pathways for change? What are possible entry points to move the reform forwards? If there is a potential window of opportunity, how can it be used to generate and sustain political momentum?
• How do the proposed strategies take into account path dependency?
• How should the strategies be sequenced?

Although awareness of and the need to conduct political economy analysis is a part of developing policies for sustainable and equitable PHC financing, country-level capacity to do so can be scarce. Investing in basic supportive functions, which include technical capacity to do political economy analysis, ability to engage with actors and policy processes, and translate knowledge to policy is key. Importantly, there is often knowledge of political economy analysis developed in other sectors that can be used in policy development in the health sector. Support for, and retention of professionals who can develop and integrate specific aspects of political economy analysis in their work should be ongoing. Subnational entities such as districts can be incentivised to co-fund training and recruitment of managerial or research cadre able to use particular political economy analysis skills, relevant to context, as a part of leadership programmes. We argue that sustaining political economy analysis is key as a part of investment in both system transformation and strengthening routine financing systems; it is particularly important in effectively responding to large-scale shocks such as pandemics or political transitions.

### Conclusion

Technical strategies for efficient and equitable financing for PHC are neither designed nor implemented in a vacuum. They are critically shaped by political, economic, and social conditions—and the dynamic nature of these forces can create opportunities to maximise impact, or impose barriers that constrain success. Applying a political economy lens to technical solutions that explicitly recognises the evolving roles of actors beyond the health system, their relative resources and power, as well as the economic constraints and social relations, is therefore necessary to strengthen the PHC financing architecture.

## Section 7: Recommendations

The Commission's deliberations have analysed how health financing arrangements can be used to drive national health systems to provide equitable, comprehensive, integrated, and high-quality PHC, delivered through platforms that are responsive to the needs of the populations they serve, and fully aligned with the objectives of UHC.

**Figure 14:**
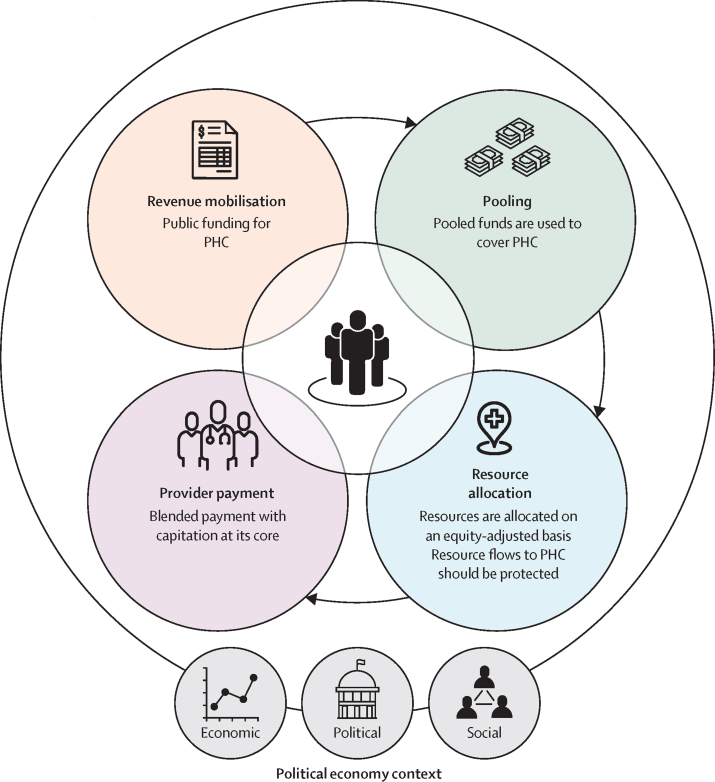
Framework for people-centred financing of PHC

We argue that countries should invest more and invest better in PHC, and that the financing arrangements that support PHC—from mobilisation and pooling of resources, to budgeting, allocation, and purchasing—must place people at the centre. They must also be driven by a focus on equity and social justice, in line with the original Alma Ata vision. We articulate the features of people-centred financing for PHC below. We recognise that the opportunities to reorient health financing policies towards PHC depend on the economic, social and political features of a particular regional, national, or sub-national context, and that there is no single pathway to achieving optimal PHC financing (figure 14).

An overarching position of the Commission has been the principle of progressive universalism: This means that governments should prioritise equity by providing universal access to affordable, quality PHC services, and particularly on ensuring that disadvantaged groups are reached first. Guaranteed entitlements can expand beyond PHC as fiscal capacity increases.

Beyond this overarching principle, the Commission has identified four key attributes of people centred financing for PHC.

First, public resources should provide the core of PHC funding with minimal reliance on direct payments when services are accessed. In most LMICs, this level of public funding can only be generated through increasing allocations to PHC from general tax revenue. Revenue raising mechanisms should be defined based on ability to pay and be progressive. While each country is at a different starting point for a shift to predominantly public funding, strategic and purposeful change to national health financing systems over time can enable gradual progress. In the meantime, low-income countries will require continued development assistance to secure a sufficient resource envelope to enable population coverage of essential PHC services.

Second, pooling arrangements should cover PHC. By supporting PHC with pooled public funds, out-of-pocket payments can be reduced to levels at which they do not pose financial barriers to accessing needed care, impoverish households, or push households deeper into poverty. Pooling enables cross-subsidy among those who are well and those who are ill, and among the poor and the wealthy.

Third, resources should be allocated equitably across levels of service delivery and geographic areas, and be protected to reach frontline PHC service providers and patients. Resource requirements for PHC should be estimated on the basis of accurate assessments of population health needs. Countries should deploy strategic resource allocation tools (including needs-based per-capita resource allocation formulae) in budget formulation, budget execution, public financial management policies, and service delivery arrangements to channel and protect the flow of resources for PHC.

Fourth, provider payment mechanisms should: assign resources based on people's health needs; create the right incentive environment to promote PHC that is people-centred along the spectrum of prevention, health promotion, and treatment; foster continuity and quality of care; and be flexible enough to respond to changing needs of patients, families and communities. This is best achieved through a blended provider payment system with capitation at its core.

Table 2 presents a matrix which maps how financing arrangements can be deployed to achieve the dual goals of people-centredness and equity.

**Table 2:** The Commission's vision of financing functions and arrangements for PHC that is people centred and equity driven

	**Mobilisation**	**Pooling**	**Allocation**	**Purchasing**
People-centred characteristics	Resource requirements for PHC should be estimated based on what is needed for each person to access; PHC that is people centred as defined in the country's context	Everyone is included in the pool	Resources are allocated based on population needs for PHC that is people centred (rather than on facilities, inputs, or vertical programmes)	Purchasing arrangements and provider payment mechanisms are linked to making PHC that is people centred available to people and flexible enough to accommodate different modes of delivery; funds flow to and are managed by frontline providers as defined in the country context
Equity and progressive universalism characteristics	Revenue-raising mechanism is defined based on ability to pay and is progressive	Cross-subsidisation occurs between poor and wealthy populations and healthy and sick populations; use of pooled funds prioritises making PHC accessible, with financial protection and subsidies directed to the poor	The mechanism used to allocate public funds prioritises the needs of the poorest segments of population, and areas (geographical or health) of greatest need	Per capita payment (capitation) is the starting point, which makes the same amount of funds available to providers to deliver the PHC package for each person (adjusted upward or downward according to health needs)
Practical implications and anticipated outcomes	Reduces out-of-pocket expenditure; progressive taxation policies	Merge or consolidate existing pools into larger pools (including formal and informal sectors, poor and rich; coverage dominated by public financing); who and what are covered by the pool expands in the most equitable and PHC-centric way; progressively move to universal health coverage according to the macro-fiscal capacity of the country, starting with access to PHC for all and financial subsidies directed to the poorest and most vulnerable; access to more services beyond PHC and subsidies for more population groups can expand as macro-fiscal capacity expands	Budgeting is based on needs-based per capita allocations to enable access to PHC that is people centred (rather than to facilities, inputs, or vertical programmes); protect resources going to PHC through existing policy tools, such as programme budgets, resource allocation formulae, conditional grants or statutory rules; define a benefit package that prioritises coverage of the needs of poorest segments of population; ensure resources reach frontline providers (through direct facility financing, for example) and improve public finance management systems more broadly; organise service delivery to pull resources to PHC, for example by creating new cadres of frontline PHC providers, defining explicit service standards, or instituting effective referral systems	Establish a blended payment model with capitation at its core: start with a baseline capitation payment . The payment amount should be determined using a formula that links the payment parameters (base per capita rate, number of enrolees linked to the provider, and any individual or provider-level adjustments) to a defined package of PHC services; define a PHC package; adjust the risk level to prioritise those in greatest need

PHC=primary health care.

Together, these attributes form the foundations of a resilient and responsive health financing system. As has become evident during the COVID-19 pandemic, effectively financing PHC during a crisis period relies on the existence of a health system that is capable of surging to tackle new priorities while also continuing to deliver existing services. Resource mobilisation, pooling, allocation, and purchasing systems must be able to respond quickly to deliver additional resources while protecting allocations to PHC.

We acknowledge that there is no single pathway to achieve optimal PHC financing, and that every country is at a different point in orienting its PHC policies and financing. Moreover, it will take time to adapt financing arrangements, and these will need to continue to adapt to changing conditions and needs over time, and to respond to shocks. However, by moving deliberately towards publicly-financed, progressively universal, and population-based health financing, countries can support the expansion and improvement of PHC.

In support of this vision, the Commission makes five recommendations to local, national and global policy makers and other relevant stakeholders (panel 20). We recognise that many of the recommendations could potentially be applicable to health system reform in general. However, we continue to focus specifically on PHC, while endeavouring to avoid separating it out from the rest of the health system.

Panel 20 **Our vision for people-centred financing of primary health care (PHC)**

PHC needs both more and better resources. The Commission's vision is of a people-centred system for financing PHC. This system should be capable of collecting, pooling, and allocating resources to purchase services that ensure that all people (community members, patients, and providers) are able to benefit. Progressive universalism—ensuring that, at every step, people who are poor or vulnerable gain at least as much as those who are wealthy or privileged—is at the core of this vision. Achieving this vision requires:

• An adequately-financed health sector, funded by expanded public and pooled sources, that protects everyone from financial hardship when seeking care. The Commission argues for an explicit focus on addressing inequities first. This entails that revenue will be raised based on ability to pay and through progressive means.
• Pooled funding will cover PHC, to enable everyone to receive PHC that is free at the point of use. Pooling of resources will support cross-subsidisation among those are well and those who are ill, and among the poor and the wealthy.
• A strategic use of all available policy tools to direct sufficient resources to PHC to enable a universally-accessible system that provides high-quality services according to a defined benefit package appropriate to the level of care and aligned with macro-fiscal capacity. In line with our core focus on people-centeredness and equity, the Commission proposes that resources are allocated based on population needs, prioritising the needs of the poorest segments of the population. To do so will require mechanisms for funding, budgeting, and financial management that ensure that resources reach frontline providers and platforms.
• A context-specific blended payment model built on capitation. Payment systems should allow adequate resources to flow to the PHC level in ways that: are equitable; match resources to population health needs; create the right incentive environment to promote the full PHC spectrum of prevention, health promotion, and management and treatment; foster people-centeredness, continuity, and quality of PHC; and, are flexible enough to support changes in service delivery models and approaches.
• A nuanced understanding of the political economy of each country throughout the development and implementation of all policy to accompany the technical approaches to ensuring people-centred financing for PHC.

### (1) Establish people-centred financing arrangements for PHC that have four key attributes

Establish financing arrangements for PHC following a principle of progressive universalism and incorporating the people-centred attributes outlined above. These are: public resources provide the core of PHC funding; pooled funds should cover PHC; resources should be allocated equitably and protected so they reach front-line providers; and provider payment is through a blended mechanism with capitation at its core.

### (2) Take a whole-of-government approach to spending more and spending better on PHC

Key actors and stakeholders should be involved in designing and implementing people-centred PHC financing reforms. Although the specifics will vary depending on the national context, some general roles and responsibilities can be identified.

The ministry of health should lead efforts to prioritise PHC. Leadership involves promoting technical strategies embodying the above principles, ensuring that sufficient resources are made available, and elaborating and pursuing political strategies in support of expanding and improving PHC financing. The ministry of health should ensure it has the technical expertise to make the case for more funding for PHC. To ensure accountability, which sections within the ministry of health are responsible for the financing and delivery of PHC should be clarified. The ministry should take responsibility for engaging the commitment of the other sectors (such as education and water and sanitation) whose activities relate to PHC.

The ministry of finance should enable the mobilisation of sufficient revenue to adequately finance people-centred PHC, as defined nationally. The ministry of finance should work with the ministry of health and other agencies to develop flexible and responsive public finance management systems that make allocations to PHC visible, protect resource flows to reach the frontlines, and allow strategic provider payment systems that evolve as capacity grows and service delivery models mature.

Local government agencies should serve as bridges between local populations and central government ministries. Local authorities are well-positioned to identify and communicate populations' needs so they can be accurately captured in allocation formulas. Local agencies are also responsible for integrating multiple funding flows and ensuring they are applied to addressing local priorities.

Communities and civil society groups should demand changes. Communities should engage in efforts to hold PHC providers accountable, and be included as partners in monitoring progress. To that end, a key priority for civil society groups should be building the capacity of communities to undertake these functions. Governments should facilitate this accountability by producing data to enable monitoring.

Health-care providers and their representative organisations should actively participate in efforts to reform PHC financing arrangements. Providers must engage in the design of payment reforms, understand the implications of proposed changes to provider payment systems, and take any and all opportunities to provide people-centred health care fostered by reformed financing arrangements.

Donor and technical agencies should provide financial resources and expert technical support to countries that need assistance to jumpstart changes in PHC financing. At country level, agencies should ensure that, at the very least, their actions do no harm. Agencies should immediately change their approaches to providing financial and technical assistance to reduce fragmentation. At best, donor and technical support agencies can be strategic partners to the national governments working to improve their financing systems to support PHC that is people centred.

### (3) Strategically plot out a pathway towards people-centred financing for PHC, including supporting basic health system functions

Each country should articulate a vision for financing PHC. Having a clear vision allows decision makers to plot a strategic technical path and identify what political engagement is needed from stakeholders throughout the system to support progress.

National funding champions of PHC should proactively explore and recognise the political, economic, and social conditions at subnational, national, and global levels to effectively navigate towards the vision through the evolving political economy context. This political economy analysis should begin at the outset of any reform process.

Implementing politically-informed technical strategies (ie, strategies rooted in a thorough understanding of the political economy context within which the technical approach sits) should involve regular mapping of the political landscape, assessing opportunities to align interests and build coalitions among actors from different sectors and administrative domains, and communicating and working towards collaboration and strategic compromises in support of key technical policies. This might require strengthening the skills of people working in government, and in academic and donor partners, to undertake political economy analysis.

The country's vision should be operationalised by mapping out a clear set of steps to pursue its chosen course, while also preparing to capitalise on unexpected opportunities and creating room to manoeuvre as needed to adapt to political and socioeconomic changes, crises, and other shocks.

All stakeholders should undertake ongoing efforts to strengthen the health system's basic functions, including data collection and analysis of resource flows and health impacts; monitoring, evaluation, and learning systems; public finance management systems that enable resources to reach frontline providers; and capacity of providers to manage funds effectively. Investment in such basic functions is needed in conjunction with investment in PHC.

### (4) Global agencies should reform the way PHC expenditure data are collected, classified, and reported

A new reporting item (ie, memorandum item) on PHC should be defined and included in countries' annual reporting of health expenditures to WHO for the Global Health Expenditure Database.

In the meantime, current reporting should be adapted to be based on a cross-classification of functions and providers (such as the one used by the OECD). This will provide more specific and useful data by, for example, allowing for differentiation between hospital and ambulatory providers, and enable an operational definition of PHC based on service delivery platforms.

The categories currently included in the calculation of PHC expenditure should be revised. In particular, how administrative costs are included and how outpatient services in hospitals are classified should be reconsidered, as current practices skew estimates of PHC spending upwards.

Most importantly, each country should establish a clear definition of PHC expenditure that is compatible with how its health system organises services; it can then use this definition to track spending over time to monitor progress.

### (5) Conclusion

The Commission recognises that its work represents the beginning, not the end, of a research agenda on financing people-centred PHC. The Commission's explorations raise many additional questions, starting with those presented in panel 21. The Commission proposes the following next steps and starting points for additional exploration by key stakeholders, including academic researchers, technical experts, policy makers, donors, and others:

• Creating a tool for national mapping of PHC financing ecosystems to create a firm data foundation for developing appropriate technical and political strategies to advance people-centred financing in support of PHC. Collaboration among researchers, technical experts, and policy makers is needed to develop a robust tool and method.
• Exploring and devising innovations to support better PHC financing, including adopting implementation science and other operational research methods.
• Securing funding from governments and other donors for rigorous research on the research questions suggested in panel 21, as well as others that will arise.

Panel 21 **Research questions on financing for primary health care (PHC) that is people centred**

The Commission's work has answered some questions, particularly on technical aspects of financing arrangements. But it has raised many others, most especially on how to operationalise our recommendations. These are the ‘how do we do this?’ questions. Going forward, the research agenda on financing PHC should study the outcomes of proposed reforms and examine implementation at country and local level. Key topics and questions include:Innovative approaches to support the delivery of PHC:

• What are the best ways to channel funds directly to facilities?
• What are the effects on health outcomes of getting money to frontline providers?
• How can digital innovations be used in health financing systems, while promoting universal health coverage and minimising fragmentation?Spending more and spending better on health in general and PHC in particular:
• What bottlenecks in health financing reforms arise in particular settings? What are the best approaches to counteracting them?
• What strategies that have been effective in ensuring financing for essential public health functions and linking them to PHC can be replicated, and how?
• What methods and data work well in measuring implementation of health financing reforms and tracking how funding flows change (including volume, recipients, timeliness, and equity)? How can these methods be used at the local, national, and global levels?
• Why are reforms addressing even well-known financing inefficiencies difficult to implement, and how can the reforms addressing these inefficiencies translate into additional financial resources for PHC?
• How can researchers collaborate effectively with policymakers to evaluate potential solutions and respond to their priorities and concerns?The political economy factors of financing PHC:
• What strategies have been used to effectively manage political economy considerations of providers and patients in health reforms? What are patients' and providers' understandings and attitudes towards proposed financing reforms?
• How can local actors be supported to foster investment and allocation in PHC? What power shifts are needed and which technical capabilities are most relevant?
• How do local and central government bodies interact in designing and implementing health financing reforms? What political economy considerations are important when seeking to influence resource allocation in decentralised settings?
• What are the political economy considerations for efforts in LMICs that seek to address fragmentation in pooling and shifting provider payment mechanisms towards capitation? What approaches can be effectively used to manage these factors?

In this report, we have set out a vision for placing people at the centre of the arrangements for financing PHC. This financing vision serves a greater ambition: health systems that provide equitable, comprehensive, integrated, and high-quality PHC delivered through platforms that are responsive to the needs of the populations they serve and fully aligned with the objectives of UHC.

## Declaration of interests

KH, DB, NB, DE and TP-J were funded by a grant from the Bill & Melinda Gates Foundation. DH has received funding from the Bill & Melinda Gates Foundation for various activities at UNICEF, including on health system strengthening and community health, both of which are mentioned in this report. In the period during which the report was developed, AE and HWa were employed by the Bill & Melinda Gates Foundation and were involved in data analysis, interpretation and writing of the report. All other authors declared no conflict of interest.

## Footnotes

* The Thailand case study was provided by S Viriyathorn, A Kulthanmanusorn, W Patcharanarumol, and V Tangcharoensathien, International Health Policy Programme, Ministry of Public Health, Thailand.
