# Correction to *Lancet Glob Health* 2022; 10: e715–72

**DOI:** 10.1016/S2214-109X(23)00035-9

**Published:** 2023-01-13

*Hanson K, Brikci N, Erlangga D, et al. The* Lancet Global Health *Commission on financing primary health care: putting people at the centre.* Lancet Glob Health *2022; **10:** e715–72*—In this Commission, Lauren Hashiguchi's name had been misspelt in the Acknowledgments section. This correction has been made as of Jan 13, 2023.

