# Supplementary material for: The Lancet Global Health Commission on financing primary health care: putting people at the centre
Source: Lancet Glob Health. 2022 Apr 4;10(5):e715–72. doi: 10.1016/S2214-109X(22)00005-5 (PMC9005653; doi:10.1016/S2214-109X(22)00005-5)
Supplement: Supplementary appendix [file mmc1.pdf]

THE LANCET
Global Health

Supplementary appendix

This appendix formed part of the original submission and has been peer reviewed. We post it as supplied by the authors.

Supplement to: Hanson K, Brikci N, Erlangga D, et al. The *Lancet Global Health* Commission on financing primary health care: putting people at the centre. *Lancet Glob Health* 2022; published online April 4. https://doi.org/10.1016/S2214-109X(22)00005-5.

The *Lancet* Global Health Commission on Financing Primary Health Care: Putting people at the centre

Appendix

Contents

Appendix 1. Financing PHC in times of COVID-19 2

Financing PHC in Sierra Leone to address entrenched and emerging challenges 2

COVID-19 and health funding in South Africa 3

Resource mobilization in times of crisis requires flexibility in budget systems and donor support 3

Resource allocation in times of crisis—getting resources to frontline providers 4

Appendix 2. Estimating PHC expenditure 5

Descriptive analysis of PHC expenditure 9

Appendix 3. Survey of PHC Organisation and Provider Payment 13

a. Study design and participants 13

b. Questionnaire development 13

c. Definitions: providers and payers 13

d. Data collection 14

Appendix 4. Digital technology and health financing 16

Virtual pooling with digital technologies 16

Digital technologies and revenue mobilization 16

Digital technologies to support provider payment and strategic purchasing 16

Methodology: Expert roundtable discussion on digital technologies for financing PHC 17

Appendix 5. Review of inefficiencies in health 18

Appendix 6. Political Economy Analysis on Financing PHC 25

Thailand's multi-actor coalition supports stronger PHC financing 25

Path dependency influences China's PHC financing[24-26] 25

Political transition as a critical juncture for PHC financing transformation in Eastern Europe 26

Turkey's efforts to establish PHC: vision and strategic compromise 27

Using Political Economy Analysis (PEA) 27

References 30

Appendix 1. Financing PHC in times of COVID-19

Financing PHC in Sierra Leone to address entrenched and emerging challenges

Sustainable financing for PHC in Sierra Leone is critical to enable the country's health system to meet its responsibilities to provide ongoing services and to mount responses to emerging threats. Effective PHC services often help bridge the gap of mistrust (a characteristic of settings described as conflict-affected, or which have experienced repeated shocks) between service users and the health system. The national response to COVID-19 in Sierra Leone, for example, was specifically designed to ensure continuity of care at all levels of the health system. This approach was informed by lessons learnt during the 2014 Ebola Virus Disease outbreak. PHC played a pivotal role in the Ebola response, including deployment of community health workers (CHWs) as contact tracers and social mobilisers, and the use of Community Care Centres for triage and isolation. The myriad roles and responsibilities of PHC level in Sierra Leone underline the importance of ensuring sustainable, people-centred financing for community health.

In recent decades, Sierra Leone has made significant commitments to health—and has backed up these commitments with investments. The country sustains a high level (10.8%) of total government expenditure on health. With the government committed to the spending goals of the Abuja Declaration, it is anticipated that the fiscal space for health in the future will not be negatively affected by COVID-19. However, only 42% of the total budget allocated for health in 2019 was actually spent—and this increased only slightly to 45% in 2020. A significant proportion of government funding for health in Sierra Leone is allocated to local authorities. In 2019, 74% of total government expenditure on health was transferred to local councils for services, including PHC services; this figure increased to 84% in 2020.

In general, resource allocation to PHC in Sierra Leone is informed by the number of health facilities, the services provided, and the populations served. Consistent and sustainable financial support for the CHW programme is particularly essential to the delivery of PHC, as CHWs support all PHC services. Because the CHW programme is heavily supported by donors, however, it can experience fragmentation in implementation.[1] Training, incentives, supervision, and supplies for the country's CHW programme are supported by various donors, including the World Bank, the Global Fund, the UK Foreign Commonwealth and Development Office, UNICEF, and USAID. Many challenges have been encountered by CHWs in the programme, such as not receiving their financial incentives after the end of the EVD response[2]; a similar situation has also been observed during the COVID-19 response.[3] Accordingly, more national investment is needed to support ownership and leadership of the CHW programme, especially in light of a new CHW policy currently being developed. Securing financial resources—enabled by annual cost modelling, setting annual financing targets by funding source, and identifying specific financing mechanisms to fulfil these targets—is necessary to ensure that funding for PHC is sufficient, comprehensive, and fully recognizes the valuable role that CHWs play in fighting disease outbreaks and promoting health in communities.

COVID-19 and health funding in South Africa

In the Republic of South Africa, the health security crisis generated by COVID-19 had a paradoxical effect on PHC funding. In 2020 an additional R21.5 billion (US$1.5 billion) was allocated to health for the national COVID response; substantial portions of this covered diagnostic testing, oxygen, personal protective equipment, community screening, and related programmes. However, the economic contraction caused by almost a year of shutdowns saw national revenue fall, and the economy is not projected to return to baseline until 2024. Approximately R76 billion (US$5.3 billion) was reduced from government health budgets in the 2021 medium-term budget.[4] These cuts have complicated the national response to the third wave of COVID-19 and potentially inadvertently weakened the national vaccination programme in 2021. Further, the cuts are likely to prejudicially affect PHC funding and to inhibit the National Health Insurance strategy for at least the next three years. Similar scenarios are playing out in many countries.

Several multinational agencies have called on governments not to pull back additional COVID-19 spending too quickly, noting the critical role played by the health sector in the economic recovery.[5] WHO has cautioned against the use of austerity budgets following the COVID-19 crisis, highlighting the known harmful effects of austerity on health budgets—and health outcomes.[6] In recent months the IMF, the World Bank and the OECD have all issued similar calls, emphasising the importance of the health sector in turning around the COVID-19-induced economic crisis and the need to avoid withdrawing social support, health, and fiscal stimulus spending too early.[7]

Resource mobilization in times of crisis requires flexibility in budget systems and donor support

The resources needed to respond to a health shock as well as maintain existing health services can be substantial. A recent World Bank estimate shows that sub-Saharan African countries may need about 3% of GDP, or US$53 billion, of additional funding for prevention, treatment and surveillance of the COVID-19 pandemic.[8]
Systems that are adequately funded are better able to withstand shocks.[9] Yet in many LMICs, OOPs spending represent a substantial share of total health expenditures (THE) and in times of crisis, private expenditure tends to increase.[9] The literature also shows that unfortunately, communities provide a key resource for coping financially with the cost of care during crises.[10] However, shocks are also often accompanied by falls in employment, and the reduced ability of people to pay for health care.[11]

Public funding to cope with the crisis is therefore of even greater importance at times of crisis. In response to COVID-19, the most common early approaches to cope with the increased need of funding from government budgets has been the reprioritisation of existing funds and *virements* between line items or within budgetary program envelopes, deficit financing, and increased flexibility in the use of funds. [12,13] Some countries opted for the use of Extra Budgetary Funds (EBFs) (set of accounts or a government entity engaged in financial transactions that are not included in the annual budget law)[14] or the reductions of 15%-30% of their operating budgets for all ministries not related to the COVID- relief

effort.[13] In Indonesia for example, the government halted non urgent spending and reallocated up to US$650 million to COVID-19 relief.[13] While substantially increased health and social spending to protect livelihoods was a feature of the response to early waves of infection in many countries in 2020, health budgets in 2021 or 2022 have in several cases become constrained due to deteriorating economic growth and fiscal and debt position.

The COVID-19 pandemic saw an unprecedented response from the donor community.[13] Donor aid in times of crisis is indeed essential and can be accessed through Disaster Risk Financing mechanisms (such as the Pandemics Emergency Financing Facility , the Catastrophe Deferred Drawdown Options or the Contingency Emergency Response Components), although there is growing evidence on their limitations which include their inadequate scale and tendency to support short-term, piecemeal interventions, and their lack of flexibility.[15]

Resource allocation in times of crisis—getting resources to frontline providers

Decisions about where financial resources should go within health to respond to the COVID-19 crisis were seldom led by systematic prioritization exercises. Reports suggest that most countries shifted financial and human resources towards the COVID-19 response at the expense of PHC.[16] Yet, it is particularly crucial, in times of crisis, to ensure that resources reach frontline facilities and community-level public health providers and advocates.[13] Aside from the political and technical decisions driving resource allocation, there is a need for the often rigid PFM systems to perform better, be more flexible, and ensure that resources reach frontline providers.

PFM tools have been adapted during emergency contexts to allow faster and more responsive allocation of resources. Emergency spending provisions, for example, allow spending in excess of budgeted amounts, temporarily lifting budget caps to allow providers to receive additional budget allocations[12]. Expenditure reprioritization through reallocations and virements which accommodated the additional financial requirements; and the use of supplementary budgets and external grants also help address emergency needs while maintaining core PHC services.[17] In China for example, the central government allowed advance appropriation and fast-track payment to meet spending needs.[13] In France, a fast-track expenditure authorization procedure was adopted and a step in the spending authorization was removed to accelerate the release of funds.[13] In Zimbabwe, funds from results-based budgeting were re-channelled towards COVID-19 health services.[12] Extending this type of flexibility beyond crisis situations may be appropriate. Whatever the crisis, ensuring a balance between the flexibility needed to respond rapidly to shocks and the financial controls necessary to enable monitoring of budget allocations will be crucial.[18]

Appendix 2. Estimating PHC expenditure

WHO reports data on total PHC spending on 98 countries for 2018 and government spending on PHC on 61 of these. We observed that the WHO database did not provide data on government spending on PHC for all OECD countries, despite the availability of health expenditure by financial source and health function from the OECD database. In order to compare spending levels in high income countries with those in low- and middle-income countries, we extracted the components of PHC expenditure by financial source from the OECD database and reconstructed total PHC spending and government spending on PHC per capita for all OECD countries. We followed the broad WHO definition of PHC spending with the single modification of excluding administration cost from PHC expenditure. We used the WHO exchange rate and population data to convert the raw data from local currency to current USD values in 2018. With the addition of reconstructed government spending on PHC per capita from the OECD database following the WHO definition, the total number of countries providing data on government spending on PHC increased to 90 countries.

Table A1 Panel of PHC dataset and source of data

	Country	Total PHC spending per capita in US$ (2018)	Government spending on PHC per capita in US$ (2018)	Data Source*
1	Afghanistan	28.18	1.27	WHO
2	Armenia	202.62	22.08	WHO
3	Australia	1906.34	1089.10	OECD
4	Austria	1959.49	1282.69	OECD
5	Barbados	748.77	230.39	WHO
6	Belarus	155.08	88.54	WHO
7	Belgium	2034.36	1385.91	OECD
8	Bhutan	46.94	36.73	WHO
9	Bosnia and Herzegovina	226.97	.	WHO
10	Botswana	265.90	228.18	WHO
11	Bulgaria	327.46	124.35	OECD
12	Burkina Faso	31.29	13.09	WHO
13	Cabo Verde	95.37	64.40	WHO
14	Cambodia	62.86	12.19	WHO
15	Canada	2396.59	1351.25	OECD
16	Central African Republic	49.68	1.62	WHO
17	Congo, Dem. Rep.	10.18	.	WHO
18	Congo, Rep.	27.01	8.19	WHO
19	Costa Rica	296.48	155.05	OECD
20	Côte d'Ivoire	57.80	16.60	WHO
21	Croatia	384.73	288.86	OECD
22	Cyprus	796.22	244.33	OECD
23	Czech Republic	586.43	398.77	OECD
24	Denmark	2364.61	1752.73	OECD
25	Dominican Republic	191.37	68.01	WHO

**26**	Egypt	57.95	17.58	WHO
**27**	Estonia	682.58	420.61	OECD
**28**	Eswatini	149.88	37.84	WHO
**29**	Ethiopia	19.86	.	WHO
**30**	Fiji	132.61	87.91	WHO
**31**	Finland	2065.50	1354.27	OECD
**32**	Gabon	120.25	61.75	WHO
**33**	Georgia	114.47	14.85	WHO
**34**	Germany	2619.02	2117.90	OECD
**35**	Ghana	56.84	.	WHO
**36**	Guinea	28.85	4.69	WHO
**37**	Guyana	224.27	140.77	WHO
**38**	Haiti	39.03	5.86	WHO
**39**	Hungary	432.53	237.32	OECD
**40**	Iceland	2297.23	1484.99	OECD
**41**	India	32.18	10.73	WHO
**42**	Japan	2175.38	1722.55	OECD
**43**	Jordan	164.75	60.65	WHO
**44**	Kazakhstan	148.35	70.31	WHO
**45**	Kenya	64.12	25.98	WHO
**46**	Korea, Rep.	1439.45	813.01	OECD
**47**	Lao PDR	44.85	17.15	WHO
**48**	Latvia	438.63	205.43	OECD
**49**	Liberia	30.74	7.41	WHO
**50**	Lithuania	594.51	302.60	OECD
**51**	Luxembourg	2360.34	1810.33	OECD
**52**	Malawi	20.65	3.88	WHO
**53**	Mali	29.34	6.89	WHO
**54**	Malta	1719.95	873.14	OECD
**55**	Mauritania	32.43	14.17	WHO
**56**	Mauritius	301.38	84.36	WHO
**57**	Moldova	114.28	41.70	WHO
**58**	Mozambique	19.32	4.14	WHO
**59**	Myanmar	39.99	3.94	WHO
**60**	Namibia	272.64	139.70	WHO
**61**	Nepal	38.38	9.89	WHO
**62**	Netherlands	1718.86	1217.75	OECD
**63**	Niger	19.92	6.81	WHO
**64**	Nigeria	57.89	4.93	WHO
**65**	North Macedonia	153.75	68.59	WHO
**66**	Norway	3184.07	2352.21	OECD
**67**	Pakistan	23.96	7.33	WHO
**68**	Paraguay	183.53	86.90	WHO
**69**	Poland	461.18	272.13	OECD

**70**	Romania	237.78	140.42	OECD
**71**	Russian Federation	255.80	117.30	OECD
**72**	Samoa	116.93	69.26	WHO
**73**	Sao Tome and Principe	87.83	25.75	WHO
**74**	Senegal	38.80	7.66	WHO
**75**	Seychelles	485.98	331.42	WHO
**76**	Slovakia	612.37	443.51	OECD
**77**	Slovenia	925.76	555.62	OECD
**78**	South Sudan	18.71	1.33	WHO
**79**	Spain	1075.02	620.11	OECD
**80**	Sri Lanka	60.08	12.61	WHO
**81**	St. Kitts and Nevis	607.96	239.50	WHO
**82**	Suriname	219.85	127.10	WHO
**83**	Sweden	2269.67	1642.81	OECD
**84**	Switzerland	3922.55	2174.22	OECD
**85**	Tajikistan	27.46	5.78	WHO
**86**	Tanzania	16.68	7.03	WHO
**87**	Timor-Leste	60.01	35.04	WHO
**88**	Togo	28.52	5.12	WHO
**89**	Tonga	100.07	69.15	WHO
**90**	Trinidad and Tobago	706.03	324.92	WHO
**91**	Tunisia	109.76	62.63	WHO
**92**	Uganda	25.56	4.45	WHO
**93**	Uruguay	636.49	.	WHO
**94**	Uzbekistan	34.23	7.42	WHO
**95**	Zambia	59.66	18.37	WHO
	Total	95	90	

*WHO database: https://apps.who.int/nha/database/Select/Indicators/en
OECD database: https://www.oecd-ilibrary.org/social-issues-migration-health/data/oecd-health-statistics/system-of-health-accounts-health-expenditure-by-function_data-00349-en

Table A2 Comparison of total PHC spending from WHO and OECD database

Countries	*PHC per capita (2018, current US$) from OECD*	*PHC per capita (2018, current US$) from WHO*
Australia	1906.3	.
Austria	1959.5	1959.5
Belgium	2034.4	1975.9
Bulgaria	327.5	327.5
Canada	2396.6	2405.5
Chile	.	.
Colombia	.	.
Costa Rica	296.5	297.3
Croatia	384.7	382.3

Cyprus	796.2	796.0
Czech Republic	586.4	586.4
Denmark	2364.6	2364.6
Estonia	682.6	682.6
Finland	2065.5	2065.5
France	1487.8	2015.0
Germany	2619.0	2619.0
Greece	494.0	706.4
Hungary	432.5	431.8
Iceland	2297.2	2297.2
Ireland	1494.5	2584.5
Israel	.	.
Italy	783.8	.
Japan	2175.4	.
Latvia	438.6	434.3
Lithuania	594.5	594.5
Luxembourg	2360.3	2360.3
Malta	1719.9	1719.9
Mexico	228.2	228.2
Netherlands	1718.9	1718.9
New Zealand	.	.
Norway	3184.1	3184.1
Poland	461.2	459.6
Portugal	445.6	1279.1
Republic of Korea	1439.4	1439.4
Romania	237.8	237.2
Russian Federation	255.8	255.8
Slovakia	612.4	612.4
Slovenia	925.8	925.8
Spain	1075.0	1075.0
Sweden	2269.7	.
Switzerland	3922.6	3922.6
Turkey	.	.
United Kingdom	1166.1	2279.0
United States	2900.6	.

As a validity check, we compared the total PHC spending from the WHO database with our reconstructed figures (see Table A2). Overall, both figures are reasonably similar with minimum deviation. On the basis of this comparison, however, we excluded 6 countries and included a further 3, providing data on 95 countries in total.

- WHO figures on total spending on PHC for France, Greece, Ireland, Portugal, and the UK are significantly higher than our reconstructed PHC spending from the OECD database (See table A2). In these countries, outpatient care expenditure is not broken down into its components such that expenditure on 'Specialist outpatient care' is included, whereas according to the WHO definition it should be excluded

from PHC. Given that on average, Specialist care accounts for around 40% of overall outpatient care spending, including the spending will inflate PHC spending for these countries. We decided to exclude these countries from our analysis

- We also excluded Mexico because the estimate of spending on medical goods only includes private spending. Thus, there is an underestimation of total PHC spending and particularly public spending on PHC
- We included 2017 data on Australia and Japan due to the different financial year accounting. We also included data for Sweden, which is not present in the WHO database for an unknown reason.
- The WHO database provided information on **total PHC spending** for 98 countries. Our final sample was 95 countries after excluding six countries (France, Greece, Ireland, Portugal, the UK and Mexico) and including three countries (Australia, Japan, and Sweden).

Descriptive analysis of PHC expenditure

Figure A1: PHC spending per capita

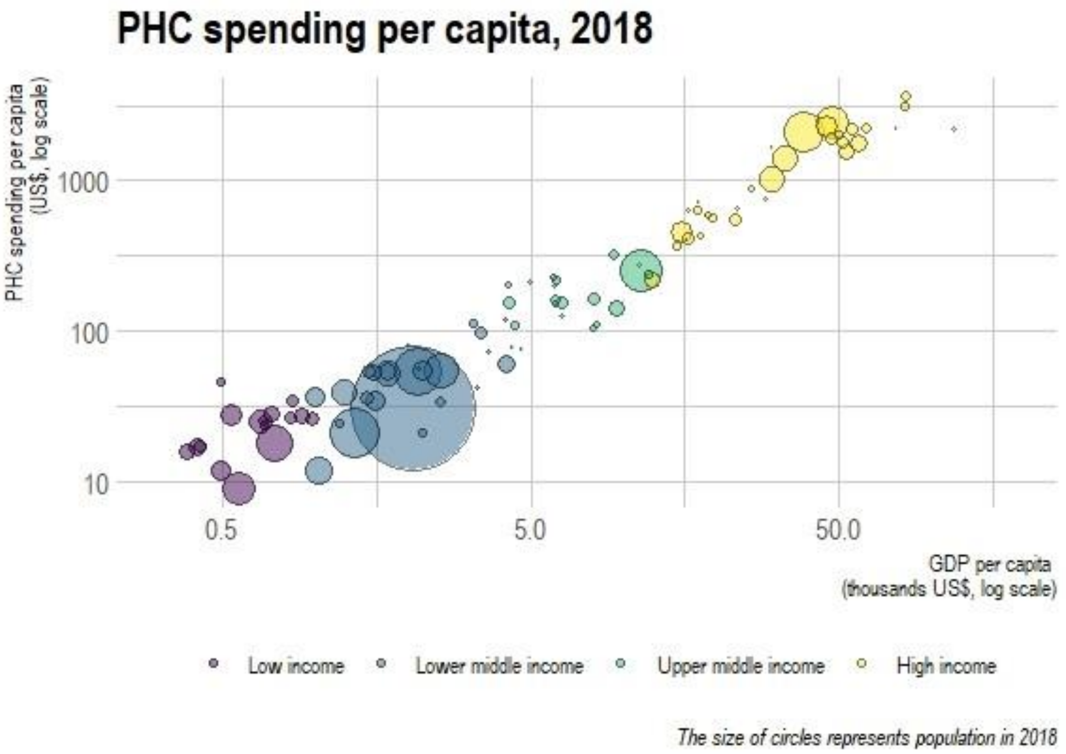

Note: In this and subsequent scatter plots we use the size of the circle to represent population. This provides a sense of the number of people who are exposed to this level of expenditure.

Figure A2: PHC spending as a share of Current Health Expenditure (2018)

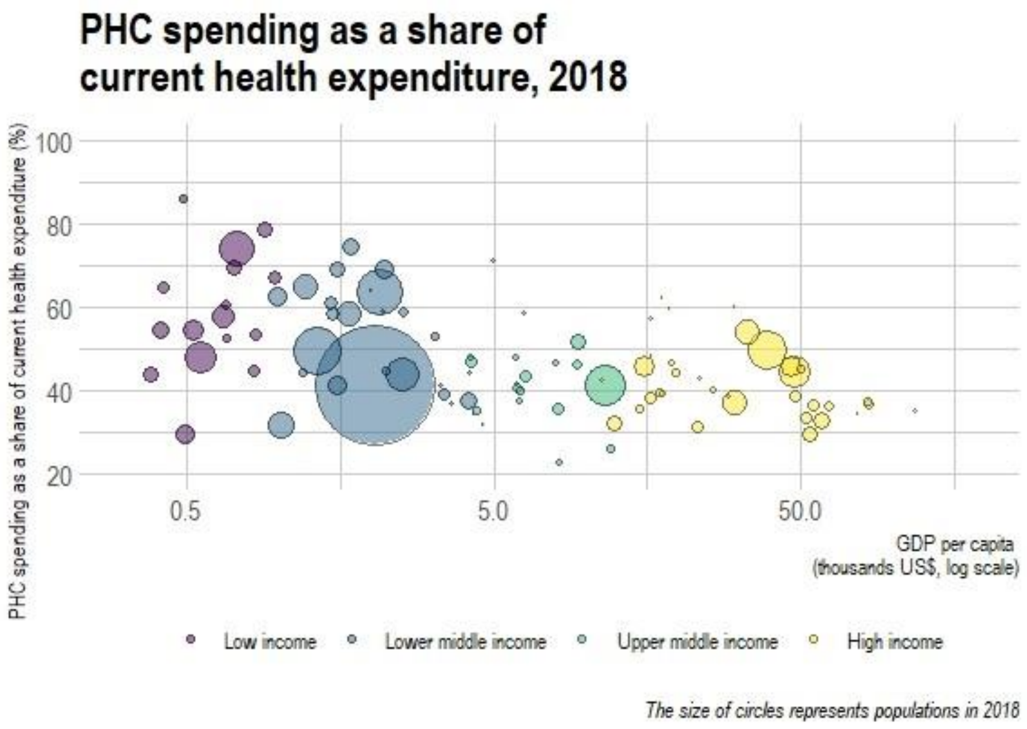

Figure A3: Government spending on PHC as a share of government spending on health (2018)

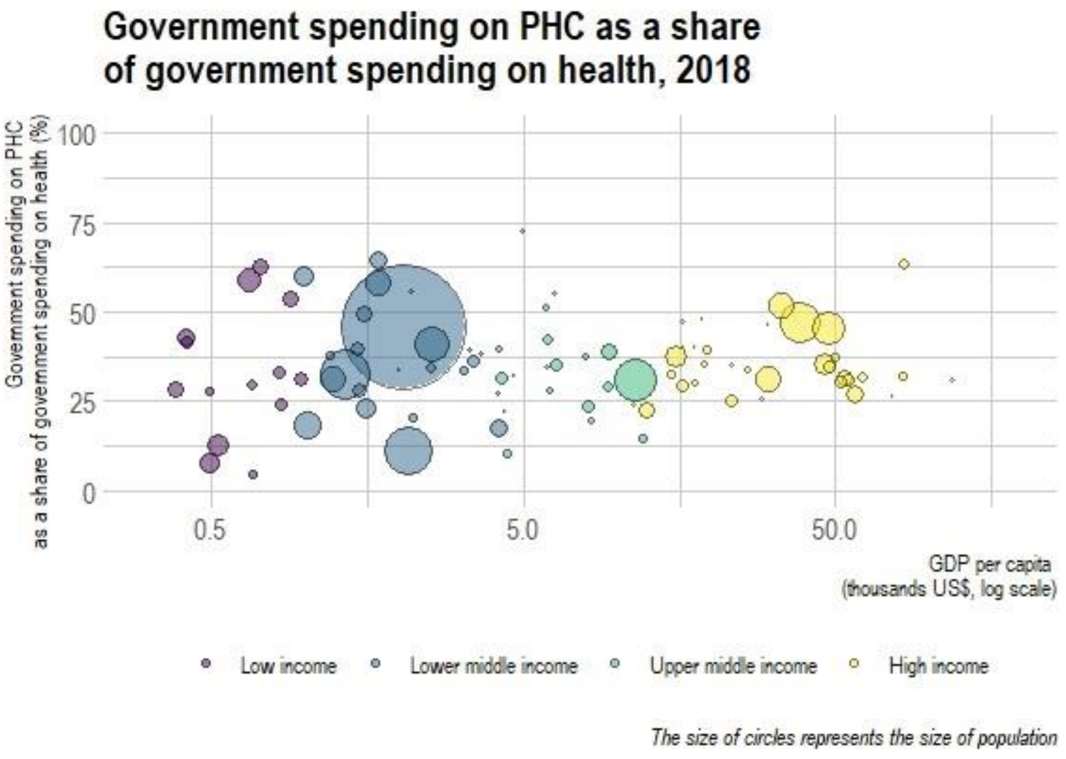

Figure A4: Total PHC spending by financial source (2018)

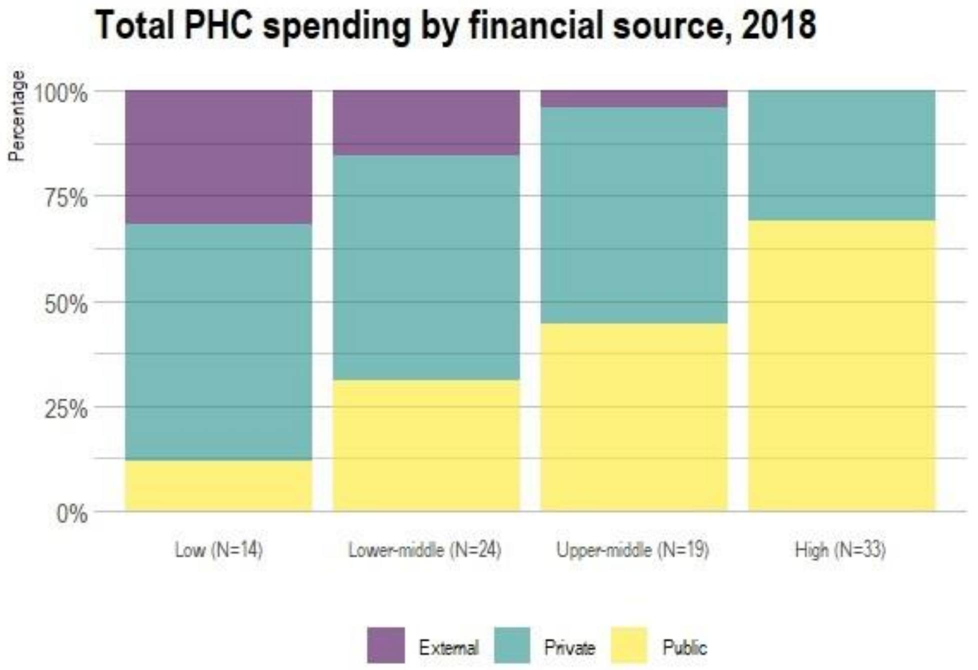

Figure A5: External spending on PHC as share of total PHC spending (2018)

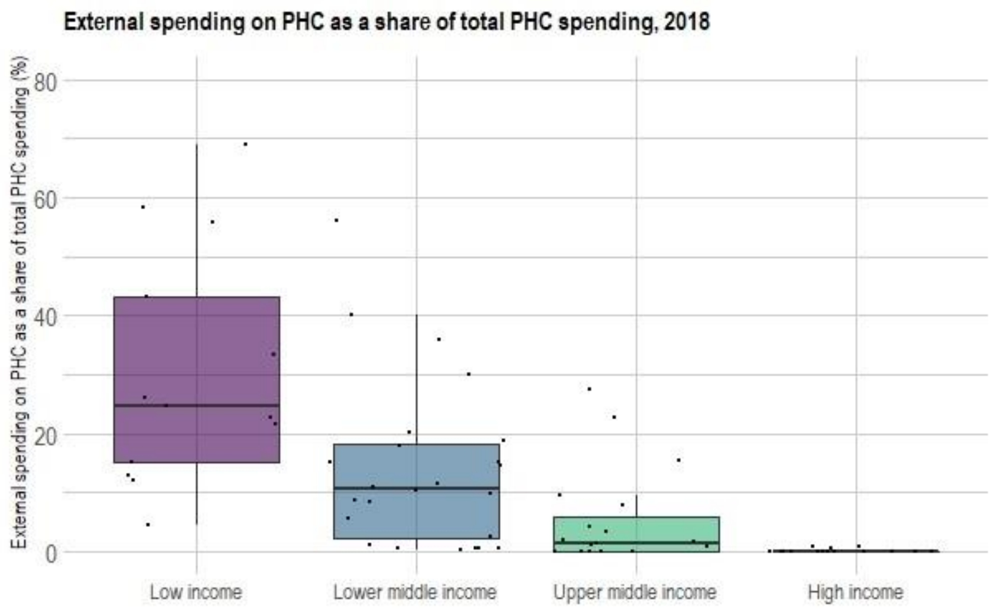

Note: Boxplots show the median (dark horizontal line) the interquartile range (25th–75th percentile, coloured box), and minimum and maximum values (the vertical lines extending from the boxes). Each dot represents one country.

Figure A6: Relationship between domestic general government health expenditure and GDP in LMICs

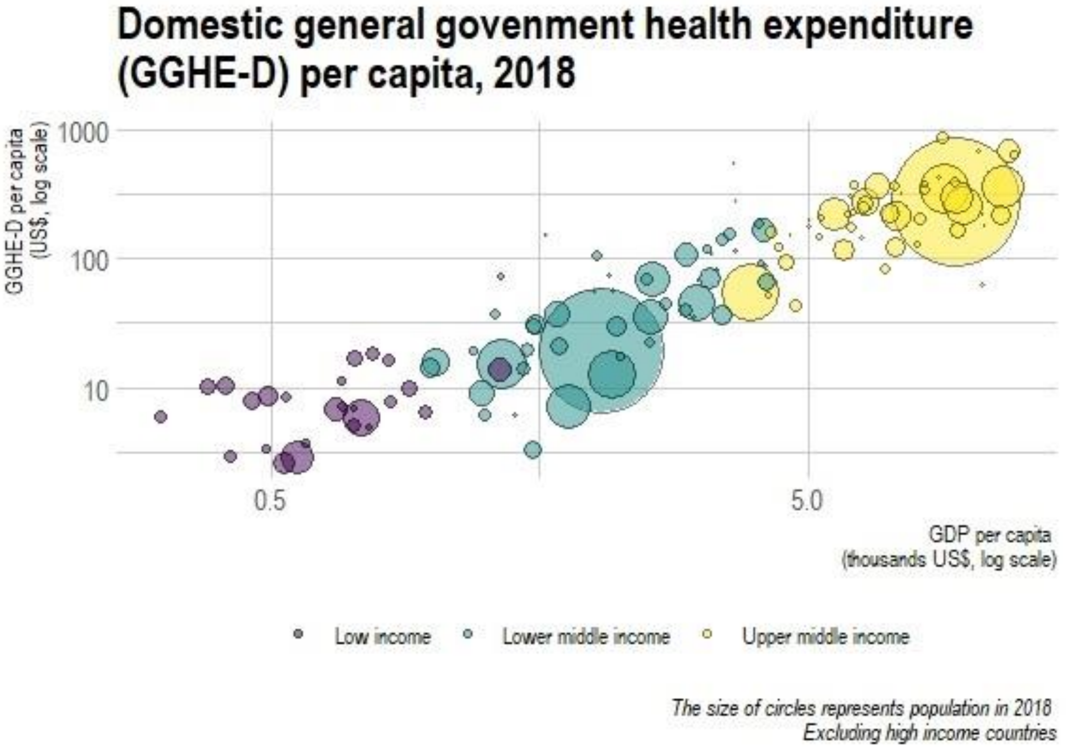

Appendix 3. Survey of PHC Organisation and Provider Payment

a. Study design and participants

The aim of the survey was to understand how PHC providers are organised and paid in LMICs. We conducted a cross-sectional survey of countries between 5th May 2021 and 27th June 2021. Participants were health financing experts in each country, identified by LSHTM, World Bank, Results for Development and WHO. The participants were from a range of organisations, including country governments, universities, consultancy organisations, World Bank country offices and WHO country offices. The study received ethics clearance from LSHTM (ref number 22645) and participants were required to provide informed consent before proceeding with the survey.

b. Questionnaire development

The survey questionnaire was developed by LSHTM (Timothy Powell-Jackson, Darius Erlangga, Henry Cust, Kara Hanson) and World Bank (Alix Michele Beith, Christoph Kurowski), with input from Cheryl Cashin, Agnes Munyua and Martin Roland. The questionnaire comprised three sections. The first section asked questions on how PHC providers in the public, for-profit and not-for-profit sectors are organised in the country. The second section was on how community health workers supporting the public sector are paid. The third section examined how PHC providers in each sector are paid by each of the following payers: government payers; social health insurance payers; community-based health insurance payers; private health insurance payers; donor and NGO payers; and households (i.e. user fees). The questionnaire drew on existing tools, including the OECD Survey of Health Systems Characteristics. It is available on request.

We designed the questionnaire to contain substantial guidance such that it could be self-completed by participants. We piloted the questionnaire in four countries, before finalising the tool. The survey questions were in the English language. The questionnaire was designed in and delivered through "Online Surveys", which allowed us to programme skips and checks for data validation. The questionnaire took approximately one hour to complete. A key challenge was how to deal with sub-national variation in a country. For example, in some countries in which the local government is the payer of public PHC providers, different payment methods may be used across different states or provinces. It would have been too onerous to capture such variation; we therefore asked respondents to answer questions according to the national picture in terms of what generally happens.

c. Definitions: providers and payers

For the purposes of this survey, we defined "primary health care" as a set of essential services – preventative, promotive, and curative – that is provided to individuals and populations. We took "health care provider" to mean a licensed physical primary health care provider "unit" or "entity" that offers PHC services, such as a health centre. While in the majority of cases the "entity" includes more than one individual working there (i.e., all health care workers in a public or private PHC health centre), the "entity" could be just one individual, such as with a solo practice. We also noted that the PHC provider can be part of a larger outpatient unit (i.e., a polyclinic model).

This meant the survey excluded: health care providers that *only* deliver specialized outpatient services, dental services, eye care services, long-term and palliative care; health care providers that are hospitals; and standalone pharmacists and drug shops. While the Commission took a much broader definition of PHC, this narrow definition of PHC provider was required to make the survey feasible and ensure response rates were not woefully low. The survey contained questions on PHC providers in public, for-profit and non-profit sectors. Public PHC providers were those that are publicly-owned and operate under public law. For-profit PHC providers were those that are privately-owned, operate under private law and are established as independent legal entities with for-profit status. Non-profit PHC health care providers were those that are privately-owned, operate under private law and are established as independent legal entities with non-profit status.

We defined a payer as an organisation that transfers resources directly to a PHC provider. By resources, we meant the transfer of money or inputs (in-kind), such as medicines or equipment, directly to a PHC provider. An individual patient can also be a payer when paying for health care out-of-pocket. Payers of PHC services could be one or a combination of three main categories:

- Public payers – Government (Ministry of Health, Other Ministries, Local Government), and Social Health Insurance Agency.
- Private payers – Voluntary private health insurance, community-based health insurance (both of which pay for covered individuals), and individuals who pay out of pocket.
- Donors – Multi- and bi-lateral development agencies, as well as private donors and philanthropy.

We defined the payment method as the mechanism used to transfer resources from payer(s) to PHC health care providers. The payer can directly pay the salaries of the health workers. The payer can also transfer money to the health care provider as a: line item budget, global budget, capitation payment, case-based payment, fee-for-service reimbursement, or pay-for-performance bonus. The payer can also give inputs, such as medicines, directly to the health care provider. As is clear from this list, the survey did not capture some of less-common and newest payment methods, such as those that have been used to incentivise value-based care and bundled payments.

d. Data collection

The questionnaire was sent out in a personal email to health financing experts identified through the networks of LSHTM, World Bank, Results for Development and WHO. The survey could be completed in two ways – either through self-completion or through a videoconference interview. In the case of the former, the study team reviewed responses and sought clarifications from the interviewees. While there was no formal requirement for respondents to get government input or approval for their responses, this did happen in some countries as a mean of validation.

In total, the survey was sent out to 107 LMICs (see Table A3). There were 75 responses, indicating a response rate of 70%. The breakdown of responses by World Bank income region was: 22 low-income countries; 22 lower middle-income countries; 31 upper middle-income countries. The population in each region covered by the survey responses were:

83.8% of low-income countries; 86.3% of lower middle-income countries; and 97.1% of upper middle-income countries. We performed a descriptive analysis, disaggregating the data by World Bank income groups. We did not weight the data by country population

Table A3. Countries with survey responses

**Low-income (22)**	**Lower middle-income (22)**	**Upper middle-income (31)**
Afghanistan	Bangladesh	Albania
Burkina Faso	Benin	Argentina
Burundi	Bhutan	Armenia
Chad	Cambodia	Azerbaijan
Democratic Republic of Congo	Cameroon	Bosnia and Herzegovina
Ethiopia	Egypt	Brazil
Guinea	Ghana	Bulgaria
Guinea-Bissau	India	China
Haiti	Kenya	Colombia
Liberia	Laos	Costa Rica
Madagascar	Moldova	Ecuador
Malawi	Mongolia	Fiji
Mali	Morocco	Georgia
Mozambique	Nepal	Guatemala
Niger	Nigeria	Indonesia
Sierra Leone	Pakistan	Iran
Sudan	Papua New Guinea	Iraq
Sudan, South	Philippines	Jamaica
Tajikistan	Sri Lanka	Kazakhstan
The Gambia	Tanzania	Kosovo
Togo	Tunisia	Lebanon
Uganda	Ukraine	Malaysia
		Mexico
		North Macedonia
		Peru
		Russia
		Saint Vincent and the Grenadines
		South Africa
		Thailand
		Turkey
		Venezuela

Appendix 4. Digital technology and health financing

Virtual pooling with digital technologies

Virtual pooling enables unpooled resources to be managed in a coordinated manner by a strategic purchaser. Virtual pools are made possible through inter-operable digital information systems that integrate or aggregate data analysis. They may allow for pool-merging as in Burundi, improved risk equalization or risk adjustment, and harmonization of benefits and payment rates across different schemes.[19]

However, the use of digital technology for pooling purposes needs to align with UHC objectives. Digital technologies may hamper pooling efforts by increasing fragmentation (when, for example, multiple start-ups and smaller targeted health insurance schemes operate independently). Digital records can also facilitate more precise differentiation among risk profiles, which could lead to the exclusion of high-risk individuals from pools. Digital banking can also discourage cross-population pooling by enabling the use of mobile wallets for individual medical savings accounts.

Digital technologies and revenue mobilization

The evidence on the potential of digital technologies to support the mobilisation of revenue for health is limited.[20] Digital technologies have supported voluntary mobilisation in Rwanda, where a large private insurer successfully recruited members working in the informal sector by enabling the collection of premiums via mobile phone. In Kenya, a micro-insurance scheme relied on 'mobile money' and 'mobile wallet' payment services to allow members to pay premiums in instalments. Digital technologies also enable diaspora communities to contribute resources to home communities for health costs, as presented by experts during the Commission's roundtable discussion. However, few examples have been documented. And thus far, they focus on voluntary rather than compulsory contributions, limiting their potential to support the UHC objectives of cross-subsidies in contributions.

Digital technologies to support provider payment and strategic purchasing

The digitalisation of information systems in support of financing health is ongoing across LMICs and has so far focused more on purchasing arrangements than resource mobilisation or pooling.[20] However, whilst projects supporting the use of digital technologies in provider payment (and more broadly in purchasing PHC) exist, documentation of these examples is limited, and few have been evaluated.[19] The Commission's roundtable discussion on digital technologies for PHC financing highlighted that this dearth of evidence may not reflect either the dynamism of the sector, nor the enthusiasm for the revolution digital technologies may represent for purchasing. Experts argued that digital technology on purchasing hold great promise for improving efficiency as it streamlines monitoring (generating better metrics on, for example, the number of patients seen, prices of services, types of purchasers, or identification of ghost workers). The inter-operability of these data sources *could* reduce their fragmentation and improve the interaction between purchasers, providers and patients.

However, as mentioned in section 2, the digital revolution still needs to be fully harnessed and directed to ensure it promotes objectives aligned with the desirable attributes of people-centred financing for PHC. For example, the difficulty of linking different data sets across purchasers may lead to further fragmentation rather than harmonisation of purchasing arrangements as the interoperability of datasets represents a major challenge. Mobile health wallets can encourage individual saving and simplify payment, but they work against the need to harmonise funding flows and align incentives for PHC providers. Careful evaluation and research are warranted to ensure that the digital revolution supports UHC.

Methodology: Expert roundtable discussion on digital technologies for financing PHC

a. Objective
The digital technology field is fast moving, and its progress is difficult to grasp through a conventional literature review. Through the roundtable discussion, we aimed to understand the current interaction between digital technologies and health financing functions by capturing the perspective of actors involved in this field.

b. Selection of respondents

- expertise in both digital technology and health financing;
- representatives across academia, donors, implementers, at global and national levels

The roundtable gathered an academic (John Hopkins University), 5 representatives from different donor agencies (OECD, World Bank, BMGF, WHO and USAID), and 6 implementers (BlueSquare, Digital Alliance, IntraHealth, PATH and two LMIC MoH representatives). The chair was Kara Hanson (LSHTM).

c. Method
We used a consensus building round-robin interview technique, following a topic guide.

d. Transcription and analysis
The discussion was recorded in Zoom and transcribed verbatim using Otter Artificial Intelligence. A coding tree was developed to analyse the transcript of discussion. NVIVO was used to extract information as per coding tree.

Appendix 5. Review of inefficiencies in health

We searched Medline, Embase, Global Health, Econlit, Africa-Wide information, Web of Science Core Collection and SciELO citation index. We identified additional studies through references of identified articles and suggestions from key experts in health financing. We restricted the search to articles in English, published between 1st Jan 2000 and 31st Jan 2021. We used keywords including "health financing," "efficiency," "savings, "resource allocation," cost effective," "inefficiency," and "value for money" (see Supplemental Table C1 for a complete search strategy). We included papers which addressed inefficiencies in medicines procurement and use, leakages (i.e. waste, corruption and fraud), human resources for health and administrative costs of fragmented health financing schemes. We examined studies that met either of the following two criteria:

- Studies that explored the impact of reforms to health financing functions (i.e., revenue mobilisation, pooling, resource allocation, and purchasing) on the inefficiency of interest. These studies had to report the baseline information related to the inefficiency measures or/and the quantified cost savings associated with the implementation of the reforms.
- Studies that assessed the feasibility of health financing reforms on the inefficiency of interest

We excluded cost-effectiveness studies of a health intervention without any consideration of health financing reforms. Since we were interested in the cost savings generated through tackling the inefficiencies, we excluded studies that only reported efficiency gains in terms of health outcomes. Other costing studies without any reference to health reforms and cost savings were also excluded.

Two reviewers screened the title and abstracts. Articles meeting inclusion criteria and obtainable as full texts were reviewed in detail. Data from individual studies were extracted into Microsoft Excel spreadsheet for analysis. We analysed two groups of articles: the first was on the impact of health financing reforms on inefficiencies and the second on the feasibility and the timing of the reforms. Due to the heterogeneity of inefficiency measures, we reported results with a descriptive approach and narrative synthesis.

The literature search in scientific databases resulted in an initial identification of 4412 publications. Additional articles were found by reference tracking and consultation with key experts. Based on the application of the inclusion criteria to the title and abstracts, 17 articles were retrieved for narrative analysis (see Figure S1 for detail on study selection process).

Table A4. Types of inefficiencies and associated reforms

Inefficiency	Reforms implemented to address identified inefficiencies
High price of pharmaceuticals including high mark-ups, high prices in procurement, variation in price of same medicine and high price due to prescribing preferences of providers and limited competition among distributors.	Price controls, pre-approval for expensive drugs; central, competitive, and bulk procurement, volume controls, purchaser-provider split, splitting tenders, rationalizing pharmaceutical care – the only pharmaceutical in a reference group is the one with the cheapest price.<br>Co-payment for prescription drugs, budget, or quota restrictions for physicians.<br>Essential medicines list (that used generics to the maximum), better monitoring of medicine quality and safety, improving provider and patient knowledge about generics.<br>Establishment of an independent regulatory agency which: banned hiring officials linked to pharmaceutical companies, banned royalties to physicians who promoted products, ensured regulation of advertising, mandated physicians to prescribe by active ingredients and not brand, established price ceiling for pharmaceuticals.
Inappropriate/ineffective/overuse/counterfeit drugs.	Province-based competitive-bidding system, bulk procurement for provinces, essential medicine list, price ceilings for drugs on the list.<br>Zero-profit drug policy.<br>Local social health insurance programmes to provide higher reimbursement for listed drugs.
High public expenditure on pharmaceuticals.	Purchaser-provider split, improving PHC, expanding family medicine, increased financial and administrative autonomy for public hospitals, performance-based financing (PBF) and capitation payments to family physicians.<br>Single sector policy and expenditure programme (under government leadership).
Absenteeism of health workers.<br>Insufficient health workers for PHC, inefficient mix of inputs between capital and labour, long distances to staffed facilities.	PBF, strategic purchasing<br>Creating a new cadre of health extension workers (HEWs).
High overhead costs due to overlapping health financing pools and fragmented systems.	Pooling of health financing schemes.<br>Single national coordinating mechanism, national system for essential medicines procurement.<br>Medical-Loss-Ratio stipulation<br>(Requiring insurers to spend at least 80-85% of premiums on medical claims), defined ceilings and efficiency targets, e-solutions to optimise hospital staff (limiting the use of additional temporary staff), better monitoring of administrative costs.
Inefficiency in purchasing PHC services from providers, inadequate resource allocation to PHC providers.	Contracting PHC services.<br>China - Government increased funding per head by 10% to PHC providers.<br>Zambia - Single sector policy and expenditure programme, under government leadership.

Table A5. Studies with quantitative evidence on reforms to address inefficiencies in health financing

The literature search in scientific databases resulted in an initial identification of 4412 publications. Additional articles were found by reference tracking and consultation with key experts. Based on the application of the inclusion criteria to the title and abstracts, 17 articles were retrieved for narrative analysis (see Figure S1 for detail on study selection process).

**Inefficiency**	**Reforms**	**Impact of reform on health expenditure**	**Studies**
High price of pharmaceuticals including high mark-ups, high prices in procurement, variation in price of same medicine and high price due to prescribing preferences of providers and limited competition among distributors.	• Price controls, pre-approval for expensive drugs; central, competitive, and bulk procurement, volume controls, purchaser-provider split, splitting tenders, rationalizing pharmaceutical care – the only pharmaceutical in a reference group is the one with the cheapest price.<br>• Co-payment for prescription drugs, budget, or quota restrictions for physicians.<br>• Essential medicines list (that used generics to the maximum), better monitoring of medicine quality and safety, improving provider and patient knowledge about generics.<br>• Establishment of an independent regulatory agency which: banned hiring officials linked to pharmaceutical companies, banned royalties to physicians who promoted products, ensured regulation of advertising, mandated physicians to prescribe by active ingredients and not brand, established price ceiling for pharmaceuticals.	• Montenegro and Macedonia - ~ 30% ↓ in pharmaceutical expenditures.<br>• Nepal - Could yield significant savings as prices of local procurement are ~300% of central procurement.<br>• Nepal - Could save >18% of value of pharmaceuticals.<br>• Cost savings from switching from specific brand name to generic medicines = 51% in Pakistan and 53% in China, between 4% and 23% in Austria, and between 11% and 73% in 17 LMICs.<br>• China - Bulk purchasing resulted in 25% ↓ in price of essential medicines.<br>• China - Price of 29 generic drugs had ↓ by 5.3% in the public sector and ↓ 4.7% in private pharmacies.<br>• El Salvador- Average price reductions in pharmaceuticals of 20% to 25%.<br>• Mexico - Cumulative savings of $2.8 billion between 2007 and 2010.<br>• Greece - 10% and 20% price reduction for pharmaceuticals and selected medical devices, respectively.<br>• New Zealand - Savings of ~ NZD 5.1 billion from 2005 to 2015.<br>• Denmark and Norway - Savings from 30% to >60% compared to average wholesale prices in some neighbouring countries.	Bredenkamp and Gragnolati (2007)[2], Mitenbergs et al (2012)[3], Okem and Cakar (2015)[4], Belay and Tandon (2015)[5], World Bank (2017)[8], Yip and Hafez (2015)[9], OECD (2017)[10]

Inappropriate/ineffective/overuse/counterfeit drugs.	• Province-based competitive-bidding system, bulk procurement for provinces, essential medicine list, price ceilings for drugs on the list.<br>• Zero-profit drug policy.<br>• Local social health insurance programmes to provide higher reimbursement for listed drugs.	N/A	Yip et al (2012)[6], World Bank (2017)[8],
High public expenditure on pharmaceuticals.	• Purchaser-provider split, improving PHC, expanding family medicine, increased financial and administrative autonomy for public hospitals, performance-based financing (PBF) and capitation payments to family physicians.<br>• Single sector policy and expenditure programme (under government leadership).	• Turkey - Share of public expenditure on pharmaceuticals in GDP decreased after 2003 until 2009: 2003, 1.5%; 2004, 1.4%; 2005-2007, 1.3%; 2008, 1.4%; 2009, 1.6%.<br>• Zambia - ↓ in government funds to drugs from ~ 21.1% between 1990 and 1992, to 2.7% in 2005.	Okem and Cakar (2015)[4], Belay and Tandon (2015)[5]
Absenteeism of health workers. Insufficient health workers for PHC, inefficient mix of inputs between capital and labour, long distances to staffed facilities.	• PBF, strategic purchasing<br>• Creating a new cadre of health extension workers (HEWs).	• Nepal - Addressing absenteeism equals increasing government health budget by 1.6%.<br>• Ethiopia - Savings from lower wage bills for HEWs, health officers and emergency surgical officers are $20 million annually	Belay and Tandon (2015)[5], Yip and Hafez (2015)[9]
High overhead costs due to overlapping health financing pools and fragmented systems.	• Pooling of health financing schemes.<br>• Single national coordinating mechanism, national system for essential medicines procurement.<br>• Medical-Loss-Ratio stipulation<br>• (Requiring insurers to spend at least 80-85% of premiums on medical claims), defined ceilings and efficiency targets, e-solutions to optimise hospital staff (limiting the use of additional	• DRC - Reduction in management costs of internationally funded projects, from ~ 28% in 2005 to 9% in 2011 and savings of more than $56 million.<br>• DRC - Greater transparency in planning and budgeting increased operational budgets by 30% in some provinces.<br>• DRC - Reform of the pharmaceutical sector and pooling of resources for regional drug transport resulted in annual savings of > $3.5 million.<br>• RoK - Decreased administrative costs from 7.87% of all National Health Insurance Scheme	Yip and Hafez (2015)[9], World Bank (2017)[8], OECD (2017)[10], Kutzi et al (2010)[11]

	temporary staff), better monitoring of administrative costs.	expenditure to 2.38% between 1996 and 2008.<br>• Kyrgyzstan - Share of government health expenditure allocated to PHC increased from 26.4% to 37.9%.<br>• USA - The share of non-medical overhead costs in net premiums decreased, resulting in accumulated savings of $3.7 billion by 2013.	
Inefficiency in purchasing PHC services from providers, inadequate resource allocation to PHC providers.	• Contracting PHC services.<br>• China - Government increased funding per head by 10% to PHC providers.<br>• Zambia - Single sector policy and expenditure programme, under government leadership.	• Spain - In 1996, average primary care contract purchased health services at a cost that if reduced to 92% of its observed level, would not affect level of output<br>• Zambia - Pre-reform, an average of 31% of the total government health budget was allocated to provinces and districts compared with an average of 55% between 1996 and 2005.	Puig-Junoy and Ortun (2003)[1], Yip et al (2012)[6], Chansa et al (2008)[7]

Table A6. Search strategy

**1**	((financ* or fiscal* or funding or revenue* or budget* or insurance or tax or taxation or resource* allocat* or allocat* resouce* or purchas*) adj3 (efficien* or inefficien* or optimi?at* or optimize or saving or save or "value for money" or cost* effective* or over utili?at* or under utili?at*)).ti,ab.
**2**	healthcare financing/ or financing, government/ or Universal health insurance/ or Insurance, health/ or Community-based health insurance/ or taxes/ or resource allocation/
**3**	efficiency/
**4**	2 and 3
**5**	1 or 4
**6**	(health* or healthcare*)
**7**	5 and 6
**8**	remove duplicates from 7

Figure A6: A study flow diagram depicting study selection process

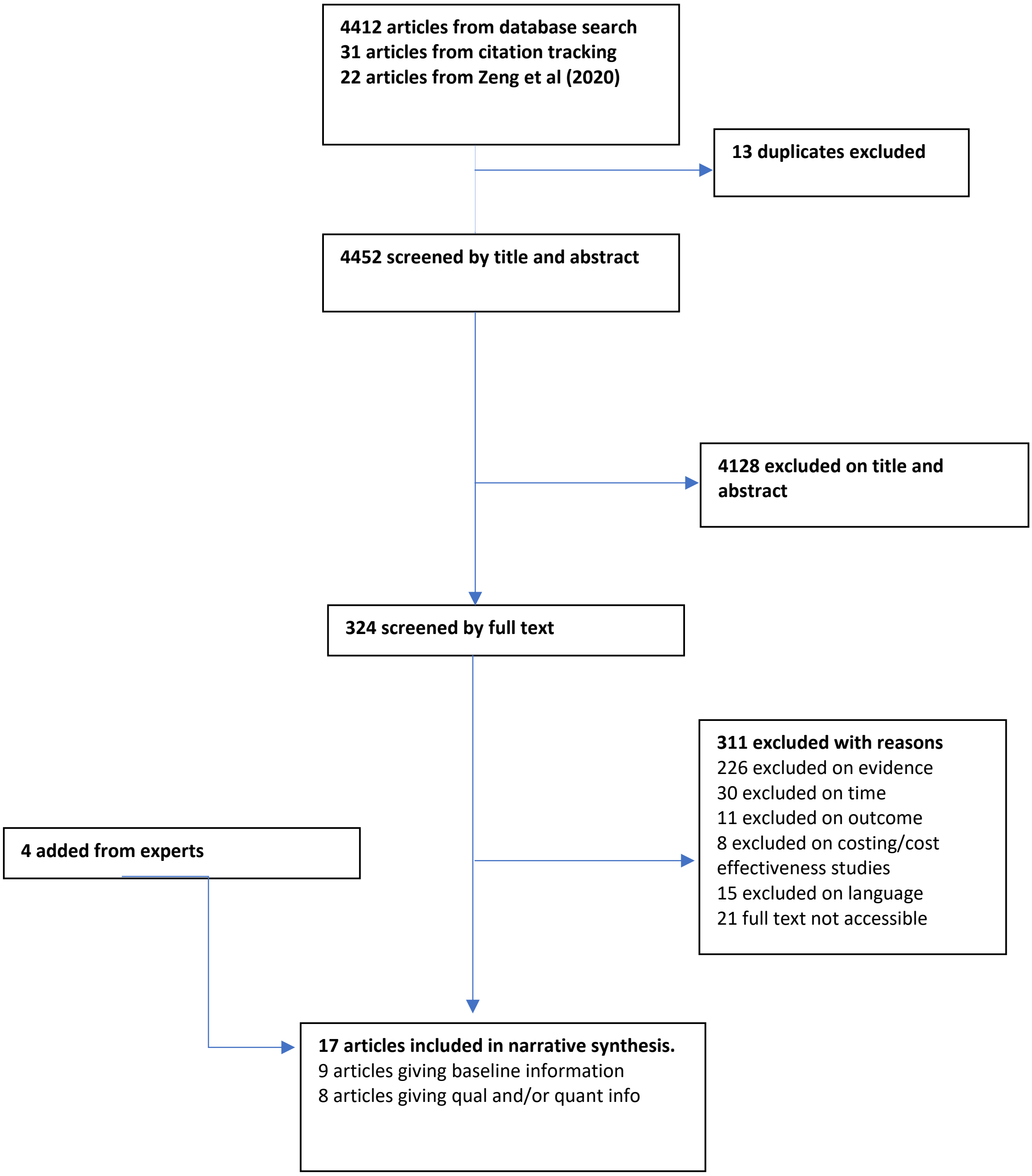

Appendix 6. Political Economy Analysis on Financing PHC

Thailand's multi-actor coalition supports stronger PHC financing

In Thailand, early efforts (1970-2000) to enhance PHC and pave the way for UHC were supported by the bureaucratic elite and technocrats (in both military and civilian governments) in the face of weak political parties.[21] After 2000, a group of policy champions in Thailand, mostly MOPH technocrats driven by a pro-poor ideology and rural health backgrounds, acted as policy entrepreneurs.[22] Meanwhile, the power of economic elites increased, either directly or through their influence on political parties and government, culminating in the landslide victory of the Thai Rak party in 2001. The economic elite continued to support populist policies and advocate for the rights and interests of poorer and rural populations to obtain political advantage. This broad support led to the political decision to put UHC on the election campaign agenda in 2001. It was enacted later that year.

This process was aligned with civil society advocacy. A prominent example of such economic elites was the Rural Doctors Society (formed in 1978, building on the democratization movement of the early 1970s) that sought to strengthen grassroots- and district-level PHC, promote professional solidarity and encourage holistic approaches to health.[23] It had technical roles (developing district hospital management guidelines), but also worked for social recognition for rural doctors and as a watchdog effectively exposing corruption. Other important institutions included the Office of the Primary Health Care Committee (1980), which implemented the Charter for Health Development (1980), and achieved strategic developments at community level such as the introduction of village health volunteers and village health communicators (1981).[22]

Path dependency influences China's PHC financing[24-26]

China's experience shows how important it is to consider path dependence i.e. the understanding that 'current and future states, actions, or decisions depend on the path of previous states, actions, or decisions'[27]- when promoting efforts to strengthen PHC and its financing. In China, funding and delivering PHC nationwide, and particularly benefiting rural populations, has required addressing the long-established system in which hospitals are entrenched as the dominant type of provider that serves the needs of privileged social groups. Hospital and PHC strengthening have followed two different evolutionary paths going through multiple cycles involving ambitious development followed by time lags. Both paths were triggered by critical junctures occurring when windows of opportunity were created by radical changes in the socio-economic context, and were then reinforced or weakened over time. On balance, this led to the persistence of hospital-oriented approaches, which stemmed from the 1800s. Between 1953 and 1965, hospitals were further strengthened by the provision of insurance coverage for inpatient care, the ability of urban populations to pay out-of-pocket, and hospitals' expansion into providing ambulatory services.

This situation was subsequently disrupted by the introduction of government policies to promote and strengthen PHC, with expansion of the deployment of CHWs (called 'barefoot doctors') with basic training, hospital doctors serving in rural areas, and the scale-up of the

Cooperative Medical Scheme, a national rural community health risk-pooling scheme in the later 1960s and the early 1970s.

However, the pre-existing hospital-centric path persisted due to the benefits provided by the (rural) Cooperative Medical Scheme and its provider payment rates being lower than those of the urban insurance schemes, and professionals resisting working in rural areas and more generally, by the pro-urban financing and delivery models established in the 1950s. The country's transition towards a market economy and other reforms (1978–2002) led to a collapse of the agrarian collective economy; this drove rapid urbanisation, emigration of rural-based doctors to urban areas, and increased demand for urban-based secondary and tertiary health care.

The continued bureaucratic orientation of the MOH and the preferences of established professional institutions for hospital-based care have also contributed to the continued dominance of a strong hospital bias in Chinese health policies. The New Cooperative Medical Scheme introduced in 2002 nominally increased resources for PHC, but PHC providers benefited insufficiently as the scheme also prioritized reimbursement for hospitalizations and allowed competition among providers at all levels. These policies both encouraged and were reinforced by patients who frequently bypassed local PHC facilities to seek outpatient care for minor conditions in hospitals and other higher-level facilities. This was despite efforts to upgrade the skills of rural health staff such as the establishment of a general practitioner training programme, which entails training of existing PHC staff in general medicine as well as recruitment and free training for doctors from remote and rural areas and contracting them to work at PHC facilities for a particular term.

The Chinese case illustrates the danger of what is sometimes called 'policy lock-in'. Hospitals attract significant resources and are supported by powerful actors despite policy efforts to foster more equitable PHC. Thus, PHC financing in China continues to be confronted by the path-dependency of institutional structures and processes ranging from the professionalization of health care to the organization and governance of hospitals. Understanding this requires recognition of the diverse professional, bureaucratic and community interests that were aligned around similar objectives, and particularly of the key role of the elites and higher income groups in influencing the balance between primary and secondary care. Path dependence determined the resilience of the hospital model.

Political transition as a critical juncture for PHC financing transformation in Eastern Europe

The dissolution of the Soviet Union and the political transitions of 1989-91 led to significant economic, social and political changes across the countries that had been part of the Soviet Union, as well as those that had used its Semashko model of health care since the Second World War. The comprehensive health reforms in these countries were driven by the fiscal collapse of the post-Soviet era, and political ideologies and coalitions driving a shift towards a more liberal economic environment.[28] This led to the introduction of payroll taxation and other earmarked taxes for replacing the tax-based system with compulsory health insurance-dominated systems in many countries.

Meanwhile, beginning in the 1980s there had been a growing understanding that PHC was underperforming and was frequently bypassed due to its perceived low quality. In the Semashko model, PHC facilities called polyclinics were staffed by specialists with basic training, had poor infrastructure and diagnostic capacity, and mainly served to dispatch patients on to higher levels of care. The introduction of mandatory insurance in some parts of the region, and cost constraints experienced in others, demanded efforts to modernise PHC and enable better gatekeeping.

Reform strategies introduced in the post-Soviet era (often involving donor funding) have been comprehensive and often included infrastructure development and training of general or family practitioners. They also involved establishing new institutional structures for the purchasing function and introducing new provider payment systems, such as capitation.[29] In some settings, newly-trained PHC (family) practitioners were given autonomy to manage resources allocated to their practices, and invest in improving services. A number of countries allowed users free choice of providers to encourage competition and quality improvement.

These changes have served to incentivise doctors to work in PHC and reflect changing societal preferences for choosing a trusted provider. These reforms proceeded over a relatively short period of time, with different degrees of success[30], demonstrating how rapid socio-political change can enable systemic PHC reforms.

Turkey's efforts to establish PHC: vision and strategic compromise

Turkey's early efforts to establish a NHI system are discussed in section 3. These attempts were blocked at different times by legislative gridlock in Parliament, opposition by the Constitutional Court, and rapid turnover of health ministers, which created instability in the policy agenda.[31] NHI was eventually introduced in 2003 by the AK party, which sought to bolster its political legitimacy by redressing inequalities and delivering on promises to its key constituencies in rural areas.[32] The success in enacting the reform relied on four political strategies: avoiding conflict, delaying action while facilitating institutional change, overpowering blockages, and achieving strategic compromise.[31]
One particular strategic compromise was achieved to avoid a Constitutional Court veto of the new social health insurance law. The ministers of health, labour, and social security mutually agreed that newly introduced social security requirements would only pertain to civil servants hired after October 2008. This compromise effectively exempted existing civil servants from being affected by the reforms, thereby limiting their opposition and ending the Constitutional Court's objections.[31]

Using Political Economy Analysis (PEA)

Undertaking PEA is a dynamic process, relevant at every stage of the policy cycle, from agenda setting to policy evaluation . Questions to guide your PEA at every stage of the policy cycle are suggested below.

Questions to guide PEA:

1. **Agenda setting:**

- Why does a policy make it or not to the agenda?
- What political levers to push and what socio- economic factors will influence policy uptake? Who and what are the political drivers of change?
- Who to associate with change (technical, political and social actors)?
- Is there political will and how to create it?

2. **Policy formulation:**
   - What words fit the political, socio and economic context?
   - What stakeholder should be involved?
   - What strategic communication is needed?
3. **Policy adaptation:**
   - Is the economic and social context supportive (e.g. is the country facing a recession or GDP growth), or should the reform wait for better conditions?
   - What social factors have an impact?
   - What changes to the policy are needed to make the reform/ change work?
4. **Policy implementation:**
   - What socio economic factors will drive or hamper implementation? (e.g. ethnic diversity in Ethiopia, high levels of inequity between rural and urban, rich and poor)?
   - How does the policy need to be implemented to work?
   - Who needs to support it?
5. **Policy evaluation:**
   - How should the policy/ reform be changed for its next iteration to better fit the political economy context? Were the right people targeted?
   - Were incentives adapted to recipients?
   - Was the right narrative developed?

The following PEA tools can be used throughout this cycle:

- **Drivers of Change and Politics of Development:** These UK Department for International Development frameworks look at the structures, institutions and agents behind particular policies to support the emergence and endurance of reforms. The Politics of Development framework subsequently supported the drivers of change approach by delving more specifically into the dynamics of the political system.[33] Applied to PHC financing, this would mean identifying which institutions and actors would support a prioritization of PHC, and which would resist it. This is focused primarily on the politics of political economy.

- **Sector level PEA** tends to start with a stakeholder mapping identifying all actors involved in the sector and their position in relation to the reform of interest; in Sierra Leone for example, civil society groups wishing to push for the removal of user fees for pregnant mother and children under five used a stakeholder and power mapping exercise to identify actors for or against this reform. This approach, however, is very political and focuses exclusively on actors, rather than on an in-depth understanding of the political economy conditions underpinning actors' appetite for change.

- **Problem-Driven Political Economy analysis** (World Bank), building from Problem Driven Iterative Analysis, focuses on identifying the reasons why a particular problem or barrier occurs, tackling it and adapting to it. These analyses tend to be

more micro and start with identifying the problem or issue to be tackled. They then recommend mapping out the institutional and governance weaknesses hindering progress; and finally identifying the political economy drivers influencing the reform.[33] Applied to PHC financing, this would imply getting to the root issue of why little attention is given to PHC financing for example, tackling this issue, and thereafter identifying and tackling a second bottleneck to change. This approach focuses on the micro level, allowing for precision in identification of the political economy drivers of problems and solutions. [34]

- **Thinking and Working Politically (TWP)** focuses on the recognition that different actors and institutions will have competing interests, and on investing in those actors and coalitions that best support the reform process of interest, in this case prioritization of PHC financing. See for example how TWP was used in the Occupied Palestinian Territories (OPTs) to understand PFM blockages.[35]

- Khan's **political settlements framework** argues that the balance of power among those who may potentially win or lose from policies (politically, economically) shapes the boundaries of feasibility in any given context and allows incentives to be fine-tuned.[36] An explicit PEA lens enables actors with less power, very often those who stand to benefit from and support equitable PHC financing – e.g. to improve accountability of frontline financing and reduce rent-seeking – to contribute to change.

- **Reich and Campos** provide a PEA guide and a software tool with an emphasis on the political domain.[37] This approach has been applied to analyse health financing reforms in Turkey and Mexico.[38]

- Some authors have examined **path dependency** of financing reform, identifying the critical junctures that explain its success or failure – e.g., in explaining China's historical hospitals dominance and deriving implications for PHC financing.[25]

References

1. Barr A, Garrett L, Marten R, Kadandale S. Health sector fragmentation: three examples from Sierra Leone. *Globalization and health* 2019; **15**(1): 1-8.
2. Raven J, Wurie H, Idriss A, et al. How should community health workers in fragile contexts be supported: qualitative evidence from Sierra Leone, Liberia and Democratic Republic of Congo. *Human Resources for Health* 2020; **18**(1): 58.
3. Raven J. The comparative agility of the community health worker cadre in fragile & conflict-affected contexts. Health Systems Global Thematic Working Group webinar; 2021; (Online): Rebuild for Resilience consortium; 2021.
4. Blecher M, Daven J, Meyer-Rath G. The COVID-19 response in South Africa, protecting lives and livelihoods: Department of Health: Republic of South Africa, 2021.
5. Kurowski C, Evans DB, Tandon A, et al. From Double Shock to Double Recovery. Washington DC: World Bank; 2021.
6. WHO. Spending on health in Europe: entering a new era. Copenhagen: WHO Regional Office for Europe, 2021.
7. IMF. Fiscal monitor: A fair shot. Washington DC: IMF, 2021.
8. Schneider P, Pivodic F, Yoo KJ. How much health financing does Sub-Saharan Africa need to fight COVID-19 (coronavirus)? 2020. https://blogs.worldbank.org/health/how-much-health-financing-does-sub-saharan-africa-need-fight-covid-19-coronavirus (accessed 10 June 2021).
9. Hanefeld J, Mayhew S, Legido-Quigley H, et al. Towards an understanding of resilience: responding to health systems shocks. *Health Policy and Planning* 2018; **33**(3): 355-67.
10. Witter S, Hunter B. Resilience of health systems during and after crises – what does it mean and how can it be enhanced? Liverpool: ReBuild Consortium, 2017.
11. WHO. Global spending on health 2020: weathering the storm. Geneva: World Health Organization, 2020.
12. Mathauer I, Dkhimi F, Townsend M. Adjustments in health purchasing as part of the Covid-19 health response: results of a short survey and lessons for the future. 2020. https://p4h.world/en/blog-covid-19-and-health-purchasing-response (accessed 1 Sept 2021).
13. Barroy H, Wang D, Pescetto C, Kutzin J. How to budget for COVID-19 response? A rapid scan of budgetary mechanisms in highly affected countries. Geneva: World Health Organization, 2020.
14. Rahim F, Allen R, Barroy H, Gores L, Kutzin J. COVID-19 Funds in Response to the Pandemic. Washington DC: International Monetary Fund, 2020.
15. Watson C, Caravani A, MItchell T, Kellett J, Peters K. Finance for reducing disaster risk: 10 things to know. London ODI and UNDP, 2015.
16. World Bank. Walking the Talk : Reimagining Primary Health Care After COVID-19. Washington DC: World Bank, 2021.
17. Miller M, Bastagli F, Hart T, et al. Financing the coronavirus response in sub-Saharan Africa. London: Overseas Development Institute, 2020.
18. Allen R. Can PFM Systems be Better Prepared for Health Emergencies: Six Lessons from the COVID-19 Crisis. 5th Meeting of the Montreux Collaborative on Fiscal Space, Public Financial Management and Health Financing. Montreaux: WHO; 2021.

19. Mathauer I. Digital technologies for health financing: what are the benefi ts and risks for UHC? Some initial reflections. Geneva: World Health Organization, 2021.
20. LSHTM Technical team. Findings from roundtable discussion on digital technology for health financing. 2021. https://www.lshtm.ac.uk/financing-phc (accessed 7 Sept 2021).
21. Patcharanarumol W, Tangcharoensathien V, Limwattananon S, et al. Why and how did Thailand achieve Good health at low cost? . In: Balabanova D MaMA, ed. Good health at low cost' 25 years on What makes a successful health system? . London: LSHTM; 2011.
22. Tangcharoensathien V, Patcharanarumol W, Kulthanmanusorn A, Saengruang N, Kosiyaporn H. The Political Economy of UHC Reform in Thailand: Lessons for Low- and Middle-Income Countries. *Health Systems & Reform* 2019; **5**(3): 195-208.
23. Wibulpolprasert S. 25 years rural doctor movement on Thailand. Bangkok: WHO; 2003.
24. Xu J, Gorsky M, Mills A. Historical roots of hospital centrism in China (1835-1949): A path dependence analysis. *Soc Sci Med* 2019; **226**: 56-62.
25. Xu J, Gorsky M, Mills A. A path dependence analysis of hospital dominance in China (1949-2018): lessons for primary care strengthening. *Health Policy Plan* 2020; **35**(2): 167-79.
26. Xu J, Yuan B. The progress and challenges in China's health financing development towards integrated people-centred health services—a systems perspective. London: Lancet Global Health Commission on Financing People-Centred Primary Health Care (Available at https://www.lshtm.ac.uk/financing-phc), 2021.
27. Page SE. Path dependence. *Quarterly Journal of Political Science* 2006; **1**(1): 87-115.
28. Sheiman I, Langenbrunner J, Kehler J, Cashin C, Kutzin J. Sources of funds and revenue collection: reforms and challenges. In: Kutzin J, Cashin C, Jakab M, editors. Implementing health financing reform: Lessons from countries in transition. Copenhagen: WHO Regional Office for Europe; 2010.
29. Fuenzalida-Puelma HL, O'Dougherty S, Evetovits T, Cashin C, Kacevicius G, McEuen M. Purchasing of health care services. In: Kutzin J, Cashin C, Jakab M, editors. Implementing health financing reform: Lessons from countries in transition. Copenhagen: WHO Regional Office for Europe; 2010.
30. Kutzin J, Cashin C, Jakab M. Implementing health financing reforms: lessons from countries in transition Copenhagen: WHO Regional Office for Europe; 2010.
31. Sparkes SP, Bump JB, Reich MR. Political Strategies for Health Reform in Turkey: Extending Veto Point Theory. *Health Systems & Reform* 2015; **1**(4): 263-75.
32. Baris E, Mollahaliloglu S, Aydin S. Healthcare in Turkey: from laggard to leader. *BMJ* 2011; **342**: c7456.
33. DFID. Political Economy Analysis - How To Note DFID, 2009.
34. Fritz V, Levy B, Ort R. Problem-Driven Political Economy Analysis: The World Bank's Experience. Directions in Development. Washington, DC: World Bank, 2014.
35. McCulloch N, Piron L-H. Thinking and Working Politically: Learning from practice. Overview to Special Issue. *Development Policy Review* 2019; **37**(S1): O1-O15.
36. Khan M. Political settlements and the governance of growth-enhancing institutions. SOAS University of London; 2010.
37. Reich MR, Campos PA. A Guide to Applied Political Analysis for Health Reform. 2020. https://cdn1.sph.harvard.edu/wp-content/uploads/sites/2216/2020/08/Guide-Applied-Political-Analysis-final-2020.08.29-FINAL.pdf (accessed 1 Sept 2021).
38. Sparkes SP, Bump JB, Özçelik EA. Political Economy Analysis for Health Financing Reform,. *Health Systems & Reform* 2019: 183-94.
